# Neuronal DNA damage response‐associated dysregulation of signalling pathways and cholesterol metabolism at the earliest stages of Alzheimer‐type pathology

**DOI:** 10.1111/nan.12252

**Published:** 2015-07-07

**Authors:** Julie E. Simpson, Paul G. Ince, Thais Minett, Fiona E. Matthews, Paul R. Heath, Pamela J. Shaw, Emily Goodall, Claire J. Garwood, Laura E. Ratcliffe, Carol Brayne, Magnus Rattray, Stephen B. Wharton, the MRC Cognitive Function and Ageing Neuropathology Study Group

**Affiliations:** ^1^ Sheffield Institute for Translational NeuroscienceUniversity of SheffieldSheffieldUK; ^2^ Institute of Public HealthUniversity of CambridgeCambridgeUK; ^3^ Department of RadiologyUniversity of CambridgeCambridgeUK; ^4^ MRC Biostatistics UnitInstitute of Public HealthCambridgeUK; ^5^ Institute of Health and SocietyUniversity of NewcastleNewcastleUK; ^6^ Faculty of Life SciencesUniversity of ManchesterManchesterUK

**Keywords:** Ageing brain, DNA damage response, neurones, microarray, dementia, Alzheimer's

## Abstract

**Aims:**

Oxidative damage and an associated DNA damage response (DDR) are evident in mild cognitive impairment and early Alzheimer's disease, suggesting that neuronal dysfunction resulting from oxidative DNA damage may account for some of the cognitive impairment not fully explained by Alzheimer‐type pathology.

**Methods:**

Frontal cortex (Braak stage 0–II) was obtained from the Medical Research Council's Cognitive Function and Ageing Study cohort. Neurones were isolated from eight cases (four high and four low DDR) by laser capture microdissection and changes in the transcriptome identified by microarray analysis.

**Results:**

Two thousand three hundred seventy‐eight genes were significantly differentially expressed (1690 up‐regulated, 688 down‐regulated, *P* < 0.001) in cases with a high neuronal DDR. Functional grouping identified dysregulation of cholesterol biosynthesis, insulin and Wnt signalling, and up‐regulation of glycogen synthase kinase 3β. Candidate genes were validated by quantitative real‐time polymerase chain reaction. Cerebrospinal fluid levels of 24(S)‐hydroxycholesterol associated with neuronal DDR across all Braak stages (*r*_s_ = 0.30, *P* = 0.03).

**Conclusions:**

A persistent neuronal DDR may result in increased cholesterol biosynthesis, impaired insulin and Wnt signalling, and increased GSK3β, thereby contributing to neuronal dysfunction independent of Alzheimer‐type pathology in the ageing brain.

## Introduction

Population‐based studies have shown that age‐associated cognitive decline often occurs in individuals with low levels of either classical Alzheimer‐type neuropathology or other neurodegenerative pathologies [1]. Oxidative damage to nucleic acids is evident in mild cognitive impairment (MCI) and early Alzheimer's disease (AD) [2, 3, 4], suggesting that oxidative stress is an early contributor to neuronal dysfunction, which either independently of, or interacting with, early Alzheimer‐type pathology results in cognitive impairment. Oxidative DNA damage induces a DNA damage response (DDR), which is characterized by the activation of DNA–protein kinase catalytic subunit (DNA–PKcs) and the phosphorylation of the histone H2AX (γH2AX) [5]. Previous investigation of the Medical Research Council's Cognitive Function and Ageing Study (MRC CFAS) cohort has demonstrated high levels of oxidative stress and an associated DDR occur at the earliest stages of Alzheimer pathology [6], as defined by low Braak and Braak neurofibrillary tangle stage [7], and an increased neuronal DDR at these early pathological stages is associated with cognitive impairment and neuronal senescence [8].

Various candidate mechanisms may underlie neuronal vulnerability and initiate adverse cellular processes several years before eventual cognitive decline. Signalling dysregulation has been implicated in the pathogenesis of AD [9, 10], and large‐scale genome‐wide association study (GWAS) studies have identified potential novel pathways [11]; however, the contribution of these cell processes to cognitive impairment remains to be defined. Population‐based neuropathology studies allow unbiased assessment of the relationship of cellular and molecular pathologies to dementia and enables early stages of classical pathologies to be investigated. By identifying novel associations, these population‐based approaches have the potential to identify new biomarkers and therapeutic strategies [12, 13].

Having demonstrated the population variation in the neuronal DDR in individuals with little or no Alzheimer‐type pathology and the association with cognitive impairment [8], this study aimed to identify the gene expression changes associated with the neuronal DDR, which may contribute to neuronal dysfunction in the ageing brain. Laser capture microdissection (LCM) of neurones from human post‐mortem material combined with microarray technology enables the neuronal transcriptome of samples to be simultaneously assessed, and changes in gene expression identified. Although a number of studies have reported microarray data in normal ageing and AD [14, 15, 16, 17, 18], to date, no study has specifically characterized the gene expression profile of neurones with high levels of a DDR (DNA–PKcs and γH2AX) at low Braak and Braak stages. Our findings identify a major transcriptional response with potentially important implications for neuronal dysfunction and cognitive decline independent of classical Alzheimer‐type pathology in the ageing brain.

## Materials and methods

### Human CNS tissue

CNS tissue was obtained from MRC CFAS, following Research Ethics Committee approval (REC ref 12/EM/0118). The structure of the CFAS neuropathology cohort has been previously described and reviewed [10]. Brains were dissected following a standard protocol [19] and neuropathological lesions assessed as part of the core CFAS neuropathology study using a modified protocol from the Consortium to Establish a Registry of Alzheimer's Disease (CERAD) [20] (http://www.cfas.ac.uk). Braak and Braak stage 0–II, frozen frontal cortex (FCx) was available for 39 cases with an average age at death of 82 years (range 70–102 years), median post‐mortem delay of 17.5 h [interquartile range (IQR 10–24 h] and tissue pH 6.6 (IQR pH 6.3–6.9). Individuals in the study were regularly interviewed and underwent extensive cognitive assessment [1, 21]. Dementia status at death was determined on the basis of all information available for each participant [22]. Within this cohort, 14 participants had dementia, 22 had no dementia and 3 participants had an unknown dementia status at death due to the lack of information in the years preceding death.

### Cerebrospinal fluid (CSF)

CSF was collected post‐mortem from one centre of the CFAS cohort (98 cases), centrifuged at 2000 g for 10 min, aliquoted and stored at −80°C until analysis. The cohort comprised 30 cases Braak and Braak stages 0–II [mean (standard deviation)], [80.5 (7.0)], 50 cases Braak and Braak stages III–IV [88.6 (6.5)], and 18 cases Braak and Braak stage V–VI [86.3 (6.1)]. The neuronal and astrocyte DDR in these cases was previously assessed [6], and all cases were previously screened for ApoE genotype [23]. Only one participant reported taking statins at their last interview.

### LCM of neurones

**Figure 1:**
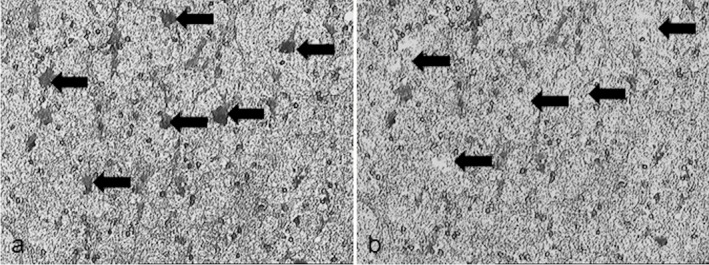
Laser capture microdissection (LCM) of neurones. Toludine blue positive neurones were isolated (**a**) before and (**b**) after LCM, as indicated by the arrows.

Gene expression analysis was performed on neurones isolated from 10 cases (5 high neuronal DDR and 5 low DDR) using laser capture microscopy (LCM) (Figure 1), based on previous investigation of the neuronal DDR (DNA–PKcs and γH2AX) in this cohort [8]. Details of cases used in the microarray study are shown in Table 1. Validation experiments were performed on LCM‐isolated neurones from the remaining 29 CFAS cases. Frozen sections (7 μm) were fixed in ice cold acetone for 3 min and stained with Toludine Blue for 1 min. Sections were dehydrated in a graded series of ethanol (70, 95 and 100%), extensively cleared in xylene and air dried for 1 h. Approximately, 1000 pyramidal neurones were isolated from each case using a PixCell II LCM system (Life Technologies, Paisley, UK). Total RNA was extracted using the PicoPure RNA extraction kit according to the manufacturer's protocol (Life Technologies), with typical yields of 100 ng of RNA. The quantity (NanoDrop 1000 spectrophotometer, Thermo Scientific, Wilmington, DE USA) and quality (Agilent Bioanalyser 2100, RNA 6000 Pico LabChip; Agilent, Palo Alto, CA, USA) of the starting RNA were analysed. Sterile solutions made with diethylpyrocarbonate (DEPC)‐treated water and RNAse‐free conditions were used throughout this protocol.

**Table 1:** MMSE score, gender, Aβ neuropathology, proportion of neurones (%) with a DDR and RIN of cases used in the microarray study

MMSE	Sex	Diffuse plaque score	Neuritic plaque score	γH2AX	DNA–PKcs	RIN
Low DDR						
**30**	**M**	**2**	**0**	**20**	**37**	**5.3**
26	M	0	0	31	18	2.6
23	M	0	0	22	35	2.6
30	F	1	0	52	38	5.3
26	M	0	0	40	29	3.3
High DDR						
6	F	2	0	44	100	2.3
20	M	0	0	75	91	5.3
**0**	**F**	**1**	**0**	**73**	**88**	**4.3**
21	M	1	0	74	72	4.3
20	F	0	0	72	80	2.5

Cases in bold were removed from the final analysis: low‐DDR cases sample outlier, high DDR case with Pick's disease.

DDR, DNA damage response; DNA–PKcs, DNA–protein kinase catalytic subunit; RIN, RNA integrity number.

### RNA amplification and microarray hybridization

Total RNA was annealed to an oligo‐d(T) primer with a T7 polymerase binding site. After generation of double‐stranded cDNA, copy RNA (cRNA) was transcribed which then formed the RNA template for a second round of amplification. At the end of this round, after synthesis of double‐stranded cDNA, biotin‐labelled cRNA was prepared using the Affymetrix Gene Chip (Affymetrix, Santa Clara, CA, USA) *in vitro* transcription labelling kit. Following clean‐up of the biotin‐labelled cRNA the material was assayed (Agilent Bioanalyser 2100) to ensure sufficient RNA of appropriate quality had been prepared. Labelled cRNA (12.5 μg) was fragmented and applied to HGU133 Plus 2.0 gene microarrays and hybridized over 16 h at 45°C in a rotating oven at 60 rpm. Post hybridization washing and sample staining was carried out using the Fluidics Station 400 and the Gene Chip Operating System. Gene chips were scanned using the GC3000 7G scanner and data processed for quality control using Expression Console software (Affymetrix) and further analysis was carried out using Qlucore Omics Explorer (Qlucore, Lund, Sweden).

### Microarray analysis

The Robust Multi‐array Average (RMA) algorithm was used for data normalization and univariate and principal component analyses to determine intensity distribution and eliminate sample outliers [24]. Significant differentially expressed genes (*P* < 0.001) were analysed using Qlucore Omics Explorer. The Database for Annotation Visualisation and Integrated Discovery bioinformatics programme (DAVID) was used to group genes according to their function and to assign genes to specific functional pathways [25].

### Validation of microarray data: quantitative real‐time polymerase chain reaction (qPCR)

RNA was extracted from LCM‐ed neurones in additional frozen FCx cases, which had not been used for the microarray studies. qPCR was performed using IDT PrimeTime qPCR assays (Integrated DNA Technologies, Glasgow, UK) containing 50 ng cDNA, 500 nM primers, 250 nM probe, and Brilliant qPCR mix (Agilent, Stockport, UK) in a total volume of 10 μl, for more details of primer sequences see Table 2. Following denaturation at 95°C for 10 min the products were amplified (40 cycles at 95°C for 30 s and 60°C for 60 s) using an MX3000P RT PCR System (Stratagene, Paisley, UK). β‐actin was amplified on each plate to normalize expression levels of target genes between different samples using the ΔΔCt calculation (ABI) and to assess assay reproducibility.

**Table 2:** Quantitative real‐time polymerase chain reaction primer/probe sequence

Gene		Sequence
DHCR24	Probe	5′‐FAM‐CATCTGGAAGCCATGCACGCTG‐3′
	Primer 1	5′‐GTACAAGGAGCCATCAAACATC‐3′
	Primer 2	5′‐AGGCAGCTGGAGAAGTTTG‐3′
*GSK3B*	Probe	5′‐FAM‐ACCACTCAAGAACTGTCAAGTAATCCACC‐3′
	Primer 1	5′‐ACGGGACCCAAATGTCAAAC‐3′
	Primer 2	5′‐GAGGAGGAATAAGGATGGTAGC‐3′
*HMGCR*	Probe	5′‐FAM‐ACCAACCTACTACCTCAGCAAGCCT‐3′
	Primer 1	5′‐TCCTTGAACACCTAGCATCTG‐3′
	Primer 2	5′‐CTGCACCATGCCATCTATAGAG‐3′
*IGFR1*	Probe	5′‐FAM‐TGAGGCCTTCCTTCCTGGAGATCA‐3′
	Primer 1	5′‐CTCCATCTCCTCTTTGATGCTG‐3′
	Primer 2	5′‐CCAGACAACTGTCCTGACAT‐3′
*INSR*	Probe	5′‐FAM‐CTCCATCCATGACAAATTTCAACACCTGT‐3′
	Primer 1	5′‐GTTGTCGGGTTGATCCAGAT‐3′
	Primer 2	5′‐AAATCACCAGCTTGGCAGA‐3′
*NFL*	Probe	5′‐FAM‐AGGAAGAGGAGGCAGCTGAAGAGG‐3′
	Primer 1	5′‐AGGAGGAGAAGGACAAGGA‐3′
	Primer 2	5′‐TTGGTTTCCTCTCCTTCTTCAC‐3′
*WNT3*	Probe	5′‐FAM‐TGGTGCCCTACTTGCAGGTGTG‐3′
	Primer 1	5′‐CCAGGAGTGTATTCGCATCT‐3′
	Primer 2	5′‐ATGAGACTTCGCTGAATCCG‐3′
*ACTB*	Probe	5′‐FAM‐CCATGTACGTTGCTATCCAGGCTGT‐3′
	Primer 1	5′‐CCAGTGGTACGGCCAGA‐3′
	Primer 2	5′‐GCGAGAAGATGACCCAGAT‐3′

*ACTB*, actin, beta; DHCR24, 24‐dehydrocholesterol reductase; *GSK3B*, glycogen synthase kinase 3β; *HMGCR*, HMG–CoA reductase; *IGFR1*, insulin growth factor receptor 1; *INSR*, insulin receptor; *NFL*, neurofilament light.

### Immunohistochemistry

To confirm neuronal expression of proteins encoded by the candidate genes, immunostaining was performed using a standard avidin biotinylated enzyme complex (ABC) method, and the signal visualized with diaminobenzidine (Vector Laboratories, Peterborough, UK). A summary of the primary antibodies used is shown in Table 3.

**Table 3:** Antibody source and specificity

Antibody	Isotype	Dilution (time, temp)	Supplier
NFL	Rabbit IgG	1:50 (60 min, RT)	AbCam, UK
GSK3B	Rabbit IgG	1:10 (60 min, RT)	Sigma, UK
HMGCR	Rabbit IgG	1:100 (60 min, RT)	AbCam, UK
IRS1	Rabbit IgG	1:50 (60 min, RT)	Santa Cruz, UK
SREBP2	Rabbit IgG	1:100 (60 min, RT)	AbCam, UK
Wnt‐3a	Rabbit IgG	1:50 (60 min, RT)	AbCam, UK

GSK3B, glycogen synthase kinase 3β; HMGCR, HMG–CoA reductase; IgG, immunoglobulin G; IRS1, insulin receptor substrate 1; RT, room temperature; SREBP, sterol regulatory element binding proteins.

### Western blotting

Frozen FCx from the array cases was homogenized in Tris extraction buffer (10 mM Tris‐HCl pH 7.4, 0.8M sodium chloride, 1 mM ethylene glycol tetraacetic acid (EGTA), 10% sucrose, 0.1 mM phenylmethanesulphonyl fluoride (PMSF), 2 μg/ml aprotonin, 10 μg/ml leupeptin, 5 μg/ml pepstatin, 40 mM β‐glycerophosphate, 50 mM sodium fluoride, 200 μM sodium orthovanadate) and centrifuged at 14 000 rpm for 30 min at 4°C. The protein content of the supernatant was measured using the bicinchoninic acid method and equal protein amounts (20 μg) analysed by Western blot analysis. Proteins were separated by sodium dodecyl sulphate‐polyacrylamide gel electrophoresis and transferred to nitrocellulose (BioRad Laboratory, Hemel Hempstead, UK). Membranes were incubated overnight with anti‐neurofilament light (NFL; 1:2500; New England Biolabs, Hitchin, UK) or anti‐Wnt3 (1:500; Abcam, Cambridge, UK) followed by the appropriate horseradish peroxidise‐linked secondary antibody (1:1000) and visualized by enhanced chemiluminescence detection. To confirm equal protein loading, the membrane was reprobed for β‐actin (1:5000; Abcam). Protein expression levels were determined by densitometry of the appropriate, subsaturated band using G:BOX Chemi (Syngene, Cambridge, UK) and the results normalized to β‐actin.

### 24(S)‐hydroxycholesterol elisa

Levels of 24(S)‐OHC were quantitated by enzyme linked immunosorbent assay (elisa) (Enzo Life Sciences UK Ltd, Exeter, UK) in all available CSF from one centre of the CFAS cohort (98 cases), according to the manufacturer's instructions.

### Statistical analysis

Spearman's correlation coefficient was calculated to test associations between continuous variables. Analyses were performed and graphs obtained using IBM® spss® Statistics 21 (Armonk, NY, USA) and statistical package stata, version 12 (College Station, TX, USA). To test the possibility of 24(S)‐OHC levels as a risk factor for dementia, logistic regression was used, and to test if possession of the ApoEε4 allele was a risk factor for decreased 24(S)‐OHC, we used multiple linear regression analysis with 24(S)‐OHC as a dependent variable. All analyses were adjusted by age at death and sex. All tests were two‐tailed, 95% confidence intervals (CI) were calculated for the odds ratio (OR) and for the linear regression coefficients (β).

## Results

### DDR‐associated changes in the neuronal transcriptome

The transcription profiles of laser‐captured neurones from high‐ and low‐DDR cases were generated using Human Genome U133 Plus 2.0 Arrays, which comprise 1.3 × 10^6^ unique oligonucleotide sequences, including >47 000 transcripts and variants of 33 000 genes. Between 32% and 45% of the probe set sequences were present across all samples [mean (range)]: low neuronal DDR [36% (32–45%)]; high neuronal DDR [40% (37–43%)].

**Figure 2:**
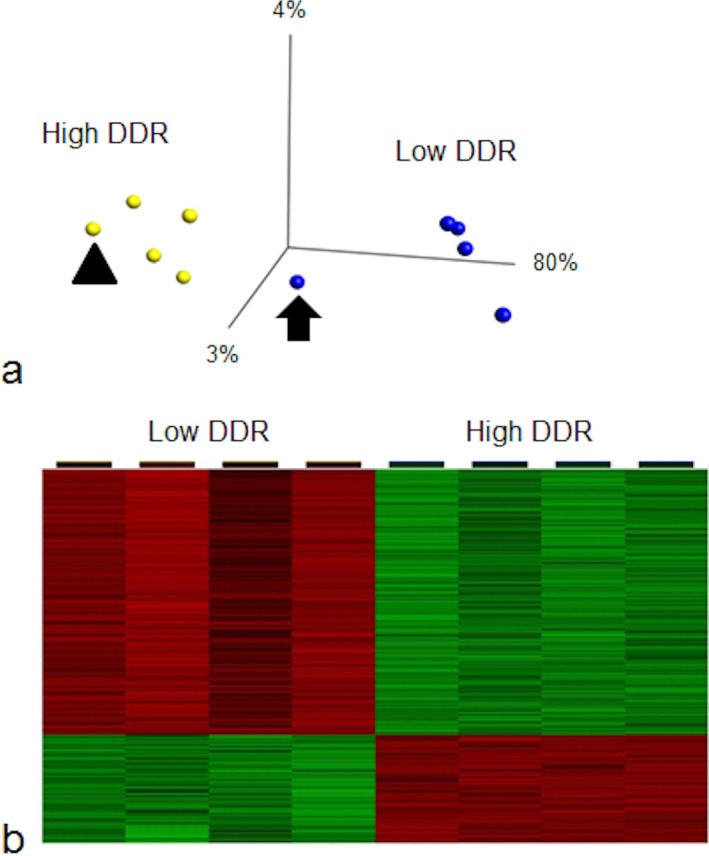
Gene expression analysis of cases with a high neuronal DNA damage response (DDR). (**a**) Principal component analysis of microarray data. One sample outlier in the low‐DDR group (indicated by the arrow) and one Pick's disease case in the high DDR group (indicated by the arrow head) were removed from subsequent analysis. (**b**) Heat map depicting up‐regulated (green) and down‐regulated (red) gene expression changes (*P* < 0.001). A high neuronal DDR was associated with the up‐regulation of 1690 genes and the down‐regulation of 688 genes.

Gene expression analyses are sensitive to the presence of sample outliers, therefore rigorous quality control procedures were used to ensure the highest possible level of quality for both datasets. One low‐DDR sample outlier was identified by visual inspection after clustering the samples using hierarchical clustering and one high DDR case with Pick's disease was excluded on review of the neuropathology, both cases were removed from subsequent analysis (Figure 2). The RMA was re‐analysed after these two cases were removed and principal components analysis was performed on the significant, differentially expressed genes (the expression dataset is freely available at Gene Expression Omnibus, accession number GSE66333). Transcript clusters showed no association with age, gender, post‐mortem delay or pH.

Two thousand three hundred seventy‐eight genes (1690 up‐regulated, 688 down‐regulated) were significantly differentially expressed (*P* < 0.001) in cases with a high DDR (Supplementary Tables 1 and 2). Functional grouping analysis identified the dysregulation of genes associated with the cellular response to stress, the synapse, signalling, ubiquitin‐mediated proteolysis, transport, the cytoskeleton and transcription. Pathway analysis identified significant up‐regulation of Wnt signalling transcripts (*P* = 0.004) and down‐regulation of insulin signalling associated transcripts (*P* = 0.029) in cases with a high neuronal DDR.

*NFL* transcripts had the greatest fold change (FC) (*NFL* probe set 221805_at, FC = 36.03, *P* = 0.0002; probe set 221801_x_at, FC = 13.87, *P* = 0.0001; probe set 221916_at, FC = 5.89, *P* = 0.0001). Glycogen synthase kinase 3β (*GSK3B*) transcripts were also significantly up‐regulated in cases with a high neuronal DDR (probe set 226191_at, FC = 4.19, *P* = 0.00004).

### DDR‐associated dysregulation of insulin signalling pathway transcripts

Further interrogation of the microarray dataset with less stringent parameters (*P* < 0.05) confirmed the neuronal DDR‐associated dysregulation of insulin signalling and identified insulin signalling‐associated changes in gene expression, including down‐regulation of insulin receptor (*IR* probe set 213792_at, FC = −1.05, *P* = 0.049), insulin growth factor receptor 1 (*IGFR1* probe set 203628_at, FC = −1.1, *P* = 0.013), insulin growth factor 2 (*IGF2* probe set 202410_at, FC = −1.1, *P* = 0.022 and probe set 210881_at, FC = −1.1, *P* = 0.007), insulin growth factor binding protein 5 (*IGFBP5* probe set 203425_at, FC = −1.1, *P* = 0.0016), phosphoinositide‐3‐kinase CD (*PIK3CD* probe set 211230_at, FC = −1.2, *P* = 0.031) and up‐regulation of insulin degrading enzyme (*IDE* probe set 203327, FC = 1.1, *P* = 0.006).

### DDR‐associated activation of the cholesterol biosynthesis pathway

Analysis of the transcription profiles of laser‐captured neurones from high neuronal DDR cases compared with low neuronal DDR at the less stringent parameters (*P* < 0.05) also identified activation of the cholesterol synthesis pathway, namely significant overexpression of six of the 10 genes encoding enzymes driving the biosynthesis of cholesterol from acetyl CoA: acetoacetyl‐CoA transferase 1 (*ACAT1* probe set 205412_at, FC = 1.2, *P* = 0.004), HMG–CoA synthase (*HMGCS1* probe set 221750_at, FC = 1.1, *P* = 0.008), HMG–CoA reductase (*HMGCR* probe set 202539_s_at, FC = 4.5, *P* = 0.0008 and probe set 202540_s_at, FC = 1.2, *P* = 0.005), farnesyl diphosphate synthase (*FDPS* probe set 201275_at, FC = 1.1, *P* = 0.002), squalene synthase (*FDFT1* probe set 210950_s_at, FC = 1.1, *P* = 0.012) and 24‐dehydrocholesterol reductase (DHCR24 probe set 200862_at, FC = 1.2, *P* = 0.005). In the brain, cholesterol biosynthesis is regulated by sterol regulatory element binding factors (SREBF), also known as sterol regulatory element binding proteins (SREBP). A high neuronal DDR was associated with the down‐regulation of *SREBF1* (probe set 1558875_at, FC = −1.46, *P* = 0.0004) and *SREBF2* (probe set 201247_at, FC = −1.1, *P* = 0.050; probe set 242748_at, FC = −1.1, *P* = 0.001).

### Validation of microarray candidates

Candidate genes playing key roles in the pathways of interest or which showed significant altered expression were validated by qPCR on LCM‐isolated neurones from all additional cases from the CFAS Braak and Braak stage 0–II cohort, which contained high‐quality RNA as indicated by the absence of excessive degradation of the ribosomal RNA (19/29 additional cases). Decreasing expression of *IR* (*r*_s_ = −0.565, *P* = 0.028) and *IGFR1* (*r*_s_ = −0.704, *P* = 0.004), and increasing expression of *NFL* (*r*_s_ = 0.633, *P* = 0.004), *GSK3B* (*r*_s_ = 0.572, *P* = 0.011), wingless‐type MMTV integration site family, member 3a (*WNT3A*, *r*_s_ = 0.539, *P* = 0.017), *HMGCR* (*r*_s_ = 0.450, *P* = 0.053) and DHCR24 (*r*_s_ = 0.458, *P* = 0.049) were associated with an increasing neuronal DDR (proportion of γH2AX^+^ neurones) (Table 4).

**Table 4:** Neuronal DNA damage response**‐**associated gene changes

Gene	Microarray		qPCR	
	FC	*P*‐value	*r*_s_	*P*‐value
*NFL*	36.03	0.0002	0.633	0.004
*GSK3B*	4.19	0.00004	0.572	0.011
*IR*	−1.05	0.049	−0.565	0.028
*IGFR1*	−1.1	0.013	−0.704	0.004
*WNT3A*	2.66	0.0009	0.539	0.017
*HMGCR*	4.5	0.0008	0.450	0.053
DHCR24	1.2	0.005	0.458	0.049

DHCR24, 24‐dehydrocholesterol reductase; FC, fold change; *GSK3B*, glycogen synthase kinase 3β; *HMGCR*, HMG–CoA reductase; *IGFR1*, insulin growth factor receptor 1; *IR*, insulin receptor; qPCR, quantitative real‐time polymerase chain reaction; *r*_s_, Spearman's rho correlation coefficient.

**Figure 3:**
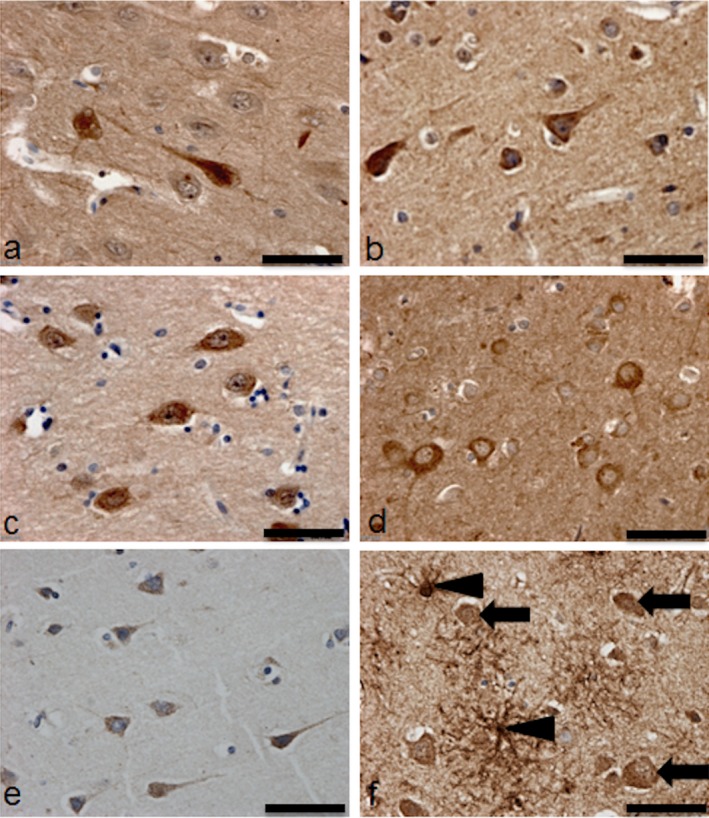
Immunohistochemical assessment of protein expression encoded by candidate genes. Neuronal expression of (**a**) neurofilament light (NFL), (**b**) IRS1, (**c**) glycogen synthase kinase 3β (GSK3β), (**d**) Wnt‐3a and (**e**) HMG–CoA reductase (HMGCR). (**f**) sterol regulatory element binding proteins (SREBP)2 was associated with both neurones (arrow) and astrocytes (arrow head). Scale bar represents 50 μm.

**Figure 4:**
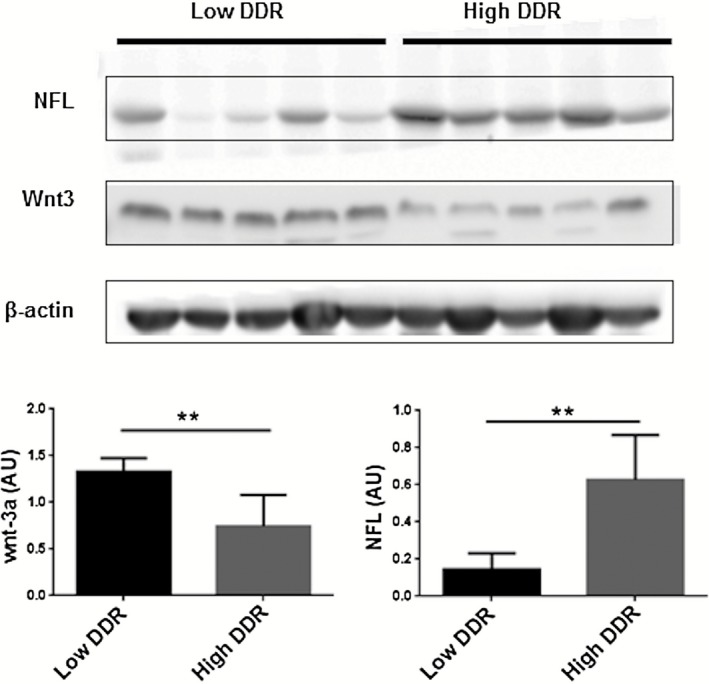
Protein expression encoded by candidate genes. Western blot analysis of total protein extracts demonstrated significant up‐regulation of neurofilament light (NFL) (68 kDa) and down‐regulation of Wnt‐3a (39 kDa) in cases with a high neuronal DNA damage response (DDR). **P < 0.01. AU, arbitrary units.

Neuronal expression of the protein encoded by selected candidate genes, namely NFL (Figure 3 **a**), insulin receptor substrate 1 (IRS1) (Figure 3 **b**), GSK3β (Figure 3 **c**), Wnt‐3a (Figure 3 **d**), HMGCR (Figure 3 **e**) and SREBP2 (Figure 3 **f**) was confirmed by immunohistochemistry. Furthermore, significantly higher levels of NFL (*P* = 0.003) and significantly lower levels of Wnt3a (*P* = 0.007) were associated with a high neuronal DDR, as detected by Western blotting of protein extracts from the array cases (Figure 4).

### CSF levels of 24(S)‐OHC associate with the neuronal DDR and inversely associate with Braak and Braak stage

**Figure 5:**
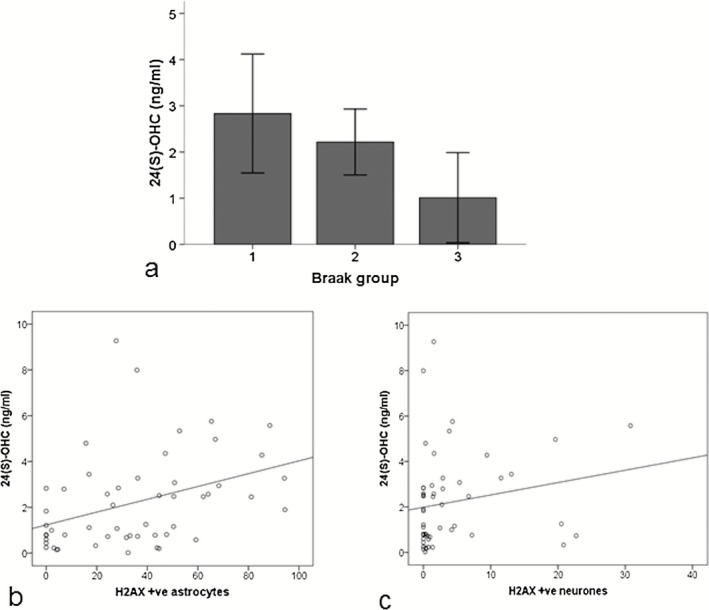
Cholesterol turnover correlates with increased levels of both astrocyte and neuronal DNA damage response (DDR) and inversely associates with Braak stage. Cerebrospinal fluid (CSF) levels of 24(S)‐OHC (**a**) inversely associated with increasing Braak and Braak stage, and positively associated with both (**b**) an astrocytic DDR (*r*_s_ = 0.43, *P* = 0.001)and (**c**) a neuronal DDR (*r*_s_ = 0.30, *P* = 0.033). Braak group 1 = Braak and Braak stage 0–II, Braak group 2 = Braak and Braak stage III–IV, Braak group 3 = Braak and Braak stage V–VI. AU = arbitrary units.

As cholesterol plays a key role in synapse formation and function [26], the study was extended to assess CSF levels of 24(S)‐OHC across all Braak and Braak stages in all available CSF samples from one well‐characterized centre of the CFAS cohort [6]. Use of this larger cohort enabled the association between cholesterol turnover and the DDR, Braak and Braak stage, ApoEε4 genotype and cognitive impairment to be investigated. CSF levels of 24(S)‐OHC negatively correlated with increasing Braak and Braak stage (*r*_s_ = −0.29, *P* = 0.040) (Figure 5 **a**): Braak stage 0–II (median 2.58 ng/ml; minimum–maximum: 0.16–9.28), Braak stage III–IV (1.90 ng/ml; 0.15–7.99) and Braak stage V–VI (0.63 ng/ml; 0.02–4.35). Levels of 24(S)‐OHC significantly correlated with both an astrocyte DDR (*r*_s_ = 0.43, *P* = 0.001) and a neuronal DDR (*r*_s_ = 0.30, *P* = 0.033) across all Braak and Braak stages (Figure 5 **b,c**).

Levels of 24(S)‐OHC were not a significant risk factor for clinical dementia (OR = 1.12, 95% CI 0.82; 1.53, *P* = 0.491). Possession of at least one ApoEε4 allele was not significantly associated with increased levels of 24(S)‐OHC (β = 0.49, 95% CI −0.77; 1.75, *P* = 0.435). CSF levels of 24(S)‐OHC were weakly associated with brain pH (*r*_s_ = 0.14, *P* = 0.341) and post mortem delay (PMD) (*r*_s_ = −0.12, *P* = 0.402).

## Discussion

Age‐associated cognitive decline often occurs in individuals with low levels of conventional dementia‐associated pathologies (Aβ plaques, neurofibrillary tangles, vascular lesions, synucleinopathy) [1, 27]. Braak and Braak staging describes an anatomical progression of neurofibrillary tangles, in which tangles that are restricted to mesial temporal lobe structures may precede clinico‐pathological AD by many years [7]. The current study investigated prefrontal cortex as it is affected relatively late in the AD process, thereby allowing us to identify potential gene expression changes at early Braak and Braak stages before the onset of classical AD‐associated neuropathological changes. We have previously demonstrated an association between the neuronal DDR and cognitive impairment in cases with little or no Alzheimer's neuropathology, and shown that high levels of a neuronal DDR correlate with lower Mini Mental State Examination (MMSE) scores [8]. The present population‐based study extends this previous finding and demonstrates that the neuronal response to DNA damage at the earliest stages of Alzheimer's pathology (Braak and Braak stage 0–II) is associated with dysregulation of intracellular signalling pathways and cholesterol biosynthesis, which may contribute to neuronal dysfunction and cognitive impairment independent of established Alzheimer pathology in the ageing brain.

Insulin resistance has been linked to several neurodegenerative diseases, including AD [9, 28, 29, 30, 31, 32]. Low concentrations of insulin, reduced receptor density and dysregulation of insulin signalling have all been reported in AD [33, 34] and with increasing Braak and Braak stage in the ageing brain [10, 35]. Furthermore, administration of insulin has been shown to improve memory in AD patients [36] suggesting a potential therapeutic avenue for dementia treatment. In the present transcriptomic study a high neuronal DDR was associated with the dysregulation of insulin/IGF and Wnt signalling pathways. Defects in both signalling pathways have been postulated to contribute to the pathogenesis of AD and dementia [9, 37, 38]. Although pathway analysis of the microarray data identified significant up‐regulation of Wnt signalling associated transcripts in cases with a high neuronal DDR, protein expression was significantly lower in these cases, suggesting post‐transcriptional regulation of Wnt expression. Both insulin/IGF and Wnt signalling negatively regulate the activity of GSK3β [39], expression of which was significantly up‐regulated in cases with a high neuronal DDR. The GSK3 hypothesis of AD proposes that GSK3β over‐activation plays a key role in the hyperphosphorylation of tau, increased β‐amyloid production and neuronal dysfunction [40]. We propose that increased levels of oxidative stress lead to a neuronal DDR, which is associated with the down‐regulation of insulin/IGF and Wnt signalling, resulting in increased GSK3β activity and ultimately leading to neuronal dysfunction and cognitive impairment, independent of local Alzheimer‐type pathology in the ageing brain.

Increasing evidence suggests AD represents a metabolic disease in which brain glucose utilization and energy production are impaired [32, 33, 34, 41]. Dysfunction of the insulin/IGF signalling pathway impacts neuronal growth, survival, differentiation, synapse formation, energy metabolism, gene expression, protein synthesis and cytoskeletal assembly [31]. Cytoskeletal disruption is a prominent feature of neurodegenerative diseases, including AD [42, 43]. Expression of NFL was significantly up‐regulated in cases with a high neuronal DDR, both at the gene (ranging from 5‐ to 36‐fold increase) and protein levels (fourfold increase), suggesting that abnormal accumulation of this cytoskeletal protein may contribute to neuronal dysfunction by impacting axonal transport.

Oxidative stress is one of the earliest events in AD, with implications as an important mediator in the onset, progression and pathogenesis of the disease [2, 3, 4]. The proposed sources of oxidative stress include abnormal mitochondria, redox transition metals and oxysterols [44, 45]. In the brain, cholesterol is converted to the oxysterol 24(S)‐OHC, which is considered a marker of cholesterol turnover [46, 47], and which is elevated in the CSF of patients with MCI [48, 49, 50]. In the current study, CSF levels of 24(S)‐OHC were highest in cases with little or no Alzheimer‐type pathology, and correlated with the neuronal DDR. We propose that high levels of the pro‐oxidant 24(S)‐OHC are one potential source of oxidative stress, which may contribute to an elevated neuronal response to oxidative DNA damage, resulting in the activation of the neuronal cholesterol biosynthesis pathway to maintain axons, dendrites and synapses at low Braak and Braak stages [26, 51]. However, while increased cholesterol biosynthesis may preserve neuronal integrity at low Braak stages, high levels of 24(S)‐OHC may also promote the progression of tau pathology [52] and increase Aβ neurotoxicity [45] in the ageing brain. We also demonstrate a significant reduction in 24(S)‐OHC expression associated with increasing Braak stage in the ageing brain, supporting the hypothesis that cholesterol turnover is highest during early neurodegenerative changes [53], and suggesting that this neuroprotective response fails with disease progression.

The pathogenesis of AD is linked to cholesterol metabolism, and is associated with genetic risk factors including apolipoprotein E (*ApoE*) genotype and polymorphisms in *HMGCR* [54, 55, 56, 57]. However, no association between ApoE genotype and 24(S)‐OHC was detected in this study, although it should be noted that the sample size for the CSF study was small. In the brain, cholesterol is almost exclusively derived from endogenous biosynthesis, and is regulated by the transcription factor SREBP2, which controls expression of the enzymes involved in cholesterol synthesis, including the rate‐limiting enzyme HMGCR [58]. Although neurones are capable of synthesizing their own cholesterol, in the adult brain they rely on delivery of cholesterol from neighbouring astrocytes via ApoE‐containing lipoproteins [59, 60], but can activate the neuronal cholesterol biosynthesis pathway in response to oxidative stress *in vitro* [61]. In support of this observation, we demonstrate the significant up‐regulation of cholesterol biosynthesis genes in neurones with high levels of a DDR.

In summary, we have defined the molecular signature of a neuronal DDR, which associates with cognitive impairment in older individuals with only early stage Alzheimer‐type pathology. As these individuals did not have established Alzheimer's neuropathology, changes in the neuronal transcriptome are not attributable to established AD, and may be independent of Alzheimer's or interact with the earliest molecular stages of the disease, or may reflect brain ageing. No other significant brain pathologies were present. We demonstrate an association between a persistent neuronal DDR, increased cholesterol biosynthesis, impaired insulin/IGF and Wnt signalling, and increased GSK3β, which may contribute to neuronal dysfunction and cognitive impairment. As these mechanisms, operating at the earliest stages of Alzheimer's neuropathology, are potential therapeutic targets it is important to understand their role in cognitive decline and to develop biomarkers to identify individuals who may benefit from targeting such pathways at preclinical disease stages.

## Author contributions

S. B. W., P. G. I., P. J. S., P. R. H., C. B. and M. R. conceived and designed the experiments. J. E. S., L. R. and C. G. performed the experiments. S. B. W., F. E. M., T. M., J. E. S., E. G. and P. R. H. analysed the data. J. E. S. and S. B. W. wrote the paper.

## Disclosure

All authors have seen and approved the paper. There are no conflicts of interest specific to this paper.

## Supporting information


**Table S1.** Up‐regulated genes in high versus low neuronal DDR cases at low Braak and Braak stages (*P* < 0.001).

**Table S2.** Down‐regulated genes in high versus low neuronal DDR cases at low Braak and Braak stages (*P* < 0.001).

## References

[1] CFANS . Pathological correlates of late‐onset dementia in a multicentre, community‐based population in England and Wales. Neuropathology Group of the Medical Research Council Cognitive Function and Ageing Study (MRC CFAS). Lancet 2001; 357: 169–175 11213093 10.1016/s0140-6736(00)03589-3

[2] Nunomura A , Perry G , Pappolla MA , Wade R , Hirai K , Chiba S , Smith MA . RNA oxidation is a prominent feature of vulnerable neurons in Alzheimer's disease. J Neurosci 1999; 19: 1959–1964 10066249 10.1523/JNEUROSCI.19-06-01959.1999 PMC6782583

[3] Nunomura A , Tamaoki T , Motohashi N , Nakamura M , McKeel DW Jr , Tabaton M , Lee HG , Smith MA , Perry G , Zhu X . The earliest stage of cognitive impairment in transition from normal aging to Alzheimer disease is marked by prominent RNA oxidation in vulnerable neurons. J Neuropathol Exp Neurol 2012; 71: 233–241 22318126 10.1097/NEN.0b013e318248e614 PMC3288284

[4] Wang J , Markesbery WR , Lovell MA . Increased oxidative damage in nuclear and mitochondrial DNA in mild cognitive impairment. J Neurochem 2006; 96: 825–832 16405502 10.1111/j.1471-4159.2005.03615.x

[5] Shrivastav M , De Haro LP , Nickoloff JA . Regulation of DNA double‐strand break repair pathway choice. Cell Res 2008; 18: 134–147 18157161 10.1038/cr.2007.111

[6] Simpson JE , Ince PG , Haynes LJ , Theaker R , Gelsthorpe C , Baxter L , Forster G , Lace GL , Shaw PJ , Matthews FE , Savva GM , Brayne C , Wharton SB . Population variation in oxidative stress and astrocyte DNA damage in relation to Alzheimer‐type pathology in the ageing brain. Neuropathol Appl Neurobiol 2010; 36: 25–40 19422529 10.1111/j.1365-2990.2009.01030.x

[7] Braak H , Braak E . Neuropathological stageing of Alzheimer‐related changes. Acta Neuropathol 1991; 82: 239–259 1759558 10.1007/BF00308809

[8] Simpson JE , Ince PG , Matthews FE , Shaw PJ , Heath PR , Brayne C , Garwood C , Higginbottom A , Wharton SB . A neuronal DNA damage response is detected at the earliest stages of Alzheimer's neuropathology and correlates with cognitive impairment in the MRC‐CFAS ageing brain cohort. Neuropathol Appl Neurobiol 2015; 41: 483–496. doi: 10.1111/nan.12202; in press 25443110 10.1111/nan.12202 PMC4861215

[9] Talbot K , Wang HY , Kazi H , Han LY , Bakshi KP , Stucky A , Fuino RL , Kawaguchi KR , Samoyedny AJ , Wilson RS , Arvanitakis Z , Schneider JA , Wolf BA , Bennett DA , Trojanowski JQ , Arnold SE . Demonstrated brain insulin resistance in Alzheimer's disease patients is associated with IGF‐1 resistance, IRS‐1 dysregulation, and cognitive decline. J Clin Invest 2012; 122: 1316–1338 22476197 10.1172/JCI59903 PMC3314463

[10] Wharton SB , Brayne C , Savva GM , Matthews FE , Forster G , Simpson J , Lace G , Ince PG . Epidemiological neuropathology: the MRC Cognitive Function and Aging Study experience. J Alzheimers Dis 2011; 25: 359–372 21422529 10.3233/JAD-2011-091402

[11] Medway C , Morgan K . Review: the genetics of Alzheimer's disease; putting flesh on the bones. Neuropathol Appl Neurobiol 2014; 40: 97–105 24443964 10.1111/nan.12101 PMC4282344

[12] Stephan BCM , Wharton SB , Simpson JE , Matthews F , Ince P , Brayne C . The epidemiological neuropathology of dementia and the implications for drug development. Neurodegen Dis Manage 2012; 2: 1–12

[13] Brayne C , Barker R , Grupe A , Harold D , Ince P , Savva G , Williams J , Williams‐Gray C , Wharton S . From molecule to clinic and community for neurodegeneration: research to bridge translational gaps. J Alzheimers Dis 2012; 33: S385–396 10.3233/JAD-2012-129006 22647264

[14] Counts SE , Alldred MJ , Che S , Ginsberg SD , Mufson EJ . Synaptic gene dysregulation within hippocampal CA1 pyramidal neurons in mild cognitive impairment. Neuropharmacology 2014; 79: 172–179 24445080 10.1016/j.neuropharm.2013.10.018 PMC3951099

[15] Forabosco P , Ramasamy A , Trabzuni D , Walker R , Smith C , Bras J , Levine AP , Hardy J , Pocock JM , Guerreiro R , Weale ME , Ryten M . Insights into TREM2 biology by network analysis of human brain gene expression data. Neurobiol Aging 2013; 34: 2699–2714 23855984 10.1016/j.neurobiolaging.2013.05.001 PMC3988951

[16] Blalock EM , Geddes JW , Chen KC , Porter NM , Markesbery WR , Landfield PW . Incipient Alzheimer's disease: microarray correlation analyses reveal major transcriptional and tumor suppressor responses. Proc Natl Acad Sci U S A 2004; 101: 2173–2178 14769913 10.1073/pnas.0308512100 PMC357071

[17] Lu T , Pan Y , Kao SY , Li C , Kohane I , Chan J , Yankner BA . Gene regulation and DNA damage in the ageing human brain. Nature 2004; 429: 883–891 15190254 10.1038/nature02661

[18] Silva AR , Grinberg LT , Farfel JM , Diniz BS , Lima LA , Silva PJ , Ferretti RE , Rocha RM , Filho WJ , Carraro DM , Brentani H . Transcriptional alterations related to neuropathology and clinical manifestation of Alzheimer's disease. PLoS ONE 2012; 7: e48751 23144955 10.1371/journal.pone.0048751 PMC3492444

[19] Ince PG , McArthur FK , Bjertness E , Torvik A , Candy JM , Edwardson JA . Neuropathological diagnoses in elderly patients in Oslo: Alzheimer's disease, Lewy body disease, vascular lesions. Dementia 1995; 6: 162–168 7620529 10.1159/000106940

[20] Mirra SS . The CERAD neuropathology protocol and consensus recommendations for the postmortem diagnosis of Alzheimer's disease: a commentary. Neurobiol Aging 1997; 18: S91–94 9330994 10.1016/s0197-4580(97)00058-4

[21] Savva GM , Wharton SB , Ince PG , Forster G , Matthews FE , Brayne C . Age, neuropathology, and dementia. N Engl J Med 2009; 360: 2302–2309 19474427 10.1056/NEJMoa0806142

[22] CFANS . Cognitive function and dementia in six areas of England and Wales: the distribution of MMSE and prevalence of GMS organicity level in the Medical Research Council Cognitive Function and Ageing Study (MRC CFAS). Psychol Med 1998; 28: 319–335 9572090 10.1017/s0033291797006272

[23] Nicoll JA , Savva GM , Stewart J , Matthews FE , Brayne C , Ince P . Association between APOE genotype, neuropathology and dementia in the older population of England and Wales. Neuropathol Appl Neurobiol 2011; 37: 285–294 20880354 10.1111/j.1365-2990.2010.01130.x

[24] Wu Z , Irizarry RA . Preprocessing of oligonucleotide array data. Nat Biotechnol 2004; 22: 656–658 10.1038/nbt0604-656b 15175677

[25] da Huang W , Sherman BT , Lempicki RA . Systematic and integrative analysis of large gene lists using DAVID bioinformatics resources. Nat Protoc 2009; 4: 44–57 19131956 10.1038/nprot.2008.211

[26] Pfrieger FW . Role of cholesterol in synapse formation and function. Biochim Biophys Acta 2003; 1610: 271–280 12648780 10.1016/s0005-2736(03)00024-5

[27] Serrano‐Pozo A , Qian J , Monsell SE , Blacker D , Gomez‐Isla T , Betensky RA , Growdon JH , Johnson KA , Frosch MP , Sperling RA , Hyman BT . Mild to moderate Alzheimer dementia with insufficient neuropathological changes. Ann Neurol 2014; 75: 597–601 24585367 10.1002/ana.24125 PMC4016558

[28] Craft S . Insulin resistance and Alzheimer's disease pathogenesis: potential mechanisms and implications for treatment. Curr Alzheimer Res 2007; 4: 147–152 17430239 10.2174/156720507780362137

[29] Neumann KF , Rojo L , Navarrete LP , Farias G , Reyes P , Maccioni RB . Insulin resistance and Alzheimer's disease: molecular links & clinical implications. Curr Alzheimer Res 2008; 5: 438–447 18855585 10.2174/156720508785908919

[30] Schubert M , Gautam D , Surjo D , Ueki K , Baudler S , Schubert D , Kondo T , Alber J , Galldiks N , Kustermann E , Arndt S , Jacobs AH , Krone W , Kahn CR , Bruning JC . Role for neuronal insulin resistance in neurodegenerative diseases. Proc Natl Acad Sci U S A 2004; 101: 3100–3105 14981233 10.1073/pnas.0308724101 PMC365750

[31] Hoyer S . Glucose metabolism and insulin receptor signal transduction in Alzheimer disease. Eur J Pharmacol 2004; 490: 115–125 15094078 10.1016/j.ejphar.2004.02.049

[32] Steen E , Terry BM , Rivera EJ , Cannon JL , Neely TR , Tavares R , Xu XJ , Wands JR , de la Monte SM . Impaired insulin and insulin‐like growth factor expression and signaling mechanisms in Alzheimer's disease – is this type 3 diabetes? J Alzheimers Dis 2005; 7: 63–80 15750215 10.3233/jad-2005-7107

[33] Frolich L , Blum‐Degen D , Bernstein HG , Engelsberger S , Humrich J , Laufer S , Muschner D , Thalheimer A , Turk A , Hoyer S , Zochling R , Boissl KW , Jellinger K , Riederer P . Brain insulin and insulin receptors in aging and sporadic Alzheimer's disease. J Neural Transm 1998; 105: 423–438 9720972 10.1007/s007020050068

[34] Hoyer S . The aging brain. Changes in the neuronal insulin/insulin receptor signal transduction cascade trigger late‐onset sporadic Alzheimer disease (SAD). A mini‐review. J Neural Transm 2002; 109: 991–1002 12111436 10.1007/s007020200082

[35] Moloney AM , Griffin RJ , Timmons S , O'Connor R , Ravid R , O'Neill C . Defects in IGF‐1 receptor, insulin receptor and IRS‐1/2 in Alzheimer's disease indicate possible resistance to IGF‐1 and insulin signalling. Neurobiol Aging 2010; 31: 224–243 18479783 10.1016/j.neurobiolaging.2008.04.002

[36] Craft S , Asthana S , Newcomer JW , Wilkinson CW , Matos IT , Baker LD , Cherrier M , Lofgreen C , Latendresse S , Petrova A , Plymate S , Raskind M , Grimwood K , Veith RC . Enhancement of memory in Alzheimer disease with insulin and somatostatin, but not glucose. Arch Gen Psychiatry 1999; 56: 1135–1140 10591291 10.1001/archpsyc.56.12.1135

[37] De Ferrari GV , Inestrosa NC . Wnt signaling function in Alzheimer's disease. Brain Res Brain Res Rev 2000; 33: 1–12 10967351 10.1016/s0165-0173(00)00021-7

[38] Alvarez AR , Godoy JA , Mullendorff K , Olivares GH , Bronfman M , Inestrosa NC . Wnt‐3a overcomes beta‐amyloid toxicity in rat hippocampal neurons. Exp Cell Res 2004; 297: 186–196 15194435 10.1016/j.yexcr.2004.02.028

[39] Patel S , Doble B , Woodgett JR . Glycogen synthase kinase‐3 in insulin and Wnt signalling: a double‐edged sword? Biochem Soc Trans 2004; 32: 803–808 15494020 10.1042/BST0320803 PMC4485494

[40] Hooper C , Killick R , Lovestone S . The GSK3 hypothesis of Alzheimer's disease. J Neurochem 2008; 104: 1433–1439 18088381 10.1111/j.1471-4159.2007.05194.x PMC3073119

[41] de la Monte SM . Brain insulin resistance and deficiency as therapeutic targets in Alzheimer's disease. Curr Alzheimer Res 2012; 9: 35–66 22329651 10.2174/156720512799015037 PMC3349985

[42] Smith MA , Rudnicka‐Nawrot M , Richey PL , Praprotnik D , Mulvihill P , Miller CA , Sayre LM , Perry G . Carbonyl‐related posttranslational modification of neurofilament protein in the neurofibrillary pathology of Alzheimer's disease. J Neurochem 1995; 64: 2660–2666 7539057 10.1046/j.1471-4159.1995.64062660.x

[43] Liu Q , Xie F , Siedlak SL , Nunomura A , Honda K , Moreira PI , Zhua X , Smith MA , Perry G . Neurofilament proteins in neurodegenerative diseases. Cell Mol Life Sci 2004; 61: 3057–3075 15583867 10.1007/s00018-004-4268-8 PMC11924432

[44] Zhu X , Su B , Wang X , Smith MA , Perry G . Causes of oxidative stress in Alzheimer disease. Cell Mol Life Sci 2007; 64: 2202–2210 17605000 10.1007/s00018-007-7218-4 PMC11136009

[45] Gamba P , Leonarduzzi G , Tamagno E , Guglielmotto M , Testa G , Sottero B , Gargiulo S , Biasi F , Mauro A , Vina J , Poli G . Interaction between 24‐hydroxycholesterol, oxidative stress, and amyloid‐beta in amplifying neuronal damage in Alzheimer's disease: three partners in crime. Aging Cell 2011; 10: 403–417 21272192 10.1111/j.1474-9726.2011.00681.x

[46] Lutjohann D , Breuer O , Ahlborg G , Nennesmo I , Siden A , Diczfalusy U , Bjorkhem I . Cholesterol homeostasis in human brain: evidence for an age‐dependent flux of 24S‐hydroxycholesterol from the brain into the circulation. Proc Natl Acad Sci U S A 1996; 93: 9799–9804 8790411 10.1073/pnas.93.18.9799 PMC38509

[47] Lutjohann D , von Bergmann K . 24S‐hydroxycholesterol: a marker of brain cholesterol metabolism. Pharmacopsychiatry 2003; 36: S102–106 14574622 10.1055/s-2003-43053

[48] Papassotiropoulos A , Lutjohann D , Bagli M , Locatelli S , Jessen F , Buschfort R , Ptok U , Bjorkhem I , von Bergmann K , Heun R . 24S‐hydroxycholesterol in cerebrospinal fluid is elevated in early stages of dementia. J Psychiatr Res 2002; 36: 27–32 11755458 10.1016/s0022-3956(01)00050-4

[49] Leoni V , Shafaati M , Salomon A , Kivipelto M , Bjorkhem I , Wahlund LO . Are the CSF levels of 24S‐hydroxycholesterol a sensitive biomarker for mild cognitive impairment? Neurosci Lett 2006; 397: 83–87 16406316 10.1016/j.neulet.2005.11.046

[50] Leoni V , Solomon A , Lovgren‐Sandblom A , Minthon L , Blennow K , Hansson O , Wahlund LO , Kivipelto M , Bjorkhem I . Diagnostic power of 24S‐hydroxycholesterol in cerebrospinal fluid: candidate marker of brain health. J Alzheimers Dis 2013; 36: 739–747 23666171 10.3233/JAD-130035

[51] Thiele C , Hannah MJ , Fahrenholz F , Huttner WB . Cholesterol binds to synaptophysin and is required for biogenesis of synaptic vesicles. Nat Cell Biol 2000; 2: 42–49 10620806 10.1038/71366

[52] Glockner F , Ohm TG . Tau pathology induces intraneuronal cholesterol accumulation. J Neuropathol Exp Neurol 2014; 73: 846–854 25101701 10.1097/NEN.0000000000000103

[53] Koudinov AR , Koudinova NV . Cholesterol homeostasis failure as a unifying cause of synaptic degeneration. J Neurol Sci 2005; 229–230: 233–240 10.1016/j.jns.2004.11.036 15760645

[54] Wellington CL . Cholesterol at the crossroads: Alzheimer's disease and lipid metabolism. Clin Genet 2004; 66: 1–16 15200500 10.1111/j.0009-9163.2004.00280.x

[55] Hartmann T . Cholesterol, A beta and Alzheimer's disease. Trends Neurosci 2001; 24: S45–48 11881745 10.1016/s0166-2236(00)01990-1

[56] Oikawa N , Hatsuta H , Murayama S , Suzuki A , Yanagisawa K . Influence of APOE genotype and the presence of Alzheimer's pathology on synaptic membrane lipids of human brains. J Neurosci Res 2014; 92: 641–650 24446209 10.1002/jnr.23341

[57] Ali‐Rahmani F , Schengrund CL , Connor JR . HFE gene variants, iron, and lipids: a novel connection in Alzheimer's disease. Front Pharmacol 2014; 5: 165 25071582 10.3389/fphar.2014.00165 PMC4086322

[58] Goldstein JL , DeBose‐Boyd RA , Brown MS . Protein sensors for membrane sterols. Cell 2006; 124: 35–46 16413480 10.1016/j.cell.2005.12.022

[59] Mauch DH , Nagler K , Schumacher S , Goritz C , Muller EC , Otto A , Pfrieger FW . CNS synaptogenesis promoted by glia‐derived cholesterol. Science 2001; 294: 1354–1357 11701931 10.1126/science.294.5545.1354

[60] Dietschy JM , Turley SD . Thematic review series: brain lipids. Cholesterol metabolism in the central nervous system during early development and in the mature animal. J Lipid Res 2004; 45: 1375–1397 15254070 10.1194/jlr.R400004-JLR200

[61] Recuero M , Vicente MC , Martinez‐Garcia A , Ramos MC , Carmona‐Saez P , Sastre I , Aldudo J , Vilella E , Frank A , Bullido MJ , Valdivieso F . A free radical‐generating system induces the cholesterol biosynthesis pathway: a role in Alzheimer's disease. Aging Cell 2009; 8: 128–139 19239419 10.1111/j.1474-9726.2009.00457.x

