# Supplementary material for: Neuronal DNA damage response‐associated dysregulation of signalling pathways and cholesterol metabolism at the earliest stages of Alzheimer‐type pathology
Source: Neuropathol Appl Neurobiol. 2015 Jul 7;42(2):167–79. doi: 10.1111/nan.12252 (PMC5102584; doi:10.1111/nan.12252)
Supplement: Supplementary file 1 — Table S1. Up‐regulated genes in high versus low neuronal DDR cases at low Braak and Braak stages (P < 0.001). [file NAN-42-167-s001.docx]

**Supplementary Table 1.** Up-regulated genes in high versus low neuronal DDR cases at low Braak and Braak stages (p<0.001)

| **Probe set ID** | **Gene** | **Symbol** | ***p-value*** | **FC** |
| --- | --- | --- | --- | --- |
| *DNA damage response* | |  |  |  |

| 212211_at | ankyrin repeat domain 17 | ANKRD17 | 0.00023 | 2.67 |
| --- | --- | --- | --- | --- |
| 204093_at | cyclin H | CCNH | 0.00098 | 3.76 |
| 211297_s_at | cyclin-dependent kinase 7 | CDK7 | 0.00031 | 2.54 |
| 214583_at | DNA-damage inducible 1 homolog 2 (S. cerevisiae) /// regulatory solute carrier protein, family 1, member 1 | DDI2 /// RSC1A1 | 0.00021 | 2.41 |
| 202971_s_at | dual-specificity tyrosine-(Y)-phosphorylation regulated kinase 2 | DYRK2 | 0.00022 | 2.65 |
| 224162_s_at | F-box protein 31 | FBXO31 | 0.00098 | 2.13 |
| 239068_at | guanine nucleotide binding protein-like 1 | GNL1 | 0.00079 | 2.12 |
| 226352_at | junction mediating and regulatory protein, p53 cofactor | JMY | 0.00002 | 2.06 |
| 224561_s_at | mortality factor 4 like 1 | MORF4L1 | 0.00086 | 2.66 |
| 201865_x_at | nuclear receptor subfamily 3, group C, member 1 (glucocorticoid receptor) | NR3C1 | 0.00099 | 2.14 |
| 226507_at | p21 protein (Cdc42/Rac)-activated kinase 1 | PAK1 | 0.00015 | 4.33 |
| 214086_s_at | poly (ADP-ribose) polymerase 2 | PARP2 | 0.00082 | 3.39 |
| 201202_at | proliferating cell nuclear antigen | PCNA | 0.00020 | 2.19 |
| 221206_at | PMS2 postmeiotic segregation increased 2 (S. cerevisiae) /// PMS2 C-terminal like pseudogene | PMS2 /// PMS2CL | 0.00097 | 1.74 |
| 203616_at | polymerase (DNA directed), beta | POLB | 0.00053 | 2.48 |
| 238992_at | polymerase (DNA directed) iota | POLI | 0.00088 | 3.39 |
| 201409_s_at | protein phosphatase 1, catalytic subunit, beta isozyme | PPP1CB | 0.00091 | 2.24 |
| 207405_s_at | RAD17 homolog (S. pombe) | RAD17 | 0.00070 | 2.71 |
| 201223_s_at | RAD23 homolog B (S. cerevisiae) | RAD23B | 0.00052 | 3.33 |
| 204146_at | RAD51 associated protein 1 | RAD51AP1 | 0.00041 | 3.02 |
| 218598_at | RAD50 interactor 1 | RINT1 | 0.00014 | 4.87 |
| 203160_s_at | ring finger protein 8, E3 ubiquitin protein ligase | RNF8 | 0.00052 | 2.05 |
| 209259_s_at | structural maintenance of chromosomes 3 | SMC3 | 0.00011 | 2.50 |
| 202266_at | tyrosyl-DNA phosphodiesterase 2 | TDP2 | 0.00030 | 3.64 |
| 203611_at | telomeric repeat binding factor 2 | TERF2 | 0.00020 | 1.67 |
| 202606_s_at | tousled-like kinase 1 | TLK1 | 0.00017 | 3.37 |
| 228627_at | tousled-like kinase 2 | TLK2 | 0.00053 | 2.04 |
| 204033_at | thyroid hormone receptor interactor 13 | TRIP13 | 0.00003 | 1.77 |
| 226557_at | tetratricopeptide repeat domain 5 | TTC5 | 0.00012 | 1.49 |
| 201898_s_at | ubiquitin-conjugating enzyme E2A | UBE2A | 0.00039 | 2.41 |
| 202334_s_at | ubiquitin-conjugating enzyme E2B | UBE2B | 0.00036 | 2.08 |
| 201524_x_at | ubiquitin-conjugating enzyme E2N | UBE2N | 0.00008 | 1.65 |
| 228734_at | ubiquitin-conjugating enzyme E2 variant 2 | UBE2V2 | 0.00020 | 7.41 |
| 220746_s_at | ubiquitin interaction motif containing 1 | UIMC1 | 0.00018 | 1.69 |
| 226015_at | zinc finger protein 12 | ZNF12 | 0.00023 | 5.86 |

|  |  |  |  |  |
| --- | --- | --- | --- | --- |
| *Cellular response to stress* | |  |  |  |
| 205047_s_at | asparagine synthetase (glutamine-hydrolyzing) | ASNS | 0.00002 | 4.72 |
| 201855_s_at | ATM interactor | ATMIN | 0.00024 | 4.24 |
| 225144_at | bone morphogenetic protein receptor, type II (serine/threonine kinase) | BMPR2 | 0.00076 | 5.11 |
| 217814_at | coiled-coil domain containing 47 | CCDC47 | 0.00085 | 2.10 |
| 212180_at | v-crk sarcoma virus CT10 oncogene homolog (avian)-like | CRKL | 0.00020 | 2.39 |
| 222543_at | derlin 1 | DERL1 | 0.00094 | 2.65 |
| 201144_s_at | eukaryotic translation initiation factor 2, subunit 1 alpha, 35kDa | EIF2S1 | 0.00097 | 1.69 |
| 219933_at | glutaredoxin 2 | GLRX2 | 0.00039 | 2.85 |
| 226191_at | glycogen synthase kinase 3 beta | GSK3B | 0.00004 | 4.19 |
| 209566_at | insulin induced gene 2 | INSIG2 | 0.00034 | 4.38 |
| 205841_at | Janus kinase 2 | JAK2 | 0.00068 | 4.22 |
| 228565_at | mixed lineage kinase 4 | KIAA1804 | 0.00025 | 4.12 |
| 212233_at | microtubule-associated protein 1B | MAP1B | 0.00085 | 4.28 |
| 226084_at | microtubule-associated protein 1B | MAP1B | 0.00039 | 2.14 |
| 203038_at | protein tyrosine phosphatase, receptor type, K | PTPRK | 0.00009 | 2.06 |
| 204466_s_at | synuclein, alpha (non A4 component of amyloid precursor) | SNCA | 0.00005 | 9.91 |
| 204467_s_at | synuclein, alpha (non A4 component of amyloid precursor) | SNCA | 0.00015 | 4.48 |
| 224705_s_at | trinucleotide repeat containing 6A | TNRC6A | 0.00052 | 2.36 |
| 234734_s_at | trinucleotide repeat containing 6A | TNRC6A | 0.00014 | 1.70 |
| 225923_at | VAMP (vesicle-associated membrane protein)-associated protein B and C | VAPB | 0.00011 | 2.72 |
|  |  |  |  |  |
| *Synapse* |  |  |  |  |
| 213101_s_at | ARP3 actin-related protein 3 homolog (yeast) | ACTR3 | 0.00006 | 2.52 |
| 213102_at | ARP3 actin-related protein 3 homolog (yeast) | ACTR3 | 0.00004 | 2.03 |
| 1557820_at | AFG3 ATPase family member 3-like 2 (S. cerevisiae) | AFG3L2 | 0.00004 | 1.90 |
| 210962_s_at | A kinase (PRKA) anchor protein (yotiao) 9 | AKAP9 | 0.00046 | 1.90 |
| 238786_at | ankyrin 3, node of Ranvier (ankyrin G) | ANK3 | 0.00033 | 3.61 |
| 209871_s_at | amyloid beta (A4) precursor protein-binding, family A, member 2 | APBA2 | 0.00023 | 1.83 |
| 204685_s_at | ATPase, Ca++ transporting, plasma membrane 2 | ATP2B2 | 0.00056 | 2.75 |
| 216120_s_at | ATPase, Ca++ transporting, plasma membrane 2 | ATP2B2 | 0.00049 | 3.90 |
| 214762_at | ATPase, H+ transporting, lysosomal 13kDa, V1 subunit G2 | ATP6V1G2 | 0.00017 | 8.24 |
| 204586_at | bassoon presynaptic cytomatrix protein | BSN | 0.00034 | 3.14 |
| 208320_at | calcium binding protein 1 | CABP1 | 0.00002 | 2.15 |
| 242410_s_at | calcium channel, voltage-dependent, R type, alpha 1E subunit | CACNA1E | 0.00006 | 4.49 |
| 213714_at | calcium channel, voltage-dependent, beta 2 subunit | CACNB2 | 0.00014 | 6.16 |
| 1568604_a_at | Ca++-dependent secretion activator | CADPS | 0.00020 | 6.44 |
| 204814_at | Ca++-dependent secretion activator | CADPS | 0.00032 | 4.00 |
| 219572_at | Ca++-dependent secretion activator 2 | CADPS2 | 0.00004 | 3.78 |
| 242524_at | cerebellin 4 precursor | CBLN4 | 0.00070 | 11.71 |
| 214596_at | cholinergic receptor, muscarinic 3 | CHRM3 | 0.00021 | 2.21 |
| 201561_s_at | calsyntenin 1 | CLSTN1 | 0.00100 | 1.90 |
| 223500_at | complexin 1 | CPLX1 | 0.00057 | 3.33 |
| 210227_at | discs, large (Drosophila) homolog-associated protein 2 | DLGAP2 | 0.00001 | 3.04 |
| 203881_s_at | dystrophin | DMD | 0.00017 | 2.24 |
| 213938_at | ELKS/RAB6-interacting/CAST family member 2 | ERC2 | 0.00022 | 4.38 |
| 203619_s_at | Fas apoptotic inhibitory molecule 2 | FAIM2 | 0.00000 | 2.52 |
| 209471_s_at | farnesyltransferase, CAAX box, alpha | FNTA | 0.00087 | 1.73 |
| 209990_s_at | gamma-aminobutyric acid (GABA) B receptor, 2 | GABBR2 | 0.00020 | 3.61 |
| 211679_x_at | gamma-aminobutyric acid (GABA) B receptor, 2 | GABBR2 | 0.00064 | 1.67 |
| 217077_s_at | gamma-aminobutyric acid (GABA) B receptor, 2 | GABBR2 | 0.00086 | 1.48 |
| 244118_at | gamma-aminobutyric acid (GABA) A receptor, alpha 1 | GABRA1 | 0.00055 | 7.58 |
| 227690_at | gamma-aminobutyric acid (GABA) A receptor, beta 3 | GABRB3 | 0.00004 | 11.15 |
| 227830_at | gamma-aminobutyric acid (GABA) A receptor, beta 3 | GABRB3 | 0.00003 | 3.88 |
| 1568612_at | gamma-aminobutyric acid (GABA) A receptor, gamma 2 | GABRG2 | 0.00082 | 7.85 |
| 244680_at | glycine receptor, beta | GLRB | 0.00004 | 6.95 |
| 205280_at | glycine receptor, beta | GLRB | 0.00016 | 10.96 |
| 225022_at | golgi-associated PDZ and coiled-coil motif containing | GOPC | 0.00040 | 2.42 |
| 227846_at | G protein-coupled receptor 176 | GPR176 | 0.00019 | 2.14 |
| 209793_at | glutamate receptor, ionotropic, AMPA 1 | GRIA1 | 0.00007 | 2.64 |
| 236538_at | glutamate receptor, ionotropic, AMPA 2 | GRIA2 | 0.00003 | 2.84 |
| 230144_at | glutamate receptor, ionotropic, AMPA 3 | GRIA3 | 0.00003 | 2.78 |
| 214611_at | glutamate receptor, ionotropic, kainate 1 | GRIK1 | 0.00089 | 2.18 |
| 231384_at | glutamate receptor, ionotropic, N-methyl D-aspartate 2A | GRIN2A | 0.00062 | 4.78 |
| 207299_s_at | glutamate receptor, metabotropic 1 | GRM1 | 0.00007 | 8.08 |
| 214217_at | glutamate receptor, metabotropic 5 | GRM5 | 0.00016 | 2.48 |
| 207548_at | glutamate receptor, metabotropic 7 /// uncharacterized LOC100996542 | GRM7 /// LOC100996542 | 0.00015 | 2.49 |
| 226651_at | homer homolog 1 (Drosophila) | HOMER1 | 0.00093 | 2.10 |
| 230849_at | potassium voltage-gated channel, shaker-related subfamily, member 1 (episodic ataxia with myokymia) | KCNA1 | 0.00002 | 5.35 |
| 207103_at | potassium voltage-gated channel, Shal-related subfamily, member 2 | KCND2 | 0.00009 | 6.38 |
| 228414_at | potassium large conductance calcium-activated channel, subfamily M, alpha member 1 | KCNMA1 | 0.00037 | 2.04 |
| 228579_at | potassium voltage-gated channel, KQT-like subfamily, member 3 | KCNQ3 | 0.00051 | 2.90 |
| 244623_at | potassium voltage-gated channel, KQT-like subfamily, member 5 | KCNQ5 | 0.00048 | 15.07 |
| 209234_at | kinesin family member 1B | KIF1B | 0.00042 | 2.97 |
| 1556047_s_at | melanoma antigen family E, 1 | MAGEE1 | 0.00018 | 6.65 |
| 229286_at | melanoma antigen family E, 1 | MAGEE1 | 0.00005 | 3.40 |
| 202228_s_at | neuroplastin | NPTN | 0.00016 | 6.15 |
| 242865_at | neuroplastin | NPTN | 0.00034 | 2.69 |
| 204684_at | neuronal pentraxin I | NPTX1 | 0.00031 | 2.68 |
| 205440_s_at | neuropeptide Y receptor Y1 | NPY1R | 0.00087 | 2.82 |
| 204105_s_at | neuronal cell adhesion molecule | NRCAM | 0.00056 | 2.29 |
| 209915_s_at | neurexin 1 | NRXN1 | 0.00007 | 6.11 |
| 228547_at | neurexin 1 | NRXN1 | 0.00095 | 5.73 |
| 229649_at | neurexin 3 | NRXN3 | 0.00034 | 7.00 |
| 205795_at | neurexin 3 | NRXN3 | 0.00076 | 3.71 |
| 231726_at | protocadherin beta 14 | PCDHB14 | 0.00035 | 4.34 |
| 207081_s_at | phosphatidylinositol 4-kinase, catalytic, alpha | PI4KA | 0.00007 | 3.86 |
| 213408_s_at | phosphatidylinositol 4-kinase, catalytic, alpha /// phosphatidylinositol 4-kinase, catalytic, alpha pseudogene 1 /// phosphatidylinositol 4-kinase, catalytic, alpha pseudogene 2 | PI4KA /// PI4KAP1 /// PI4KAP2 | 0.00016 | 1.96 |
| 201133_s_at | praja ring finger 2, E3 ubiquitin protein ligase | PJA2 | 0.00022 | 2.69 |
| 228494_at | protein phosphatase 1, regulatory subunit 9A | PPP1R9A | 0.00025 | 3.08 |
| 233985_x_at | protein phosphatase 1, regulatory subunit 9A | PPP1R9A | 0.00048 | 1.45 |
| 202429_s_at | protein phosphatase 3, catalytic subunit, alpha isozyme | PPP3CA | 0.00029 | 9.20 |
| 202432_at | protein phosphatase 3, catalytic subunit, beta isozyme | PPP3CB | 0.00073 | 4.25 |
| 218613_at | pleckstrin and Sec7 domain containing 3 | PSD3 | 0.00049 | 6.01 |
| 214811_at | RIMS binding protein 2 | RIMBP2 | 0.00024 | 4.68 |
| 231986_at | regulating synaptic membrane exocytosis 1 | RIMS1 | 0.00003 | 2.90 |
| 204730_at | regulating synaptic membrane exocytosis 3 | RIMS3 | 0.00030 | 2.10 |
| 205230_at | rabphilin 3A homolog (mouse) | RPH3A | 0.00035 | 2.78 |
| 235225_at | sodium channel, voltage-gated, type II, beta subunit | SCN2B | 0.00041 | 5.57 |
| 214293_at | septin 11 | SEPT11 | 0.00051 | 2.91 |
| 210040_at | solute carrier family 12 (potassium/chloride transporter), member 5 | SLC12A5 | 0.00033 | 4.44 |
| 204229_at | solute carrier family 17 (sodium-dependent inorganic phosphate cotransporter), member 7 | SLC17A7 | 0.00004 | 3.23 |
| 1554593_s_at | solute carrier family 1 (high affinity aspartate/glutamate transporter), member 6 | SLC1A6 | 0.00079 | 8.59 |
| 204953_at | synaptosomal-associated protein, 91kDa | SNAP91 | 0.00011 | 8.70 |
| 226794_at | syntaxin binding protein 5 (tomosyn) | STXBP5 | 0.00091 | 4.36 |
| 232426_at | synaptic vesicle glycoprotein 2B | SV2B | 0.00017 | 4.55 |
| 205551_at | synaptic vesicle glycoprotein 2B | SV2B | 0.00012 | 5.22 |
| 229818_at | SV2 related protein homolog (rat) | SVOP | 0.00066 | 2.95 |
| 229039_at | synapsin II | SYN2 | 0.00015 | 8.25 |
| 232027_at | spectrin repeat containing, nuclear envelope 1 | SYNE1 | 0.00001 | 3.24 |
| 205691_at | synaptogyrin 3 | SYNGR3 | 0.00061 | 3.28 |
| 203998_s_at | synaptotagmin I | SYT1 | 0.00039 | 19.62 |
| 203999_at | synaptotagmin I | SYT1 | 0.00034 | 7.77 |
| 212701_at | talin 2 | TLN2 | 0.00028 | 2.60 |
| 226186_at | tropomodulin 2 (neuronal) | TMOD2 | 0.00052 | 2.27 |
| 209390_at | tuberous sclerosis 1 | TSC1 | 0.00025 | 1.35 |
| 201672_s_at | ubiquitin specific peptidase 14 (tRNA-guanine transglycosylase) | USP14 | 0.00046 | 3.49 |
| 208845_at | voltage-dependent anion channel 3 | VDAC3 | 0.00007 | 1.60 |
|  |  |  |  |  |
| *Wnt signalling* |  |  |  |  |
| 222696_at | axin 2 | AXIN2 | 0.00011 | 2.05 |
| 224471_s_at | beta-transducin repeat containing E3 ubiquitin protein ligase | BTRC | 0.00013 | 3.06 |
| 211761_s_at | calcyclin binding protein | CACYBP | 0.00046 | 3.10 |
| 209956_s_at | calcium/calmodulin-dependent protein kinase II beta | CAMK2B | 0.00002 | 4.48 |
| 211483_x_at | calcium/calmodulin-dependent protein kinase II beta | CAMK2B | 0.00043 | 2.07 |
| 210404_x_at | calcium/calmodulin-dependent protein kinase II beta | CAMK2B | 0.00033 | 2.16 |
| 225019_at | calcium/calmodulin-dependent protein kinase II delta | CAMK2D | 0.00052 | 2.88 |
| 227767_at | casein kinase 1, gamma 3 | CSNK1G3 | 0.00099 | 2.23 |
| 214247_s_at | dickkopf WNT signaling pathway inhibitor 3 | DKK3 | 0.00021 | 2.15 |
| 209455_at | F-box and WD repeat domain containing 11 | FBXW11 | 0.00033 | 2.71 |
| 219683_at | frizzled family receptor 3 | FZD3 | 0.00074 | 2.78 |
| 225068_at | kelch-like family member 12 | KLHL12 | 0.00098 | 4.16 |
| 218364_at | leucine rich repeat (in FLII) interacting protein 2 | LRRFIP2 | 0.00021 | 1.65 |
| 224984_at | nuclear factor of activated T-cells 5, tonicity-responsive | NFAT5 | 0.00007 | 2.97 |
| 222589_at | nemo-like kinase | NLK | 0.00067 | 3.26 |
| 203966_s_at | protein phosphatase, Mg2+/Mn2+ dependent, 1A | PPM1A | 0.00004 | 2.89 |
| 208652_at | protein phosphatase 2, catalytic subunit, alpha isozyme | PPP2CA | 0.00035 | 8.06 |
| 230708_at | prickle homolog 1 (Drosophila) | PRICKLE1 | 0.00085 | 2.95 |
| 226069_at | prickle homolog 1 (Drosophila) | PRICKLE1 | 0.00009 | 5.69 |
| 225968_at | prickle homolog 2 (Drosophila) | PRICKLE2 | 0.00095 | 3.38 |
| 209685_s_at | protein kinase C, beta | PRKCB | 0.00010 | 2.92 |
| 207957_s_at | protein kinase C, beta | PRKCB | 0.00083 | 10.95 |
| 202762_at | Rho-associated, coiled-coil containing protein kinase 2 | ROCK2 | 0.00028 | 3.59 |
| 200719_at | S-phase kinase-associated protein 1 | SKP1 | 0.00041 | 2.39 |
| 200711_s_at | S-phase kinase-associated protein 1 | SKP1 | 0.00031 | 2.56 |
| 203075_at | SMAD family member 2 | SMAD2 | 0.00057 | 2.34 |
| 229103_at | wingless-type MMTV integration site family, member 3 | WNT3 | 0.00085 | 2.66 |
|  |  |  |  |  |
| *MAPK signalling* |  |  |  |  |
| 213198_at | activin A receptor, type IB | ACVR1B | 0.00000 | 1.77 |
| 212607_at | v-akt murine thymoma viral oncogene homolog 3 (protein kinase B, gamma) | AKT3 | 0.00022 | 3.16 |
| 219714_s_at | calcium channel, voltage-dependent, alpha 2/delta subunit 3 | CACNA2D3 | 0.00005 | 6.12 |
| 214230_at | cell division cycle 42 | CDC42 | 0.00036 | 6.87 |
| 201536_at | dual specificity phosphatase 3 | DUSP3 | 0.00025 | 1.54 |
| 238521_at | fibroblast growth factor 12 | FGF12 | 0.00003 | 9.34 |
| 230231_at | fibroblast growth factor 14 | FGF14 | 0.00013 | 4.33 |
| 239178_at | fibroblast growth factor 9 (glia-activating factor) | FGF9 | 0.00018 | 4.60 |
| 223049_at | growth factor receptor-bound protein 2 | GRB2 | 0.00095 | 2.22 |
| 204009_s_at | v-Ki-ras2 Kirsten rat sarcoma viral oncogene homolog | KRAS | 0.00092 | 4.16 |
| 227562_at | late endosomal/lysosomal adaptor, MAPK and MTOR activator 3 | LAMTOR3 | 0.00027 | 4.21 |
| 202670_at | mitogen-activated protein kinase kinase 1 | MAP2K1 | 0.00073 | 5.79 |
| 203266_s_at | mitogen-activated protein kinase kinase 4 | MAP2K4 | 0.00048 | 3.29 |
| 229664_at | mitogen-activated protein kinase 8 | MAPK8 | 0.00025 | 3.10 |
| 203218_at | mitogen-activated protein kinase 9 | MAPK9 | 0.00007 | 5.94 |
| 225781_at | mitogen-activated protein kinase 9 | MAPK9 | 0.00011 | 3.69 |
| 209332_s_at | MYC associated factor X | MAX | 0.00005 | 2.12 |
| 218411_s_at | MAP3K12 binding inhibitory protein 1 | MBIP | 0.00070 | 2.65 |
| 209199_s_at | myocyte enhancer factor 2C | MEF2C | 0.00028 | 5.39 |
| 210675_s_at | protein tyrosine phosphatase, receptor type, R | PTPRR | 0.00032 | 3.06 |
| 203097_s_at | Rap guanine nucleotide exchange factor (GEF) 2 | RAPGEF2 | 0.00000 | 5.41 |
| 224769_at | TAO kinase 1 | TAOK1 | 0.00099 | 2.03 |
|  |  |  |  |  |
| *Calcium signalling* |  |  |  |  |
| 213245_at | adenylate cyclase 1 (brain) | ADCY1 | 0.00047 | 3.35 |
| 212361_s_at | ATPase, Ca++ transporting, cardiac muscle, slow twitch 2 | ATP2A2 | 0.00016 | 5.92 |
| 242036_x_at | ATPase, Ca++ transporting, plasma membrane 3 | ATP2B3 | 0.00053 | 3.95 |
| 200623_s_at | calmodulin 1 (phosphorylase kinase, delta) /// calmodulin 2 (phosphorylase kinase, delta) /// calmodulin 3 (phosphorylase kinase, delta) | CALM1 /// CALM2 /// CALM3 | 0.00010 | 1.95 |
| 226382_at | calcium/calmodulin-dependent protein kinase ID /// uncharacterized LOC283070 | CAMK1D /// LOC283070 | 0.00035 | 3.70 |
| 213268_at | calmodulin binding transcription activator 1 | CAMTA1 | 0.00033 | 6.88 |
| 214157_at | GNAS complex locus | GNAS | 0.00051 | 3.86 |
| 217673_x_at | GNAS complex locus | GNAS | 0.00064 | 1.83 |
| 236234_at | phosphodiesterase 1A, calmodulin-dependent | PDE1A | 0.00012 | 7.94 |
| 235518_at | solute carrier family 8 (sodium/calcium exchanger), member 1 | SLC8A1 | 0.00055 | 3.53 |
| 205803_s_at | transient receptor potential cation channel, subfamily C, member 1 | TRPC1 | 0.00075 | 2.82 |
|  |  |  |  |  |
| *Phosphatidylinositol signalling* | |  |  |  |
| 238694_at | diacylglycerol kinase, epsilon 64kDa | DGKE | 0.00028 | 3.11 |
| 235695_at | inositol polyphosphate-4-phosphatase, type I, 107kDa | INPP4A | 0.00057 | 2.59 |
| 203446_s_at | oculocerebrorenal syndrome of Lowe | OCRL | 0.00068 | 3.45 |
| 212240_s_at | phosphoinositide-3-kinase, regulatory subunit 1 (alpha) | PIK3R1 | 0.00062 | 1.89 |
| 218942_at | phosphatidylinositol-5-phosphate 4-kinase, type II, gamma | PIP4K2C | 0.00019 | 2.23 |
| 212990_at | synaptojanin 1 | SYNJ1 | 0.00012 | 4.92 |
|  |  |  |  |  |
| *Neurotrophin signalling* | |  |  |  |
| 204686_at | insulin receptor substrate 1 | IRS1 | 0.00074 | 2.41 |
| 208743_s_at | tyrosine 3-monooxygenase/tryptophan 5-monooxygenase activation protein, beta polypeptide | YWHAB | 0.00002 | 9.77 |
| 217718_s_at | tyrosine 3-monooxygenase/tryptophan 5-monooxygenase activation protein, beta polypeptide | YWHAB | 0.00047 | 2.02 |
|  |  |  |  |  |
| *Secretion* |  |  |  |  |
| 218098_at | ADP-ribosylation factor guanine nucleotide-exchange factor 2 (brefeldin A-inhibited) | ARFGEF2 | 0.00002 | 5.70 |
| 222127_s_at | exocyst complex component 1 | EXOC1 | 0.00086 | 2.86 |
| 206857_s_at | FK506 binding protein 1B, 12.6 kDa | FKBP1B | 0.00035 | 4.20 |
| 208693_s_at | glycyl-tRNA synthetase | GARS | 0.00031 | 2.75 |
| 221467_at | melanocortin 4 receptor | MC4R | 0.00024 | 1.69 |
| 212310_at | melanoma inhibitory activity family, member 3 | MIA3 | 0.00065 | 3.26 |
| 212448_at | neural precursor cell expressed, developmentally down-regulated 4-like, E3 ubiquitin protein ligase | NEDD4L | 0.00021 | 1.80 |
| 222824_at | nudix (nucleoside diphosphate linked moiety X)-type motif 5 | NUDT5 | 0.00007 | 1.78 |
| 236838_at | SRC kinase signaling inhibitor 1 | SRCIN1 | 0.00013 | 1.45 |
| 202260_s_at | syntaxin binding protein 1 | STXBP1 | 0.00035 | 3.22 |
| 215518_at | syntaxin binding protein 5-like | STXBP5L | 0.00091 | 3.03 |
| 219736_at | tripartite motif containing 36 | TRIM36 | 0.00034 | 11.70 |
| 225926_at | vesicle transport through interaction with t-SNAREs 1B | VTI1B | 0.00024 | 2.30 |
|  |  |  |  |  |
| *Cytoskeleton* |  |  |  |  |
| 225098_at | abl-interactor 2 | ABI2 | 0.00058 | 1.80 |
| 226893_at | v-abl Abelson murine leukemia viral oncogene homolog 2 | ABL2 | 0.00041 | 2.13 |
| 203861_s_at | actinin, alpha 2 | ACTN2 | 0.00029 | 1.92 |
| 203863_at | actinin, alpha 2 | ACTN2 | 0.00028 | 2.76 |
| 218395_at | ARP6 actin-related protein 6 homolog (yeast) | ACTR6 | 0.00041 | 5.47 |
| 203156_at | A kinase (PRKA) anchor protein 11 | AKAP11 | 0.00016 | 6.43 |
| 202630_at | amyloid beta precursor protein (cytoplasmic tail) binding protein 2 | APPBP2 | 0.00082 | 2.36 |
| 226914_at | actin related protein 2/3 complex, subunit 5-like | ARPC5L | 0.00003 | 5.98 |
| 202391_at | brain abundant, membrane attached signal protein 1 | BASP1 | 0.00011 | 3.59 |
| 210817_s_at | calcium binding and coiled-coil domain 2 | CALCOCO2 | 0.00075 | 1.32 |
| 212710_at | calmodulin regulated spectrin-associated protein 1 | CAMSAP1 | 0.00049 | 3.44 |
| 212711_at | calmodulin regulated spectrin-associated protein 1 | CAMSAP1 | 0.00043 | 2.77 |
| 212765_at | calmodulin regulated spectrin-associated protein family, member 2 | CAMSAP2 | 0.00001 | 3.44 |
| 225010_at | coiled-coil domain containing 6 | CCDC6 | 0.00006 | 4.46 |
| 205899_at | cyclin A1 | CCNA1 | 0.00013 | 3.61 |
| 202717_s_at | cell division cycle 16 | CDC16 | 0.00089 | 2.43 |
| 217880_at | cell division cycle 27 | CDC27 | 0.00053 | 2.25 |
| 226449_at | centrosomal protein 120kDa | CEP120 | 0.00058 | 2.01 |
| 207719_x_at | centrosomal protein 170kDa | CEP170 | 0.00078 | 1.84 |
| 212746_s_at | centrosomal protein 170kDa | CEP170 | 0.00088 | 2.48 |
| 52285_f_at | centrosomal protein 76kDa | CEP76 | 0.00010 | 5.32 |
| 209194_at | centrin, EF-hand protein, 2 | CETN2 | 0.00040 | 2.82 |
| 212832_s_at | cytoskeleton associated protein 5 | CKAP5 | 0.00086 | 2.43 |
| 201975_at | CAP-GLY domain containing linker protein 1 | CLIP1 | 0.00018 | 2.65 |
| 229116_at | connector enhancer of kinase suppressor of Ras 2 | CNKSR2 | 0.00016 | 4.49 |
| 206731_at | connector enhancer of kinase suppressor of Ras 2 | CNKSR2 | 0.00027 | 6.37 |
| 221676_s_at | coronin, actin binding protein, 1C | CORO1C | 0.00026 | 2.26 |
| 209789_at | coronin, actin binding protein, 2B | CORO2B | 0.00045 | 2.41 |
| 1556877_at | catenin, alpha 3 | CTNNA3 | 0.00037 | 2.99 |
| 213295_at | cylindromatosis (turban tumor syndrome) | CYLD | 0.00066 | 3.29 |
| 203139_at | death-associated protein kinase 1 | DAPK1 | 0.00091 | 3.65 |
| 215116_s_at | dynamin 1 | DNM1 | 0.00013 | 2.74 |
| 203105_s_at | dynamin 1-like | DNM1L | 0.00001 | 2.51 |
| 230933_at | destrin (actin depolymerizing factor) | DSTN | 0.00092 | 4.62 |
| 205348_s_at | dynein, cytoplasmic 1, intermediate chain 1 | DYNC1I1 | 0.00070 | 5.72 |
| 211684_s_at | dynein, cytoplasmic 1, intermediate chain 2 | DYNC1I2 | 0.00024 | 1.92 |
| 217976_s_at | dynein, cytoplasmic 1, light intermediate chain 1 | DYNC1LI1 | 0.00039 | 6.65 |
| 203303_at | dynein, light chain, Tctex-type 3 | DYNLT3 | 0.00021 | 2.62 |
| 231930_at | ELMO/CED-12 domain containing 1 | ELMOD1 | 0.00015 | 5.78 |
| 204796_at | echinoderm microtubule associated protein like 1 | EML1 | 0.00007 | 4.00 |
| 242443_at | echinoderm microtubule associated protein like 5 | EML5 | 0.00062 | 2.26 |
| 201340_s_at | ectodermal-neural cortex 1 (with BTB domain) | ENC1 | 0.00019 | 8.06 |
| 201341_at | ectodermal-neural cortex 1 (with BTB domain) | ENC1 | 0.00007 | 8.76 |
| 212339_at | erythrocyte membrane protein band 4.1-like 1 | EPB41L1 | 0.00010 | 3.25 |
| 212681_at | erythrocyte membrane protein band 4.1-like 3 | EPB41L3 | 0.00049 | 4.87 |
| 218875_s_at | F-box protein 5 | FBXO5 | 0.00025 | 3.60 |
| 218980_at | formin homology 2 domain containing 3 | FHOD3 | 0.00032 | 4.12 |
| 210142_x_at | flotillin 1 | FLOT1 | 0.00098 | 1.90 |
| 225168_at | FERM domain containing 4A | FRMD4A | 0.00013 | 2.05 |
| 215052_at | FERM and PDZ domain containing 4 | FRMPD4 | 0.00075 | 2.60 |
| 228744_at | HAUS augmin-like complex, subunit 2 | HAUS2 | 0.00062 | 3.44 |
| 225792_at | hook homolog 1 (Drosophila) | HOOK1 | 0.00034 | 5.79 |
| 226395_at | hook homolog 3 (Drosophila) | HOOK3 | 0.00029 | 2.80 |
| 36030_at | intermediate filament family orphan 1 | IFFO1 | 0.00028 | 1.89 |
| 218100_s_at | intraflagellar transport 57 homolog (Chlamydomonas) | IFT57 | 0.00001 | 3.16 |
| 204465_s_at | internexin neuronal intermediate filament protein, alpha | INA | 0.00048 | 8.14 |
| 238600_at | janus kinase and microtubule interacting protein 1 | JAKMIP1 | 0.00030 | 2.31 |
| 227713_at | katanin p60 subunit A-like 1 | KATNAL1 | 0.00057 | 2.25 |
| 212427_at | KIAA0368 | KIAA0368 | 0.00042 | 2.40 |
| 203086_at | kinesin heavy chain member 2A | KIF2A | 0.00028 | 2.54 |
| 228680_at | kinesin family member 3A | KIF3A | 0.00069 | 3.45 |
| 203389_at | kinesin family member 3C | KIF3C | 0.00080 | 2.45 |
| 226534_at | KIT ligand | KITLG | 0.00030 | 4.80 |
| 214591_at | kelch-like family member 4 | KLHL4 | 0.00060 | 2.77 |
| 243998_at | keratin 222 | KRT222 | 0.00002 | 5.02 |
| 244111_at | keratin 222 | KRT222 | 0.00004 | 5.40 |
| 222561_at | LanC lantibiotic synthetase component C-like 2 (bacterial) | LANCL2 | 0.00003 | 2.75 |
| 214099_s_at | myomegalin-like | LOC100996724 | 0.00018 | 2.52 |
| 219338_s_at | leucine rich repeat containing 49 | LRRC49 | 0.00016 | 3.46 |
| 223492_s_at | leucine rich repeat (in FLII) interacting protein 1 | LRRFIP1 | 0.00035 | 2.28 |
| 203151_at | microtubule-associated protein 1A | MAP1A | 0.00049 | 2.14 |
| 225540_at | microtubule-associated protein 2 | MAP2 | 0.00062 | 2.01 |
| 235066_at | microtubule-associated protein 4 | MAP4 | 0.00027 | 9.84 |
| 228448_at | microtubule-associated protein 6 | MAP6 | 0.00023 | 1.91 |
| 228943_at | microtubule-associated protein 6 | MAP6 | 0.00028 | 1.90 |
| 220145_at | microtubule-associated protein 9 | MAP9 | 0.00054 | 2.53 |
| 221047_s_at | MAP/microtubule affinity-regulating kinase 1 | MARK1 | 0.00007 | 2.69 |
| 208017_s_at | MCF.2 cell line derived transforming sequence | MCF2 | 0.00092 | 6.13 |
| 212473_s_at | microtubule associated monooxygenase, calponin and LIM domain containing 2 | MICAL2 | 0.00074 | 2.02 |
| 1556903_at | midline | MID1 | 0.00072 | 2.00 |
| 223012_at | microRNA 4746 /// UBX domain protein 6 | MIR4746 /// UBXN6 | 0.00060 | 1.76 |
| 227761_at | myosin VA (heavy chain 12, myoxin) | MYO5A | 0.00002 | 3.75 |
| 208093_s_at | nudE nuclear distribution E homolog (A. nidulans)-like 1 | NDEL1 | 0.00005 | 3.09 |
| 33767_at | neurofilament, heavy polypeptide | NFH | 0.00055 | 8.31 |
| 221801_x_at | neurofilament, light polypeptide | NFL | 0.00011 | 13.87 |
| 221805_at | neurofilament, light polypeptide | NFL | 0.00017 | 36.03 |
| 221916_at | neurofilament, light polypeptide | NFL | 0.00012 | 5.89 |
| 223442_at | nicolin 1 | NICN1 | 0.00075 | 2.57 |
| 201577_at | NME/NM23 nucleoside diphosphate kinase 1 | NME1 | 0.00015 | 3.83 |
| 224830_at | nudix (nucleoside diphosphate linked moiety X)-type motif 21 | NUDT21 | 0.00038 | 5.33 |
| 228962_at | phosphodiesterase 4D, cAMP-specific | PDE4D | 0.00089 | 4.81 |
| 63305_at | PBX/knotted 1 homeobox 2 | PKNOX2 | 0.00007 | 2.68 |
| 1555900_at | polo-like kinase 1 | PLK1 | 0.00034 | 3.60 |
| 203407_at | periplakin | PPL | 0.00066 | 3.15 |
| 215172_at | protein tyrosine phosphatase, non-receptor type 20A /// protein tyrosine phosphatase, non-receptor type 20B | PTPN20A /// PTPN20B | 0.00000 | 10.50 |
| 236935_at | protein tyrosine phosphatase, non-receptor type 4 (megakaryocyte) | PTPN4 | 0.00001 | 5.02 |
| 202582_s_at | RAN binding protein 9 | RANBP9 | 0.00028 | 2.45 |
| 244739_at | radixin | RDX | 0.00029 | 3.91 |
| 1552789_at | SEC62 homolog (S. cerevisiae) | SEC62 | 0.00080 | 3.69 |
| 1552790_a_at | SEC62 homolog (S. cerevisiae) | SEC62 | 0.00086 | 5.08 |
| 213666_at | septin 6 | SEPT6 | 0.00090 | 3.23 |
| 226627_at | septin 8 | SEPT8 | 0.00026 | 2.32 |
| 222010_at | small nucleolar RNA, H/ACA box 29 /// t-complex 1 | SNORA29 /// TCP1 | 0.00033 | 2.85 |
| 210033_s_at | sperm associated antigen 6 | SPAG6 | 0.00081 | 2.29 |
| 229417_at | staufen double-stranded RNA binding protein 2 | STAU2 | 0.00011 | 3.26 |
| 204226_at | staufen double-stranded RNA binding protein 2 | STAU2 | 0.00026 | 3.52 |
| 202786_at | serine threonine kinase 39 | STK39 | 0.00084 | 2.87 |
| 200783_s_at | stathmin 1 | STMN1 | 0.00068 | 2.98 |
| 223245_at | spermatid perinuclear RNA binding protein | STRBP | 0.00001 | 6.33 |
| 223246_s_at | spermatid perinuclear RNA binding protein | STRBP | 0.00046 | 2.90 |
| 212828_at | synaptojanin 2 | SYNJ2 | 0.00001 | 2.33 |
| 202349_at | torsin family 1, member A (torsin A) | TOR1A | 0.00037 | 2.03 |
| 230104_s_at | tubulin polymerization promoting protein | TPPP | 0.00099 | 1.42 |
| 203846_at | tripartite motif containing 32 | TRIM32 | 0.00044 | 1.47 |
| 226964_at | tau tubulin kinase 2 | TTBK2 | 0.00007 | 3.90 |
| 226120_at | tetratricopeptide repeat domain 8 | TTC8 | 0.00072 | 3.75 |
| 209026_x_at | tubulin, beta class I | TUBB | 0.00075 | 2.74 |
| 203894_at | tubulin, gamma 2 | TUBG2 | 0.00081 | 1.75 |
| 208780_x_at | VAMP (vesicle-associated membrane protein)-associated protein A, 33kDa | VAPA | 0.00022 | 1.40 |
| 224813_at | Wiskott-Aldrich syndrome-like | WASL | 0.00037 | 3.98 |
|  |  |  |  |  |
| *Proteolysis* |  |  |  |  |
| 206046_at | ADAM metallopeptidase domain 23 | ADAM23 | 0.00081 | 1.95 |
| 209001_s_at | anaphase promoting complex subunit 13 | ANAPC13 | 0.00048 | 2.49 |
| 225521_at | anaphase promoting complex subunit 7 | ANAPC7 | 0.00018 | 3.42 |
| 209974_s_at | BUB3 mitotic checkpoint protein | BUB3 | 0.00017 | 2.40 |
| 235224_s_at | cullin-associated and neddylation-dissociated 1 | CAND1 | 0.00075 | 3.32 |
| 200661_at | cathepsin A | CTSA | 0.00086 | 1.59 |
| 201372_s_at | cullin 3 | CUL3 | 0.00001 | 4.79 |
| 228546_at | dipeptidyl-peptidase 6 | DPP6 | 0.00098 | 2.85 |
| 207789_s_at | dipeptidyl-peptidase 6 | DPP6 | 0.00003 | 2.19 |
| 213186_at | DAZ interacting zinc finger protein 3 | DZIP3 | 0.00077 | 3.34 |
| 224628_at | endoplasmic reticulum lectin 1 | ERLEC1 | 0.00080 | 2.56 |
| 205774_at | coagulation factor XII (Hageman factor) | F12 | 0.00072 | 2.17 |
| 235850_at | family with sequence similarity 162, member A | FAM162A | 0.00069 | 2.96 |
| 227641_at | F-box and leucine-rich repeat protein 16 | FBXL16 | 0.00021 | 1.85 |
| 222119_s_at | F-box protein 11 | FBXO11 | 0.00018 | 2.73 |
| 202271_at | F-box protein 28 | FBXO28 | 0.00009 | 3.25 |
| 202272_s_at | F-box protein 28 | FBXO28 | 0.00034 | 2.20 |
| 1555971_s_at | F-box protein 28 | FBXO28 | 0.00029 | 2.33 |
| 218432_at | F-box protein 3 | FBXO3 | 0.00030 | 7.71 |
| 227521_at | F-box protein 33 | FBXO33 | 0.00036 | 2.47 |
| 218539_at | F-box protein 34 | FBXO34 | 0.00017 | 2.63 |
| 225100_at | F-box protein 45 | FBXO45 | 0.00010 | 2.15 |
| 242294_at | F-box protein 45 | FBXO45 | 0.00029 | 4.11 |
| 212987_at | F-box protein 9 | FBXO9 | 0.00039 | 2.59 |
| 235195_at | F-box and WD repeat domain containing 2 | FBXW2 | 0.00005 | 3.65 |
| 229419_at | F-box and WD repeat domain containing 7, E3 ubiquitin protein ligase | FBXW7 | 0.00060 | 3.09 |
| 213341_at | fem-1 homolog c (C. elegans) | FEM1C | 0.00052 | 2.47 |
| 243743_at | HECT, C2 and WW domain containing E3 ubiquitin protein ligase 1 | HECW1 | 0.00097 | 1.61 |
| 218306_s_at | HECT and RLD domain containing E3 ubiquitin protein ligase family member 1 | HERC1 | 0.00043 | 2.39 |
| 219352_at | HECT and RLD domain containing E3 ubiquitin protein ligase family member 6 | HERC6 | 0.00052 | 2.85 |
| 208598_s_at | HECT, UBA and WWE domain containing 1, E3 ubiquitin protein ligase | HUWE1 | 0.00007 | 1.84 |
| 222471_s_at | potassium channel modulatory factor 1 | KCMF1 | 0.00017 | 4.68 |
| 226215_s_at | lysine (K)-specific demethylase 2B | KDM2B | 0.00068 | 1.75 |
| 201212_at | legumain | LGMN | 0.00047 | 3.61 |
| 223612_s_at | ligand of numb-protein X 1, E3 ubiquitin protein ligase | LNX1 | 0.00016 | 2.90 |
| 229663_at | lon peptidase 2, peroxisomal | LONP2 | 0.00077 | 1.63 |
| 225996_at | LON peptidase N-terminal domain and ring finger 2 | LONRF2 | 0.00054 | 6.59 |
| 239359_at | membrane-associated ring finger (C3HC4) 11 | MAR11 | 0.00046 | 3.09 |
| 201455_s_at | aminopeptidase puromycin sensitive | NPEPPS | 0.00087 | 2.49 |
| 219557_s_at | nuclear receptor interacting protein 3 | NRIP3 | 0.00014 | 3.84 |
| 202395_at | N-ethylmaleimide-sensitive factor | NSF | 0.00015 | 9.16 |
| 205825_at | proprotein convertase subtilisin/kexin type 1 | PCSK1 | 0.00011 | 6.77 |
| 204869_at | proprotein convertase subtilisin/kexin type 2 | PCSK2 | 0.00009 | 5.77 |
| 225274_at | prenylcysteine oxidase 1 | PCYOX1 | 0.00001 | 3.76 |
| 227639_at | phosphatidylinositol glycan anchor biosynthesis, class K | PIGK | 0.00019 | 2.69 |
| 218667_at | praja ring finger 1, E3 ubiquitin protein ligase | PJA1 | 0.00073 | 2.89 |
| 210759_s_at | proteasome (prosome, macropain) subunit, alpha type, 1 | PSMA1 | 0.00077 | 2.39 |
| 201316_at | proteasome (prosome, macropain) subunit, alpha type, 2 | PSMA2 | 0.00059 | 3.17 |
| 201274_at | proteasome (prosome, macropain) subunit, alpha type, 5 | PSMA5 | 0.00067 | 1.76 |
| 214288_s_at | proteasome (prosome, macropain) subunit, beta type, 1 | PSMB1 | 0.00060 | 1.49 |
| 201068_s_at | proteasome (prosome, macropain) 26S subunit, ATPase, 2 | PSMC2 | 0.00085 | 2.01 |
| 201198_s_at | proteasome (prosome, macropain) 26S subunit, non-ATPase, 1 | PSMD1 | 0.00047 | 3.23 |
| 208776_at | proteasome (prosome, macropain) 26S subunit, non-ATPase, 11 | PSMD11 | 0.00003 | 4.04 |
| 202353_s_at | proteasome (prosome, macropain) 26S subunit, non-ATPase, 12 | PSMD12 | 0.00018 | 5.80 |
| 212296_at | proteasome (prosome, macropain) 26S subunit, non-ATPase, 14 | PSMD14 | 0.00024 | 2.72 |
| 203447_at | proteasome (prosome, macropain) 26S subunit, non-ATPase, 5 | PSMD5 | 0.00086 | 2.49 |
| 201705_at | proteasome (prosome, macropain) 26S subunit, non-ATPase, 7 | PSMD7 | 0.00037 | 1.89 |
| 205174_s_at | glutaminyl-peptide cyclotransferase | QPCT | 0.00048 | 6.66 |
| 226922_at | RAN binding protein 2 | RANBP2 | 0.00067 | 3.28 |
| 234950_s_at | ring finger and WD repeat domain 2, E3 ubiquitin protein ligase | RFWD2 | 0.00011 | 1.86 |
| 225416_at | ring finger protein, LIM domain interacting | RLIM | 0.00074 | 2.76 |
| 208924_at | ring finger protein 11 | RNF11 | 0.00050 | 3.05 |
| 219263_at | ring finger protein 128, E3 ubiquitin protein ligase | RNF128 | 0.00044 | 6.11 |
| 201824_at | ring finger protein 14 | RNF14 | 0.00040 | 3.84 |
| 201962_s_at | ring finger protein 41 | RNF41 | 0.00089 | 4.21 |
| 203403_s_at | ring finger protein (C3H2C3 type) 6 | RNF6 | 0.00067 | 6.07 |
| 218286_s_at | ring finger protein 7 | RNF7 | 0.00028 | 2.32 |
| 203889_at | secretogranin V (7B2 protein) | SCG5 | 0.00021 | 6.27 |
| 201239_s_at | signal peptidase complex subunit 2 homolog (S. cerevisiae) | SPCS2 | 0.00066 | 1.80 |
| 227607_at | STAM binding protein-like 1 | STAMBPL1 | 0.00056 | 4.44 |
| 223330_s_at | SGT1, suppressor of G2 allele of SKP1 (S. cerevisiae) | SUGT1 | 0.00087 | 3.25 |
| 200740_s_at | small ubiquitin-like modifier 3 | SUMO3 | 0.00033 | 2.91 |
| 218289_s_at | ubiquitin-like modifier activating enzyme 5 | UBA5 | 0.00098 | 2.85 |
| 222579_at | ubiquitin-like modifier activating enzyme 5 | UBA5 | 0.00099 | 3.65 |
| 201343_at | ubiquitin-conjugating enzyme E2D 2 | UBE2D2 | 0.00040 | 2.48 |
| 201344_at | ubiquitin-conjugating enzyme E2D 2 | UBE2D2 | 0.00008 | 3.58 |
| 225651_at | ubiquitin-conjugating enzyme E2E 2 | UBE2E2 | 0.00011 | 2.81 |
| 225783_at | ubiquitin-conjugating enzyme E2F (putative) | UBE2F | 0.00006 | 2.18 |
| 209141_at | ubiquitin-conjugating enzyme E2G 1 | UBE2G1 | 0.00075 | 2.29 |
| 209142_s_at | ubiquitin-conjugating enzyme E2G 1 | UBE2G1 | 0.00024 | 4.13 |
| 225179_at | ubiquitin-conjugating enzyme E2K | UBE2K | 0.00037 | 3.94 |
| 217978_s_at | ubiquitin-conjugating enzyme E2Q family member 1 | UBE2Q1 | 0.00063 | 2.47 |
| 226612_at | ubiquitin-conjugating enzyme E2Q family-like 1 | UBE2QL1 | 0.00017 | 8.20 |
| 222657_s_at | ubiquitin-conjugating enzyme E2W (putative) | UBE2W | 0.00009 | 4.32 |
| 213291_s_at | ubiquitin protein ligase E3A | UBE3A | 0.00014 | 4.49 |
| 201817_at | ubiquitin protein ligase E3C | UBE3C | 0.00061 | 2.60 |
| 227790_at | ubiquitin protein ligase E3D | UBE3D | 0.00052 | 3.05 |
| 212760_at | ubiquitin protein ligase E3 component n-recognin 2 | UBR2 | 0.00018 | 3.03 |
| 230029_x_at | ubiquitin protein ligase E3 component n-recognin 3 (putative) | UBR3 | 0.00037 | 2.39 |
| 218108_at | ubiquitin protein ligase E3 component n-recognin 7 (putative) | UBR7 | 0.00076 | 3.21 |
| 201387_s_at | ubiquitin carboxyl-terminal esterase L1 (ubiquitin thiolesterase) | UCHL1 | 0.00036 | 5.90 |
| 219960_s_at | ubiquitin carboxyl-terminal hydrolase L5 | UCHL5 | 0.00011 | 4.05 |
| 220419_s_at | ubiquitin specific peptidase 25 | USP25 | 0.00008 | 2.65 |
| 227256_at | ubiquitin specific peptidase 31 | USP31 | 0.00079 | 5.91 |
| 226033_at | ubiquitin specific peptidase 31 | USP31 | 0.00004 | 2.67 |
| 226505_x_at | ubiquitin specific peptidase 32 | USP32 | 0.00070 | 2.56 |
| 213510_x_at | ubiquitin specific peptidase 32 pseudogene 2 | USP32P2 | 0.00030 | 3.19 |
| 212066_s_at | ubiquitin specific peptidase 34 | USP34 | 0.00012 | 1.91 |
| 238057_at | ubiquitin specific peptidase 45 | USP45 | 0.00078 | 4.29 |
| 225925_s_at | ubiquitin specific peptidase 48 | USP48 | 0.00086 | 1.69 |
| 229501_s_at | ubiquitin specific peptidase 8 | USP8 | 0.00097 | 1.53 |
| 229573_at | ubiquitin specific peptidase 9, X-linked | USP9X | 0.00073 | 2.01 |
| 1556228_a_at | valosin containing protein (p97)/p47 complex interacting protein 1 | VCPIP1 | 0.00016 | 4.75 |
| 212880_at | WD repeat domain 7 | WDR7 | 0.00002 | 6.03 |
| 201760_s_at | WD repeat and SOCS box containing 2 | WSB2 | 0.00017 | 3.05 |
| 201352_at | YME1-like 1 ATPase | YME1L1 | 0.00089 | 1.58 |
| 225350_s_at | zyg-11 family member B, cell cycle regulator | ZYG11B | 0.00005 | 2.93 |
|  |  |  |  |  |
| *Ion transport* |  |  |  |  |
| 209425_at | alpha-methylacyl-CoA racemase | AMACR | 0.00035 | 2.88 |
| 229176_at | ankylosis, progressive homolog (mouse) | ANKH | 0.00081 | 2.27 |
| 229313_at | anoctamin 5 | ANO5 | 0.00001 | 4.91 |
| 200761_s_at | ADP-ribosylation-like factor 6 interacting protein 5 | ARL6IP5 | 0.00026 | 2.26 |
| 201243_s_at | ATPase, Na+/K+ transporting, beta 1 polypeptide | ATP1B1 | 0.00020 | 6.75 |
| 212255_s_at | ATPase, Ca++ transporting, type 2C, member 1 | ATP2C1 | 0.00070 | 1.53 |
| 208870_x_at | ATP synthase, H+ transporting, mitochondrial F1 complex, gamma polypeptide 1 | ATP5C1 | 0.00045 | 3.51 |
| 213366_x_at | ATP synthase, H+ transporting, mitochondrial F1 complex, gamma polypeptide 1 | ATP5C1 | 0.00051 | 3.86 |
| 205711_x_at | ATP synthase, H+ transporting, mitochondrial F1 complex, gamma polypeptide 1 | ATP5C1 | 0.00020 | 3.45 |
| 208745_at | ATP synthase, H+ transporting, mitochondrial Fo complex, subunit G | ATP5L | 0.00015 | 2.40 |
| 200078_s_at | ATPase, H+ transporting, lysosomal 21kDa, V0 subunit b | ATP6V0B | 0.00048 | 1.82 |
| 201972_at | ATPase, H+ transporting, lysosomal 70kDa, V1 subunit A | ATP6V1A | 0.00014 | 4.71 |
| 201089_at | ATPase, H+ transporting, lysosomal 56/58kDa, V1 subunit B2 | ATP6V1B2 | 0.00038 | 3.45 |
| 202872_at | ATPase, H+ transporting, lysosomal 42kDa, V1 subunit C1 | ATP6V1C1 | 0.00049 | 4.35 |
| 226463_at | ATPase, H+ transporting, lysosomal 42kDa, V1 subunit C1 | ATP6V1C1 | 0.00000 | 7.12 |
| 208898_at | ATPase, H+ transporting, lysosomal 34kDa, V1 subunit D | ATP6V1D | 0.00028 | 3.81 |
| 214769_at | chloride channel, voltage-sensitive 4 | CLCN4 | 0.00005 | 5.44 |
| 203950_s_at | chloride channel, voltage-sensitive 6 | CLCN6 | 0.00007 | 1.87 |
| 217897_at | FXYD domain containing ion transport regulator 6 | FXYD6 | 0.00026 | 2.82 |
| 229294_at | junctophilin 3 | JPH3 | 0.00024 | 3.01 |
| 210454_s_at | potassium inwardly-rectifying channel, subfamily J, member 6 | KCNJ6 | 0.00043 | 3.32 |
| 244113_at | potassium inwardly-rectifying channel, subfamily J, member 9 | KCNJ9 | 0.00070 | 2.32 |
| 204678_s_at | potassium channel, subfamily K, member 1 | KCNK1 | 0.00061 | 2.03 |
| 220116_at | potassium intermediate/small conductance calcium-activated channel, subfamily N, member 2 | KCNN2 | 0.00023 | 6.93 |
| 220294_at | potassium channel, subfamily V, member 1 | KCNV1 | 0.00003 | 7.04 |
| 226246_at | potassium channel tetramerisation domain containing 1 | KCTD1 | 0.00002 | 2.66 |
| 233234_at | potassium channel tetramerisation domain containing 16 | KCTD16 | 0.00051 | 1.75 |
| 228608_at | sodium leak channel, non-selective | NALCN | 0.00004 | 2.72 |
| 202783_at | nicotinamide nucleotide transhydrogenase | NNT | 0.00062 | 2.35 |
| 227107_at | pannexin | PANX1 | 0.00008 | 4.26 |
| 210383_at | sodium channel, voltage-gated, type I, alpha subunit | SCN1A | 0.00016 | 5.89 |
| 229057_at | sodium channel, voltage-gated, type II, alpha subunit | SCN2A | 0.00002 | 6.69 |
| 210432_s_at | sodium channel, voltage-gated, type III, alpha subunit | SCN3A | 0.00014 | 3.82 |
| 204722_at | sodium channel, voltage-gated, type III, beta subunit | SCN3B | 0.00004 | 3.67 |
| 226741_at | solute carrier family 12 (potassium/chloride transporters), member 6 | SLC12A6 | 0.00020 | 1.39 |
| 223441_at | solute carrier family 17 (anion/sugar transporter), member 5 | SLC17A5 | 0.00010 | 3.09 |
| 203339_at | solute carrier family 25 (aspartate/glutamate carrier), member 12 | SLC25A12 | 0.00001 | 6.06 |
| 203340_s_at | solute carrier family 25 (aspartate/glutamate carrier), member 12 | SLC25A12 | 0.00001 | 4.11 |
| 235299_at | solute carrier family 41, member 2 | SLC41A2 | 0.00039 | 2.66 |
| 228792_at | solute carrier family 24, member 5 | SLC5 | 0.00052 | 4.21 |
| 203909_at | solute carrier family 9, subfamily A (NHE6, cation proton antiporter 6), member 6 | SLC9A6 | 0.00021 | 6.14 |
|  |  |  |  |  |
| *Transport* |  |  |  |  |
| 202852_s_at | alpha- and gamma-adaptin binding protein | AAGAB | 0.00007 | 2.29 |
| 222472_at | aftiphilin | AFTPH | 0.00051 | 3.16 |
| 205359_at | A kinase (PRKA) anchor protein 6 | AKAP6 | 0.00006 | 2.59 |
| 203299_s_at | adaptor-related protein complex 1, sigma 2 subunit | AP1S2 | 0.00021 | 2.25 |
| 212159_x_at | adaptor-related protein complex 2, alpha 2 subunit | AP2A2 | 0.00029 | 2.45 |
| 203410_at | adaptor-related protein complex 3, mu 2 subunit | AP3M2 | 0.00025 | 3.13 |
| 202442_at | adaptor-related protein complex 3, sigma 1 subunit | AP3S1 | 0.00002 | 2.14 |
| 200734_s_at | ADP-ribosylation factor 3 | ARF3 | 0.00009 | 3.67 |
| 218230_at | ADP-ribosylation factor interacting protein 1 | ARFIP1 | 0.00063 | 1.96 |
| 209824_s_at | aryl hydrocarbon receptor nuclear translocator-like | ARNTL | 0.00054 | 2.49 |
| 202372_at | aurora kinase A pseudogene 1 /// RAB3 GTPase activating protein subunit 2 (non-catalytic) | AURKAPS1 /// RAB3GAP2 | 0.00055 | 2.00 |
| 222437_s_at | charged multivesicular body protein 3 /// RNF103-CHMP3 readthrough | CHMP3 /// RNF103-CHMP3 | 0.00051 | 2.23 |
| 219356_s_at | charged multivesicular body protein 5 | CHMP5 | 0.00031 | 3.49 |
| 225769_at | component of oligomeric golgi complex 6 | COG6 | 0.00098 | 2.70 |
| 201359_at | coatomer protein complex, subunit beta 1 | COPB1 | 0.00087 | 3.50 |
| 213500_at | coatomer protein complex, subunit beta 2 | COPB2 | 0.00061 | 1.97 |
| 223420_at | DnaJ (Hsp40) homolog, subfamily C, member 14 | DNAJC14 | 0.00031 | 1.53 |
| 235296_at | eukaryotic translation initiation factor 5A2 | EIF5A2 | 0.00038 | 7.47 |
| 232067_at | failed axon connections homolog (Drosophila) | FAXC | 0.00003 | 4.81 |
| 230447_at | failed axon connections homolog (Drosophila) | FAXC | 0.00023 | 7.07 |
| 226145_s_at | Fraser syndrome 1 | FRAS1 | 0.00068 | 3.03 |
| 224887_at | N-acetylglucosamine-1-phosphate transferase, gamma subunit | GNPTG | 0.00005 | 1.49 |
| 222552_at | golgi transport 1B | GOLT1B | 0.00047 | 3.75 |
| 212434_at | GrpE-like 1, mitochondrial (E. coli) | GRPEL1 | 0.00035 | 2.27 |
| 54037_at | Hermansky-Pudlak syndrome 4 | HPS4 | 0.00095 | 1.41 |
| 202056_at | karyopherin alpha 1 (importin alpha 5) | KPNA1 | 0.00071 | 4.13 |
| 201088_at | karyopherin alpha 2 (RAG cohort 1, importin alpha 1) | KPNA2 | 0.00091 | 2.06 |
| 225268_at | karyopherin alpha 4 (importin alpha 3) | KPNA4 | 0.00020 | 2.14 |
| 226976_at | karyopherin alpha 6 (importin alpha 7) | KPNA6 | 0.00088 | 2.38 |
| 219643_at | low density lipoprotein receptor-related protein 1B | LRP1B | 0.00077 | 1.92 |
| 212245_at | multiple coagulation factor deficiency 2 | MCFD2 | 0.00074 | 3.54 |
| 203517_at | metaxin 2 | MTX2 | 0.00032 | 5.79 |
| 225111_s_at | N-ethylmaleimide-sensitive factor attachment protein, beta | NAPB | 0.00009 | 5.46 |
| 225448_at | N-ethylmaleimide-sensitive factor attachment protein, gamma | NAPG | 0.00034 | 5.67 |
| 209300_s_at | NECAP endocytosis associated 1 | NECAP1 | 0.00047 | 5.35 |
| 213682_at | nucleoporin 50kDa | NUP50 | 0.00043 | 4.86 |
| 202073_at | optineurin | OPTN | 0.00026 | 8.39 |
| 202074_s_at | optineurin | OPTN | 0.00070 | 2.45 |
| 229453_at | protein disulfide isomerase family A, member 3 | PDIA3 | 0.00090 | 2.30 |
| 203503_s_at | peroxisomal biogenesis factor 14 | PEX14 | 0.00029 | 1.39 |
| 203970_s_at | peroxisomal biogenesis factor 3 | PEX3 | 0.00081 | 2.23 |
| 239725_at | post-GPI attachment to proteins 1 | PGAP1 | 0.00046 | 2.77 |
| 213469_at | post-GPI attachment to proteins 1 | PGAP1 | 0.00032 | 2.23 |
| 213360_s_at | POM121 transmembrane nucleoporin /// POM121 transmembrane nucleoporin C | POM121 /// POM121C | 0.00035 | 1.90 |
| 200863_s_at | RAB11A, member RAS oncogene family | RAB11A | 0.00005 | 3.25 |
| 234998_at | RAB11A, member RAS oncogene family | RAB11A | 0.00049 | 5.74 |
| 225739_at | RAB11 family interacting protein 4 (class II) | RAB11FIP4 | 0.00064 | 2.09 |
| 59697_at | RAB15, member RAS oncogene family | RAB15 | 0.00000 | 2.10 |
| 221810_at | RAB15, member RAS oncogene family | RAB15 | 0.00016 | 1.99 |
| 224377_s_at | RAB18, member RAS oncogene family | RAB18 | 0.00002 | 3.78 |
| 213405_at | RAB22A, member RAS oncogene family | RAB22A | 0.00014 | 4.86 |
| 208731_at | RAB2A, member RAS oncogene family | RAB2A | 0.00050 | 3.46 |
| 208732_at | RAB2A, member RAS oncogene family | RAB2A | 0.00084 | 2.79 |
| 208733_at | RAB2A, member RAS oncogene family | RAB2A | 0.00076 | 4.91 |
| 203581_at | RAB4A, member RAS oncogene family /// S-phase response (cyclin related) | RAB4A /// SPHAR | 0.00077 | 1.87 |
| 221792_at | RAB6B, member RAS oncogene family | RAB6B | 0.00011 | 3.51 |
| 225259_at | RAB6B, member RAS oncogene family | RAB6B | 0.00051 | 4.52 |
| 211961_s_at | RAB7A, member RAS oncogene family | RAB7A | 0.00067 | 2.23 |
| 226633_at | RAB8B, member RAS oncogene family | RAB8B | 0.00088 | 2.24 |
| 200749_at | RAN, member RAS oncogene family | RAN | 0.00044 | 3.44 |
| 213019_at | RAN binding protein 6 | RANBP6 | 0.00047 | 2.77 |
| 228408_s_at | SDA1 domain containing 1 | SDAD1 | 0.00025 | 3.22 |
| 215696_s_at | SEC16 homolog A (S. cerevisiae) | SEC16A | 0.00021 | 2.47 |
| 228150_at | SEC16 homolog B (S. cerevisiae) | SEC16B | 0.00053 | 2.57 |
| 238078_at | SEC22 vesicle trafficking protein homolog A (S. cerevisiae) | SEC22A | 0.00007 | 4.81 |
| 212887_at | Sec23 homolog A (S. cerevisiae) | SEC23A | 0.00084 | 4.50 |
| 200945_s_at | SEC31 homolog A (S. cerevisiae) | SEC31A | 0.00039 | 2.69 |
| 218404_at | sorting nexin 10 | SNX10 | 0.00019 | 6.47 |
| 227031_at | sorting nexin 13 | SNX13 | 0.00098 | 3.35 |
| 213545_x_at | sorting nexin 3 | SNX3 | 0.00024 | 2.79 |
| 205335_s_at | signal recognition particle 19kDa | SRP19 | 0.00005 | 3.21 |
| 208801_at | signal recognition particle 72kDa | SRP72 | 0.00061 | 2.38 |
| 203544_s_at | signal transducing adaptor molecule (SH3 domain and ITAM motif) 1 | STAM | 0.00027 | 4.01 |
| 212799_at | syntaxin 6 | STX6 | 0.00002 | 2.82 |
| 212632_at | syntaxin 7 | STX7 | 0.00026 | 3.47 |
| 215171_s_at | translocase of inner mitochondrial membrane 17 homolog A (yeast) | TIMM17A | 0.00006 | 3.92 |
| 200662_s_at | translocase of outer mitochondrial membrane 20 homolog (yeast) | TOMM20 | 0.00090 | 6.52 |
| 225036_at | translocase of outer mitochondrial membrane 5 homolog (yeast) | TOMM5 | 0.00003 | 3.57 |
| 201519_at | translocase of outer mitochondrial membrane 70 homolog A (S. cerevisiae) | TOMM70A | 0.00002 | 2.89 |
| 218431_at | VPS33B interacting protein, apical-basolateral polarity regulator, spe-39 homolog | VIPAS39 | 0.00035 | 1.58 |
| 227987_at | vacuolar protein sorting 13 homolog A (S. cerevisiae) | VPS13A | 0.00002 | 5.20 |
| 227988_s_at | vacuolar protein sorting 13 homolog A (S. cerevisiae) | VPS13A | 0.00050 | 5.70 |
| 214785_at | vacuolar protein sorting 13 homolog A (S. cerevisiae) | VPS13A | 0.00073 | 2.59 |
| 201807_at | vacuolar protein sorting 26 homolog A (S. pombe) | VPS26A | 0.00013 | 2.26 |
| 222388_s_at | vacuolar protein sorting 35 homolog (S. cerevisiae) | VPS35 | 0.00010 | 5.27 |
| 225378_at | vacuolar protein sorting 37 homolog A (S. cerevisiae) | VPS37A | 0.00003 | 2.52 |
| 229653_at | vacuolar protein sorting 53 homolog (S. cerevisiae) | VPS53 | 0.00012 | 2.41 |
| 223022_s_at | Vps20-associated 1 homolog (S. cerevisiae) | VTA1 | 0.00045 | 2.35 |
| 212982_at | zinc finger, DHHC-type containing 17 | ZDHHC17 | 0.00044 | 2.39 |
|  |  |  |  |  |
| *Transcription* |  |  |  |  |
| 214956_at | AP2 associated kinase 1 | AAK1 | 0.00010 | 7.48 |
| 214998_at | AP2 associated kinase 1 | AAK1 | 0.00093 | 3.71 |
| 201128_s_at | ATP citrate lyase | ACLY | 0.00036 | 2.31 |
| 201661_s_at | acyl-CoA synthetase long-chain family member 3 | ACSL3 | 0.00069 | 2.73 |
| 201662_s_at | acyl-CoA synthetase long-chain family member 3 | ACSL3 | 0.00100 | 3.80 |
| 202422_s_at | acyl-CoA synthetase long-chain family member 4 | ACSL4 | 0.00019 | 5.54 |
| 206014_at | actin-like 6B | ACTL6B | 0.00005 | 2.96 |
| 205327_s_at | activin A receptor, type IIA | ACVR2A | 0.00004 | 2.39 |
| 228771_at | adrenergic, beta, receptor kinase 2 | ADRBK2 | 0.00020 | 3.28 |
| 221761_at | adenylosuccinate synthase | ADSS | 0.00069 | 3.07 |
| 218568_at | acylglycerol kinase | AGK | 0.00006 | 2.77 |
| 230652_at | v-raf murine sarcoma 3611 viral oncogene homolog | ARAF | 0.00021 | 3.22 |
| 235320_at | ADP-ribosylation factor-like 6 | ARL6 | 0.00060 | 4.21 |
| 227585_at | ATPase family, AAA domain containing 1 | ATAD1 | 0.00098 | 3.94 |
| 223340_at | atlastin GTPase 1 | ATL1 | 0.00055 | 2.77 |
| 219659_at | ATPase, aminophospholipid transporter, class I, type 8A, member 2 | ATP8A2 | 0.00024 | 2.47 |
| 231395_at | ATPase, aminophospholipid transporter, class I, type 8A, member 2 | ATP8A2 | 0.00014 | 2.62 |
| 215161_at | calcium/calmodulin-dependent protein kinase IG | CAMK1G | 0.00015 | 3.80 |
| 223460_at | calcium/calmodulin-dependent protein kinase kinase 1, alpha | CAMKK1 | 0.00022 | 2.04 |
| 208696_at | chaperonin containing TCP1, subunit 5 (epsilon) | CCT5 | 0.00005 | 2.37 |
| 204510_at | cell division cycle 7 | CDC7 | 0.00039 | 4.14 |
| 228991_at | cyclin-dependent kinase 13 | CDK13 | 0.00047 | 2.97 |
| 204831_at | cyclin-dependent kinase 8 | CDK8 | 0.00018 | 3.55 |
| 202157_s_at | CUGBP, Elav-like family member 2 | CELF2 | 0.00002 | 1.73 |
| 225031_at | chromodomain helicase DNA binding protein 6 | CHD6 | 0.00029 | 3.02 |
| 228751_at | CDC-like kinase 4 | CLK4 | 0.00073 | 1.65 |
| 222448_s_at | cytidine monophosphate (UMP-CMP) kinase 1, cytosolic | CMPK1 | 0.00019 | 2.96 |
| 205489_at | crystallin, mu | CRYM | 0.00070 | 3.71 |
| 202613_at | CTP synthase 1 | CTPS1 | 0.00004 | 2.19 |
| 203302_at | deoxycytidine kinase | DCK | 0.00048 | 3.97 |
| 205399_at | doublecortin-like kinase 1 | DCLK1 | 0.00047 | 5.22 |
| 215303_at | doublecortin-like kinase 1 | DCLK1 | 0.00080 | 5.25 |
| 201241_at | DEAD (Asp-Glu-Ala-Asp) box helicase 1 | DDX1 | 0.00071 | 4.51 |
| 204977_at | DEAD (Asp-Glu-Ala-Asp) box polypeptide 10 | DDX10 | 0.00005 | 3.66 |
| 219945_at | DEAD (Asp-Glu-Ala-Asp) box helicase 25 | DDX25 | 0.00051 | 2.30 |
| 201385_at | DEAH (Asp-Glu-Ala-His) box helicase 15 | DHX15 | 0.00056 | 1.79 |
| 229017_s_at | dual serine/threonine and tyrosine protein kinase | DSTYK | 0.00039 | 3.09 |
| 209033_s_at | dual-specificity tyrosine-(Y)-phosphorylation regulated kinase 1A | DYRK1A | 0.00004 | 2.69 |
| 212954_at | dual-specificity tyrosine-(Y)-phosphorylation regulated kinase 4 | DYRK4 | 0.00049 | 1.61 |
| 222398_s_at | elongation factor Tu GTP binding domain containing 2 | EFTUD2 | 0.00005 | 1.97 |
| 218935_at | EH-domain containing 3 | EHD3 | 0.00051 | 2.50 |
| 217736_s_at | eukaryotic translation initiation factor 2-alpha kinase 1 | EIF2AK1 | 0.00054 | 3.08 |
| 224936_at | eukaryotic translation initiation factor 2, subunit 3 gamma, 52kDa | EIF2S3 | 0.00075 | 1.84 |
| 200912_s_at | eukaryotic translation initiation factor 4A2 /// microRNA 1248 /// small nucleolar RNA, H/ACA box 4 /// small nucleolar RNA, H/ACA box 63 /// small nucleolar RNA, H/ACA box 81 /// small nucleolar RNA, C/D box 2 | EIF4A2 /// MIR1248 /// SNORA4 /// SNORA63 /// SNORA81 /// SNORD2 | 0.00062 | 2.31 |
| 203693_s_at | E2F transcription factor 3 | E2F3 | 0.00045 | 1.86 |
| 206051_at | ELAV (embryonic lethal, abnormal vision, Drosophila)-like 4 | ELAVL4 | 0.00018 | 2.40 |
| 238073_at | ELAV (embryonic lethal, abnormal vision, Drosophila)-like 4 | ELAVL4 | 0.00032 | 1.67 |
| 229374_at | EPH receptor A4 | EPHA4 | 0.00075 | 3.58 |
| 225290_at | ethanolamine kinase 1 | ETNK1 | 0.00048 | 6.35 |
| 219200_at | FAST kinase domains 3 | FASTKD3 | 0.00076 | 2.58 |
| 220415_at | FPGT-TNNI3K readthrough /// TNNI3 interacting kinase | FPGT-TNNI3K /// TNNI3K | 0.00019 | 2.35 |
| 210005_at | phosphoribosylglycinamide formyltransferase, phosphoribosylglycinamide synthetase, phosphoribosylaminoimidazole synthetase | GART | 0.00016 | 1.59 |
| 225392_at | G elongation factor, mitochondrial 2 | GFM2 | 0.00010 | 2.99 |
| 204993_at | guanine nucleotide binding protein (G protein), alpha z polypeptide | GNAZ | 0.00058 | 1.66 |
| 217850_at | guanine nucleotide binding protein-like 3 (nucleolar) /// small nucleolar RNA, C/D box 19B | GNL3 /// SNORD19B | 0.00028 | 2.03 |
| 218241_at | golgin A5 | GOLGA5 | 0.00001 | 3.22 |
| 212510_at | glycerol-3-phosphate dehydrogenase 1-like | GPD1L | 0.00061 | 3.13 |
| 203817_at | guanylate cyclase 1, soluble, beta 3 | GUCY1B3 | 0.00017 | 8.09 |
| 202042_at | histidyl-tRNA synthetase | HARS | 0.00018 | 2.08 |
| 202539_s_at | 3-hydroxy-3-methylglutaryl-CoA reductase | HMGCR | 0.00079 | 4.50 |
| 227110_at | heterogeneous nuclear ribonucleoprotein C (C1/C2) | HNRNPC | 0.00061 | 3.14 |
| 1555844_s_at | heterogeneous nuclear ribonucleoprotein M | HNRNPM | 0.00026 | 2.11 |
| 214328_s_at | heat shock protein 90kDa alpha (cytosolic), class A member 1 | HSP90AA1 | 0.00018 | 1.75 |
| 214434_at | heat shock 70kDa protein 12A | HSPA12A | 0.00034 | 4.59 |
| 202557_at | heat shock protein 70kDa family, member 13 | HSPA13 | 0.00051 | 2.37 |
| 206976_s_at | heat shock 105kDa/110kDa protein 1 | HSPH1 | 0.00005 | 3.70 |
| 202070_s_at | isocitrate dehydrogenase 3 (NAD+) alpha | IDH3A | 0.00043 | 2.23 |
| 230551_at | kinase suppressor of ras 2 | KSR2 | 0.00006 | 5.61 |
| 34764_at | leucyl-tRNA synthetase 2, mitochondrial | LARS2 | 0.00069 | 1.90 |
| 225474_at | membrane associated guanylate kinase, WW and PDZ domain containing 1 | MAGI1 | 0.00032 | 2.37 |
| 206144_at | membrane associated guanylate kinase, WW and PDZ domain containing 1 | MAGI1 | 0.00092 | 2.58 |
| 207121_s_at | mitogen-activated protein kinase 6 | MAPK6 | 0.00064 | 3.01 |
| 201475_x_at | methionyl-tRNA synthetase | MARS | 0.00062 | 1.83 |
| 213671_s_at | methionyl-tRNA synthetase | MARS | 0.00017 | 2.15 |
| 225613_at | microtubule associated serine/threonine kinase family member 4 | MAST4 | 0.00031 | 1.49 |
| 200626_s_at | matrin 3 /// small nucleolar RNA host gene 4 (non-protein coding) | MATR3 /// SNHG4 | 0.00016 | 5.92 |
| 203353_s_at | methyl-CpG binding domain protein 1 | MBD1 | 0.00024 | 1.96 |
| 203095_at | mitochondrial translational initiation factor 2 | MTIF2 | 0.00085 | 2.26 |
| 233665_x_at | mitochondrial tRNA translation optimization 1 | MTO1 | 0.00024 | 2.81 |
| 200027_at | asparaginyl-tRNA synthetase | NARS | 0.00042 | 2.68 |
| 230864_at | serine/threonine-protein kinase NIM1 | NIM1 | 0.00019 | 2.92 |
| 227556_at | NME/NM23 family member 7 | NME7 | 0.00005 | 4.83 |
| 211951_at | nucleolar and coiled-body phosphoprotein 1 | NOLC1 | 0.00089 | 2.01 |
| 225044_at | 5'-nucleotidase, cytosolic IIIB | NT5C3B | 0.00097 | 1.36 |
| 231909_x_at | outer dense fiber of sperm tails 2-like | ODF2L | 0.00014 | 5.74 |
| 219293_s_at | Obg-like ATPase 1 | OLA1 | 0.00003 | 2.85 |
| 214607_at | p21 protein (Cdc42/Rac)-activated kinase 3 | PAK3 | 0.00007 | 2.31 |
| 1555310_a_at | p21 protein (Cdc42/Rac)-activated kinase 6 | PAK6 | 0.00013 | 1.65 |
| 218809_at | pantothenate kinase 2 | PANK2 | 0.00001 | 2.19 |
| 228569_at | poly(A) polymerase alpha | PAPOLA | 0.00075 | 2.43 |
| 222839_s_at | poly(A) polymerase gamma | PAPOLG | 0.00005 | 3.38 |
| 209043_at | 3'-phosphoadenosine 5'-phosphosulfate synthase 1 | PAPSS1 | 0.00024 | 2.99 |
| 207956_x_at | PDS5, regulator of cohesion maintenance, homolog B (S. cerevisiae) | PDS5B | 0.00066 | 2.56 |
| 201037_at | phosphofructokinase, platelet | PFKP | 0.00086 | 2.02 |
| 227068_at | phosphoglycerate kinase 1 | PGK1 | 0.00096 | 3.23 |
| 212740_at | phosphoinositide-3-kinase, regulatory subunit 4 | PIK3R4 | 0.00041 | 3.90 |
| 201939_at | polo-like kinase 2 | PLK2 | 0.00085 | 8.56 |
| 203253_s_at | diphosphoinositol pentakisphosphate kinase 2 | PPIP5K2 | 0.00082 | 2.94 |
| 203680_at | protein kinase, cAMP-dependent, regulatory, type II, beta | PRKAR2B | 0.00018 | 5.26 |
| 226101_at | protein kinase C, epsilon | PRKCE | 0.00011 | 1.80 |
| 203401_at | phosphoribosyl pyrophosphate synthetase 2 | PRPS2 | 0.00064 | 4.92 |
| 219151_s_at | RAB, member of RAS oncogene family-like 2A /// RAB, member of RAS oncogene family-like 2B | RABL2A /// RABL2B | 0.00008 | 3.01 |
| 225585_at | RAP2A, member of RAS oncogene family | RAP2A | 0.00039 | 3.66 |
| 201330_at | arginyl-tRNA synthetase | RARS | 0.00100 | 2.75 |
| 223467_at | RAS, dexamethasone-induced 1 | RASD1 | 0.00024 | 1.87 |
| 219142_at | RAS-like, family 11, member B | RASL11B | 0.00095 | 3.01 |
| 235070_at | RNA binding protein, fox-1 homolog (C. elegans) 1 | RBFOX1 | 0.00033 | 3.78 |
| 221217_s_at | RNA binding protein, fox-1 homolog (C. elegans) 1 | RBFOX1 | 0.00014 | 2.53 |
| 225236_at | RNA binding motif protein 18 | RBM18 | 0.00086 | 2.92 |
| 226316_at | RNA binding motif protein 26 | RBM26 | 0.00005 | 3.24 |
| 229433_at | RNA binding motif protein 26 | RBM26 | 0.00021 | 2.93 |
| 225326_at | RNA binding motif protein 27 | RBM27 | 0.00049 | 2.01 |
| 211421_s_at | ret proto-oncogene | RET | 0.00057 | 3.20 |
| 203225_s_at | riboflavin kinase | RFK | 0.00029 | 3.20 |
| 201477_s_at | ribonucleotide reductase M1 | RRM1 | 0.00037 | 2.45 |
| 213262_at | spastic ataxia of Charlevoix-Saguenay (sacsin) | SACS | 0.00022 | 3.72 |
| 41329_at | SCY1-like 3 (S. cerevisiae) | SCYL3 | 0.00015 | 2.45 |
| 200961_at | selenophosphate synthetase 2 | SEPHS2 | 0.00026 | 2.53 |
| 225227_at | SKI-like oncogene | SKIL | 0.00000 | 2.13 |
| 213720_s_at | SWI/SNF related, matrix associated, actin dependent regulator of chromatin, subfamily a, member 4 | SMARCA4 | 0.00049 | 2.68 |
| 214728_x_at | SWI/SNF related, matrix associated, actin dependent regulator of chromatin, subfamily a, member 4 | SMARCA4 | 0.00054 | 1.80 |
| 208794_s_at | SWI/SNF related, matrix associated, actin dependent regulator of chromatin, subfamily a, member 4 | SMARCA4 | 0.00039 | 2.21 |
| 200058_s_at | small nuclear ribonucleoprotein 200kDa (U5) | SNRNP200 | 0.00047 | 1.60 |
| 1558254_s_at | SRSF protein kinase 2 | SRPK2 | 0.00044 | 2.82 |
| 230091_at | SRSF protein kinase 2 | SRPK2 | 0.00058 | 4.16 |
| 218140_x_at | signal recognition particle receptor, B subunit | SRPRB | 0.00078 | 2.12 |
| 208854_s_at | serine/threonine kinase 24 | STK24 | 0.00027 | 1.62 |
| 231806_s_at | serine/threonine kinase 36 | STK36 | 0.00079 | 2.18 |
| 221696_s_at | serine/threonine/tyrosine kinase 1 | STYK1 | 0.00042 | 2.32 |
| 220030_at | serine/threonine/tyrosine kinase 1 | STYK1 | 0.00039 | 6.77 |
| 202930_s_at | succinate-CoA ligase, ADP-forming, beta subunit | SUCLA2 | 0.00019 | 5.65 |
| 209025_s_at | synaptotagmin binding, cytoplasmic RNA interacting protein | SYNCRIP | 0.00080 | 4.60 |
| 202168_at | TAF9 RNA polymerase II, TATA box binding protein (TBP)-associated factor, 32kDa | TAF9 | 0.00032 | 4.25 |
| 218520_at | TANK-binding kinase 1 | TBK1 | 0.00077 | 3.10 |
| 222931_s_at | threonine synthase-like 1 (S. cerevisiae) | THNSL1 | 0.00039 | 2.13 |
| 209593_s_at | torsin family 1, member B (torsin B) | TOR1B | 0.00041 | 1.76 |
| 201391_at | TNF receptor-associated protein 1 | TRAP1 | 0.00037 | 2.01 |
| 201266_at | thioredoxin reductase 1 | TXNRD1 | 0.00078 | 2.00 |
| 224691_at | U2AF homology motif (UHM) kinase 1 | UHMK1 | 0.00047 | 2.92 |
| 218757_s_at | UPF3 regulator of nonsense transcripts homolog B (yeast) | UPF3B | 0.00006 | 2.24 |
| 203856_at | vaccinia related kinase 1 | VRK1 | 0.00067 | 3.57 |
| 212048_s_at | tyrosyl-tRNA synthetase | YARS | 0.00099 | 2.70 |
|  |  |  |  |  |
| *Miscellaneous/Unknown* | |  |  |  |
| 202169_s_at | aminoadipate-semialdehyde dehydrogenase-phosphopantetheinyl transferase | AASDHPPT | 0.00085 | 7.72 |
| 218633_x_at | abhydrolase domain containing 10 | ABHD10 | 0.00004 | 2.40 |
| 237974_at | abhydrolase domain containing 12B /// microRNA 4454 | ABHD12B /// MIR4454 | 0.00003 | 2.36 |
| 212895_s_at | active BCR-related | ABR | 0.00089 | 1.86 |
| 223361_at | ABRA C-terminal like | ABRACL | 0.00013 | 4.44 |
| 202324_s_at | acyl-CoA binding domain containing 3 | ACBD3 | 0.00010 | 2.09 |
| 204565_at | acyl-CoA thioesterase 13 | ACOT13 | 0.00000 | 2.99 |
| 208002_s_at | acyl-CoA thioesterase 7 | ACOT7 | 0.00033 | 2.60 |
| 206833_s_at | acylphosphatase 2, muscle type | ACYP2 | 0.00036 | 3.73 |
| 228925_at | ADAM metallopeptidase domain 1A, pseudogene /// NULL | ADAM1A /// ADAM1A | 0.00049 | 4.16 |
| 203865_s_at | adenosine deaminase, RNA-specific, B1 | ADARB1 | 0.00011 | 2.73 |
| 225889_at | AE binding protein 2 | AEBP2 | 0.00016 | 2.39 |
| 218534_s_at | angiogenic factor with G patch and FHA domains 1 | AGGF1 | 0.00049 | 3.02 |
| 222661_at | angiogenic factor with G patch and FHA domains 1 | AGGF1 | 0.00077 | 2.24 |
| 203566_s_at | amylo-alpha-1, 6-glucosidase, 4-alpha-glucanotransferase | AGL | 0.00045 | 3.04 |
| 228120_at | argonaute RISC catalytic component 1 | AGO1 | 0.00093 | 1.80 |
| 224480_s_at | 1-acylglycerol-3-phosphate O-acyltransferase 9 | AGPAT9 | 0.00001 | 2.54 |
| 221569_at | Abelson helper integration site 1 | AHI1 | 0.00036 | 3.33 |
| 212992_at | AHNAK nucleoprotein 2 | AHNAK2 | 0.00002 | 3.51 |
| 227605_at | aminoacyl tRNA synthetase complex-interacting multifunctional protein 1 | AIMP1 | 0.00089 | 2.07 |
| 226718_at | adhesion molecule with Ig-like domain 1 | AMIGO1 | 0.00055 | 1.84 |
| 226258_at | antagonist of mitotic exit network 1 homolog (S. cerevisiae) | AMN1 | 0.00026 | 3.02 |
| 212798_s_at | ankyrin repeat and MYND domain containing 2 | ANKMY2 | 0.00042 | 4.32 |
| 1554471_a_at | ankyrin repeat domain 13C | ANKRD13C | 0.00021 | 3.27 |
| 227375_at | ankyrin repeat domain 13C | ANKRD13C | 0.00086 | 3.52 |
| 221522_at | ankyrin repeat domain 27 (VPS9 domain) | ANKRD27 | 0.00056 | 2.60 |
| 216073_at | ankyrin repeat domain 34C | ANKRD34C | 0.00088 | 5.44 |
| 1569040_s_at | ankyrin repeat domain 36B pseudogene 2 /// ankyrin repeat domain-containing protein 36C-like | ANKRD36BP2 /// LOC101060554 | 0.00043 | 6.48 |
| 212731_at | ankyrin repeat domain 46 | ANKRD46 | 0.00027 | 7.25 |
| 225731_at | ankyrin repeat domain 50 | ANKRD50 | 0.00035 | 4.55 |
| 225735_at | ankyrin repeat domain 50 | ANKRD50 | 0.00032 | 2.97 |
| 215241_at | anoctamin 3 | ANO3 | 0.00014 | 3.67 |
| 201366_at | annexin A7 | ANXA7 | 0.00000 | 2.22 |
| 214960_at | apoptosis inhibitor 5 | API5 | 0.00001 | 5.43 |
| 213282_at | apolipoprotein O-like | APOOL | 0.00032 | 1.69 |
| 218158_s_at | adaptor protein, phosphotyrosine interaction, PH domain and leucine zipper containing 1 | APPL1 | 0.00099 | 3.96 |
| 213601_at | Rho GTPase activating protein 19 /// slit homolog 1 (Drosophila) | ARHGAP19 /// SLIT1 | 0.00013 | 2.22 |
| 205414_s_at | Rho GTPase activating protein 44 | ARHGAP44 | 0.00015 | 2.53 |
| 219610_at | Rho guanine nucleotide exchange factor (GEF) 28 | ARHGEF28 | 0.00063 | 1.87 |
| 219610_at | Rho guanine nucleotide exchange factor (GEF) 28 | ARHGEF28 | 0.00063 | 1.87 |
| 225181_at | AT rich interactive domain 1B (SWI1-like) | ARID1B | 0.00096 | 1.59 |
| 1555279_at | armadillo repeat containing 8 | ARMC8 | 0.00064 | 7.91 |
| 218694_at | armadillo repeat containing, X-linked 1 | ARMCX1 | 0.00074 | 2.74 |
| 203404_at | armadillo repeat containing, X-linked 2 | ARMCX2 | 0.00055 | 3.77 |
| 228027_at | ARMCX5-GPRASP2 readthrough /// G protein-coupled receptor associated sorting protein 2 | ARMCX5-GPRASP2 /// GPRASP2 | 0.00081 | 3.24 |
| 221483_s_at | cAMP-regulated phosphoprotein, 19kDa | ARPP19 | 0.00091 | 2.00 |
| 220359_s_at | cAMP-regulated phosphoprotein, 21kDa | ARPP21 | 0.00009 | 6.22 |
| 231935_at | cAMP-regulated phosphoprotein, 21kDa | ARPP21 | 0.00057 | 4.25 |
| 43511_s_at | arrestin, beta 1 | ARRB1 | 0.00071 | 1.76 |
| 222667_s_at | ash1 (absent, small, or homeotic)-like (Drosophila) | ASH1L | 0.00062 | 1.53 |
| 227014_at | aspartate beta-hydroxylase domain containing 2 | ASPHD2 | 0.00070 | 2.36 |
| 227365_at | ataxia, cerebellar, Cayman type | ATCAY | 0.00021 | 1.66 |
| 226684_at | autophagy related 2B | ATG2B | 0.00049 | 3.26 |
| 223338_s_at | ATPase inhibitory factor 1 | ATPIF1 | 0.00030 | 1.85 |
| 213744_at | attractin-like 1 | ATRNL1 | 0.00070 | 2.80 |
| 213745_at | attractin-like 1 | ATRNL1 | 0.00004 | 13.47 |
| 212474_at | AVL9 homolog (S. cerevisiase) | AVL9 | 0.00025 | 3.35 |
| 211379_x_at | beta-1,3-N-acetylgalactosaminyltransferase 1 (globoside blood group) | B3GALNT1 | 0.00033 | 6.42 |
| 202984_s_at | BCL2-associated athanogene 5 | BAG5 | 0.00004 | 4.03 |
| 202985_s_at | BCL2-associated athanogene 5 | BAG5 | 0.00010 | 2.39 |
| 205638_at | brain-specific angiogenesis inhibitor 3 | BAI3 | 0.00056 | 5.02 |
| 205294_at | BAI1-associated protein 2 | BAIAP2 | 0.00016 | 2.73 |
| 225285_at | branched chain amino-acid transaminase 1, cytosolic | BCAT1 | 0.00042 | 3.76 |
| 210347_s_at | B-cell CLL/lymphoma 11A (zinc finger protein) | BCL11A | 0.00070 | 2.41 |
| 201084_s_at | BCL2-associated transcription factor 1 | BCLAF1 | 0.00084 | 3.90 |
| 218332_at | brain expressed, X-linked 1 | BEX1 | 0.00017 | 4.50 |
| 224367_at | brain expressed X-linked 2 | BEX2 | 0.00044 | 2.82 |
| 229963_at | brain expressed, X-linked 5 | BEX5 | 0.00016 | 4.77 |
| 213709_at | basic helix-loop-helix domain containing, class B, 9 | BHLHB9 | 0.00001 | 4.20 |
| 231964_at | bicaudal D homolog 1 (Drosophila) | BICD1 | 0.00049 | 4.68 |
| 222761_at | basic, immunoglobulin-like variable motif containing | BIVM | 0.00057 | 2.76 |
| 201032_at | bladder cancer associated protein | BLCAP | 0.00082 | 2.48 |
| 226580_at | breast cancer metastasis-suppressor 1-like | BRMS1L | 0.00063 | 2.88 |
| 225217_s_at | bromodomain and PHD finger containing, 3 | BRPF3 | 0.00003 | 2.10 |
| 208906_at | Berardinelli-Seip congenital lipodystrophy 2 (seipin) /// HNRNPUL2-BSCL2 readthrough | BSCL2 /// HNRNPUL2-BSCL2 | 0.00003 | 4.05 |
| 225811_at | chromosome 11 open reading frame 58 | C11orf58 | 0.00017 | 2.93 |
| 230326_s_at | chromosome 11 open reading frame 73 | C11orf73 | 0.00051 | 2.45 |
| 1557180_at | chromosome 11 open reading frame 87 | C11orf87 | 0.00078 | 6.97 |
| 236532_at | chromosome 11 open reading frame 87 | C11orf87 | 0.00009 | 4.49 |
| 218220_at | chromosome 12 open reading frame 10 | C12orf10 | 0.00002 | 2.44 |
| 222613_at | chromosome 12 open reading frame 4 | C12orf4 | 0.00096 | 2.01 |
| 219099_at | chromosome 12 open reading frame 5 | C12orf5 | 0.00028 | 3.88 |
| 44065_at | chromosome 12 open reading frame 52 | C12orf52 | 0.00001 | 3.39 |
| 229071_at | chromosome 17 open reading frame 100 | C17orf100 | 0.00007 | 2.18 |
| 226406_at | chromosome 18 open reading frame 25 | C18orf25 | 0.00001 | 3.36 |
| 53720_at | chromosome 19 open reading frame 66 | C19orf66 | 0.00075 | 1.80 |
| 218712_at | chromosome 1 open reading frame 109 | C1orf109 | 0.00050 | 2.61 |
| 229973_at | chromosome 1 open reading frame 173 | C1orf173 | 0.00028 | 10.97 |
| 221272_s_at | chromosome 1 open reading frame 21 | C1orf21 | 0.00032 | 2.03 |
| 212791_at | chromosome 1 open reading frame 216 | C1orf216 | 0.00072 | 3.03 |
| 214214_s_at | complement component 1, q subcomponent binding protein | C1QBP | 0.00007 | 5.62 |
| 225224_at | chromosome 20 open reading frame 112 | C20orf112 | 0.00052 | 1.89 |
| 212943_at | C2 calcium-dependent domain containing 5 | C2CD5 | 0.00080 | 2.63 |
| 238974_at | chromosome 2 open reading frame 69 | C2orf69 | 0.00004 | 3.84 |
| 236068_s_at | chromosome 3 open reading frame 14 | C3orf14 | 0.00029 | 3.18 |
| 201678_s_at | chromosome 3 open reading frame 37 | C3orf37 | 0.00099 | 1.90 |
| 203738_at | chromosome 5 open reading frame 22 | C5orf22 | 0.00085 | 2.49 |
| 221787_at | chromosome 6 open reading frame 120 | C6orf120 | 0.00068 | 3.74 |
| 227456_s_at | chromosome 6 open reading frame 136 | C6orf136 | 0.00064 | 2.48 |
| 241024_at | chromosome 6 open reading frame 147 /// KH homology domain containing 1 | C6orf147 /// KHDC1 | 0.00054 | 3.72 |
| 223576_at | chromosome 6 open reading frame 203 | C6orf203 | 0.00085 | 2.65 |
| 53202_at | chromosome 7 open reading frame 25 /// proteasome (prosome, macropain) subunit, alpha type, 2 | C7orf25 /// PSMA2 | 0.00058 | 3.78 |
| 47083_at | chromosome 7 open reading frame 26 | C7orf26 | 0.00083 | 1.44 |
| 228149_at | chromosome 7 open reading frame 60 | C7orf60 | 0.00045 | 3.85 |
| 218187_s_at | chromosome 8 open reading frame 33 | C8orf33 | 0.00033 | 2.48 |
| 229430_at | chromosome 8 open reading frame 46 | C8orf46 | 0.00044 | 2.22 |
| 221865_at | chromosome 9 open reading frame 91 | C9orf91 | 0.00006 | 3.86 |
| 223550_s_at | carbonic anhydrase X | CA10 | 0.00026 | 6.15 |
| 231270_at | carbonic anhydrase XIII /// uncharacterized LOC100507258 | CA13 /// LOC100507258 | 0.00076 | 2.65 |
| 217873_at | calcium binding protein 39 | CAB39 | 0.00042 | 5.46 |
| 235118_at | cell adhesion molecule 2 | CADM2 | 0.00055 | 2.78 |
| 212551_at | CAP, adenylate cyclase-associated protein, 2 (yeast) | CAP2 | 0.00035 | 2.71 |
| 212554_at | CAP, adenylate cyclase-associated protein, 2 (yeast) | CAP2 | 0.00062 | 10.95 |
| 224370_s_at | calcyphosine 2 | CAPS2 | 0.00001 | 6.29 |
| 217940_s_at | carbohydrate kinase domain containing | CARKD | 0.00096 | 1.98 |
| 219342_at | CAS1 domain containing 1 | CASD1 | 0.00002 | 5.06 |
| 242301_at | cerebellin 2 precursor | CBLN2 | 0.00025 | 4.52 |
| 206037_at | cysteine conjugate-beta lyase, cytoplasmic | CCBL1 | 0.00054 | 1.43 |
| 206721_at | coiled-coil domain containing 181 | CCDC181 | 0.00006 | 3.49 |
| 218125_s_at | coiled-coil domain containing 25 | CCDC25 | 0.00010 | 2.00 |
| 218628_at | coiled-coil domain containing 53 | CCDC53 | 0.00033 | 1.63 |
| 228122_at | coiled-coil domain containing 66 | CCDC66 | 0.00022 | 2.69 |
| 223301_s_at | coiled-coil domain containing 82 | CCDC82 | 0.00022 | 2.60 |
| 235228_at | coiled-coil domain containing 85A | CCDC85A | 0.00099 | 4.80 |
| 205827_at | cholecystokinin | CCK | 0.00006 | 6.35 |
| 201955_at | cyclin C | CCNC | 0.00079 | 3.81 |
| 202769_at | cyclin G2 | CCNG2 | 0.00069 | 1.41 |
| 213743_at | cyclin T2 | CCNT2 | 0.00048 | 2.19 |
| 224649_x_at | cyclin Y | CCNY | 0.00045 | 3.65 |
| 227280_s_at | cyclin Y-like 1 | CCNYL1 | 0.00023 | 3.11 |
| 221156_x_at | cell cycle progression 1 | CCPG1 | 0.00028 | 2.20 |
| 214151_s_at | cell cycle progression 1 /// DYX1C1-CCPG1 readthrough (NMD candidate) | CCPG1 /// DYX1C1-CCPG1 | 0.00084 | 3.51 |
| 208310_s_at | CCZ1 vacuolar protein trafficking and biogenesis associated homolog (S. cerevisiae) /// CCZ1 vacuolar protein trafficking and biogenesis associated homolog B (S. cerevisiae) | CCZ1 /// CCZ1B | 0.00060 | 1.41 |
| 209582_s_at | CD200 molecule | CD200 | 0.00017 | 3.49 |
| 209583_s_at | CD200 molecule | CD200 | 0.00080 | 6.03 |
| 203376_at | cell division cycle 40 | CDC40 | 0.00025 | 3.07 |
| 220115_s_at | cadherin 10, type 2 (T2-cadherin) | CDH10 | 0.00001 | 3.69 |
| 207149_at | cadherin 12, type 2 (N-cadherin 2) | CDH12 | 0.00091 | 3.66 |
| 204726_at | cadherin 13, H-cadherin (heart) | CDH13 | 0.00003 | 6.23 |
| 206280_at | cadherin 18, type 2 | CDH18 | 0.00049 | 2.40 |
| 241500_at | cadherin 8, type 2 | CDH8 | 0.00003 | 5.83 |
| 204154_at | cysteine dioxygenase type 1 | CDO1 | 0.00074 | 4.12 |
| 225527_at | CCAAT/enhancer binding protein (C/EBP), gamma | CEBPG | 0.00046 | 2.02 |
| 212446_s_at | ceramide synthase 6 | CERS6 | 0.00013 | 7.09 |
| 223753_s_at | cripto, FRL-1, cryptic family 1 /// cripto, FRL-1, cryptic family 1B | CFC1 /// CFC1B | 0.00003 | 1.87 |
| 204605_at | cell growth regulator with ring finger domain 1 | CGRRF1 | 0.00077 | 1.94 |
| 224932_at | coiled-coil-helix-coiled-coil-helix domain containing 10 | CHCHD10 | 0.00069 | 1.91 |
| 217972_at | coiled-coil-helix-coiled-coil-helix domain containing 3 | CHCHD3 | 0.00095 | 1.50 |
| 204260_at | chromogranin B (secretogranin 1) | CHGB | 0.00023 | 8.06 |
| 228345_at | cysteine-rich hydrophobic domain 1 | CHIC1 | 0.00010 | 3.43 |
| 204591_at | cell adhesion molecule with homology to L1CAM (close homolog of L1) | CHL1 | 0.00073 | 6.12 |
| 212624_s_at | chimerin 1 | CHN1 | 0.00043 | 5.43 |
| 242488_at | cholinergic receptor, muscarinic 3 | CHRM3 | 0.00021 | 4.17 |
| 224903_at | cirrhosis, autosomal recessive 1A (cirhin) | CIRH1A | 0.00067 | 2.07 |
| 226686_at | CDGSH iron sulfur domain 2 | CISD2 | 0.00040 | 3.16 |
| 201769_at | clathrin interactor 1 | CLINT1 | 0.00023 | 2.25 |
| 225856_at | clock circadian regulator | CLOCK | 0.00001 | 2.26 |
| 227531_at | clock circadian regulator | CLOCK | 0.00016 | 3.58 |
| 205518_s_at | cytidine monophospho-N-acetylneuraminic acid hydroxylase, pseudogene | CMAHP | 0.00015 | 2.25 |
| 227731_at | CCHC-type zinc finger, nucleic acid binding protein | CNBP | 0.00018 | 2.54 |
| 201653_at | cornichon homolog (Drosophila) | CNIH | 0.00011 | 3.01 |
| 213436_at | cannabinoid receptor 1 (brain) | CNR1 | 0.00036 | 15.39 |
| 227202_at | contactin 1 | CNTN1 | 0.00032 | 3.84 |
| 229831_at | contactin 3 (plasmacytoma associated) | CNTN3 | 0.00068 | 3.91 |
| 219301_s_at | contactin associated protein-like 2 | CNTNAP2 | 0.00017 | 2.94 |
| 225638_at | cytochrome c oxidase assembly factor 6 homolog (S. cerevisiae) | COA6 | 0.00030 | 2.13 |
| 222637_at | COMM domain containing 10 | COMMD10 | 0.00066 | 3.70 |
| 218072_at | COMM domain containing 9 | COMMD9 | 0.00002 | 2.18 |
| 213486_at | COPG2 imprinted transcript 1 (non-protein coding) | COPG2IT1 | 0.00003 | 5.69 |
| 225096_at | coordinator of PRMT5, differentiation stimulator | COPRS | 0.00090 | 2.61 |
| 202078_at | COP9 signalosome subunit 3 | COPS3 | 0.00025 | 2.30 |
| 218042_at | COP9 signalosome subunit 4 | COPS4 | 0.00043 | 4.58 |
| 202141_s_at | COP9 signalosome subunit 8 | COPS8 | 0.00100 | 2.01 |
| 223515_s_at | coenzyme Q3 methyltransferase | COQ3 | 0.00011 | 3.26 |
| 1552301_a_at | coronin 6 | CORO6 | 0.00051 | 2.60 |
| 231336_at | copine IV | CPNE4 | 0.00012 | 4.13 |
| 201989_s_at | cAMP responsive element binding protein-like 2 | CREBL2 | 0.00021 | 2.10 |
| 201990_s_at | cAMP responsive element binding protein-like 2 | CREBL2 | 0.00027 | 3.10 |
| 235556_at | CREB3 regulatory factor | CREBRF | 0.00069 | 2.29 |
| 221260_s_at | cysteine-serine-rich nuclear protein 2 | CSRNP2 | 0.00000 | 4.35 |
| 235355_at | cysteine-serine-rich nuclear protein 3 | CSRNP3 | 0.00050 | 4.72 |
| 203947_at | cleavage stimulation factor, 3' pre-RNA, subunit 3, 77kDa | CSTF3 | 0.00100 | 2.12 |
| 225681_at | collagen triple helix repeat containing 1 | CTHRC1 | 0.00041 | 2.96 |
| 222706_at | CWC25 spliceosome-associated protein homolog (S. cerevisiae) | CWC25 | 0.00092 | 2.14 |
| 223337_at | CWC27 spliceosome-associated protein homolog (S. cerevisiae) | CWC27 | 0.00036 | 1.65 |
| 238554_at | cytochrome b5 type B (outer mitochondrial membrane) | CYB5B | 0.00039 | 4.19 |
| 209975_at | cytochrome P450, family 2, subfamily E, polypeptide 1 | CYP2E1 | 0.00031 | 2.21 |
| 224748_at | DDB1 and CUL4 associated factor 7 | DCAF7 | 0.00004 | 1.55 |
| 238914_at | deleted in colorectal carcinoma | DCC | 0.00007 | 3.69 |
| 1557617_at | DCTN1 antisense RNA 1 | DCTN1-AS1 | 0.00062 | 3.14 |
| 239425_at | DCN1, defective in cullin neddylation 1, domain containing 5 | DCUN1D5 | 0.00016 | 2.52 |
| 228032_s_at | DENN/MADD domain containing 1B | DENND1B | 0.00026 | 3.13 |
| 228551_at | DENN/MADD domain containing 5B | DENND5B | 0.00094 | 2.93 |
| 220251_at | digestive organ expansion factor homolog (zebrafish) | DIEXF | 0.00009 | 2.68 |
| 201479_at | dyskeratosis congenita 1, dyskerin /// microRNA 644b /// small nucleolar RNA, H/ACA box 56 | DKC1 /// MIR644B /// SNORA56 | 0.00044 | 2.02 |
| 235527_at | discs, large (Drosophila) homolog-associated protein 1 | DLGAP1 | 0.00030 | 5.42 |
| 203791_at | Dmx-like 1 | DMXL1 | 0.00057 | 3.42 |
| 225061_at | DnaJ (Hsp40) homolog, subfamily A, member 4 | DNAJA4 | 0.00026 | 3.00 |
| 208810_at | DnaJ (Hsp40) homolog, subfamily B, member 6 /// transmembrane protein 135 | DNAJB6 /// TMEM135 | 0.00096 | 5.28 |
| 218976_at | DnaJ (Hsp40) homolog, subfamily C, member 12 | DNAJC12 | 0.00022 | 3.98 |
| 212908_at | DnaJ (Hsp40) homolog, subfamily C, member 16 | DNAJC16 | 0.00063 | 2.28 |
| 213853_at | DnaJ (Hsp40) homolog, subfamily C, member 24 | DNAJC24 | 0.00031 | 2.82 |
| 226859_at | DnaJ (Hsp40) homolog, subfamily C , member 25 /// DNAJC25-GNG10 readthrough | DNAJC25 /// DNAJC25-GNG10 | 0.00008 | 3.23 |
| 212490_at | DnaJ (Hsp40) homolog, subfamily C, member 8 | DNAJC8 | 0.00071 | 2.60 |
| 213088_s_at | DnaJ (Hsp40) homolog, subfamily C, member 9 | DNAJC9 | 0.00062 | 2.87 |
| 1553976_a_at | deleted in primary ciliary dyskinesia homolog (mouse) | DPCD | 0.00003 | 2.52 |
| 222360_at | diphthamide biosynthesis 5 | DPH5 | 0.00032 | 2.88 |
| 230158_at | dpy-19-like 2 (C. elegans) | DPY19L2 | 0.00003 | 3.39 |
| 215143_at | dpy-19-like 2 pseudogene 2 (C. elegans) | DPY19L2P2 | 0.00026 | 4.03 |
| 235721_at | deltex homolog 3 (Drosophila) | DTX3 | 0.00004 | 1.35 |
| 208955_at | deoxyuridine triphosphatase | DUT | 0.00015 | 1.90 |
| 223171_at | dymeclin | DYM | 0.00063 | 2.49 |
| 217992_s_at | EF-hand domain family, member D2 | EFHD2 | 0.00088 | 1.97 |
| 225936_at | EP300 interacting inhibitor of differentiation 2 | EID2 | 0.00085 | 3.72 |
| 208985_s_at | eukaryotic translation initiation factor 3, subunit J | EIF3J | 0.00013 | 2.25 |
| 242674_at | eukaryotic translation initiation factor 4E | EIF4E | 0.00019 | 3.86 |
| 226734_at | eukaryotic translation initiation factor 4E family member 2 | EIF4E2 | 0.00086 | 2.59 |
| 225941_at | eukaryotic translation initiation factor 4E family member 3 | EIF4E3 | 0.00001 | 4.17 |
| 217956_s_at | enolase-phosphatase 1 | ENOPH1 | 0.00030 | 4.19 |
| 237054_at | ectonucleotide pyrophosphatase/phosphodiesterase 5 (putative) | ENPP5 | 0.00002 | 4.17 |
| 204076_at | ectonucleoside triphosphate diphosphohydrolase 4 | ENTPD4 | 0.00017 | 3.79 |
| 223253_at | ependymin related protein 1 (zebrafish) | EPDR1 | 0.00011 | 12.39 |
| 228453_at | ectopic P-granules autophagy protein 5 homolog (C. elegans) | EPG5 | 0.00048 | 2.31 |
| 239579_at | epoxide hydrolase 4 | EPHX4 | 0.00082 | 5.86 |
| 227847_at | EPM2A (laforin) interacting protein 1 | EPM2AIP1 | 0.00018 | 3.04 |
| 218135_at | ERGIC and golgi 2 | ERGIC2 | 0.00080 | 2.55 |
| 224657_at | ERBB receptor feedback inhibitor 1 | ERRFI1 | 0.00015 | 2.36 |
| 207981_s_at | estrogen-related receptor gamma | ESRRG | 0.00014 | 4.56 |
| 227200_at | ets variant 3 | ETV3 | 0.00015 | 1.81 |
| 212108_at | Fas associated factor family member 2 | FAF2 | 0.00061 | 1.55 |
| 222673_x_at | family with sequence similarity 122B | FAM122B | 0.00005 | 3.14 |
| 225361_x_at | family with sequence similarity 122B | FAM122B | 0.00003 | 3.07 |
| 229460_at | family with sequence similarity 126, member B | FAM126B | 0.00015 | 5.63 |
| 221904_at | family with sequence similarity 131, member A | FAM131A | 0.00097 | 2.14 |
| 221983_at | family with sequence similarity 134, member A | FAM134A | 0.00011 | 2.93 |
| 221984_s_at | family with sequence similarity 134, member A | FAM134A | 0.00065 | 2.27 |
| 218510_x_at | family with sequence similarity 134, member B | FAM134B | 0.00024 | 2.57 |
| 218532_s_at | family with sequence similarity 134, member B | FAM134B | 0.00082 | 3.43 |
| 218518_at | family with sequence similarity 13, member B | FAM13B | 0.00033 | 3.16 |
| 214945_at | family with sequence similarity 153, member A /// family with sequence similarity 153, member B /// family with sequence similarity 153, member C, pseudogene /// uncharacterized LOC100507387 | FAM153A /// FAM153B /// FAM153C /// LOC100507387 | 0.00006 | 2.72 |
| 242584_at | family with sequence similarity 161, member A | FAM161A | 0.00008 | 2.30 |
| 36612_at | family with sequence similarity 168, member A | FAM168A | 0.00053 | 1.88 |
| 241399_at | family with sequence similarity 19 (chemokine (C-C motif)-like), member A2 | FAM19A2 | 0.00043 | 4.36 |
| 229459_at | family with sequence similarity 19 (chemokine (C-C motif)-like), member A5 | FAM19A5 | 0.00045 | 4.48 |
| 218331_s_at | family with sequence similarity 208, member B | FAM208B | 0.00011 | 2.70 |
| 224435_at | family with sequence similarity 213, member A | FAM213A | 0.00099 | 3.37 |
| 204521_at | family with sequence similarity 216, member A | FAM216A | 0.00076 | 3.88 |
| 224452_s_at | family with sequence similarity 220, member A | FAM220A | 0.00018 | 3.65 |
| 230276_at | family with sequence similarity 49, member A | FAM49A | 0.00059 | 4.43 |
| 228987_at | family with sequence similarity 49, member B | FAM49B | 0.00010 | 3.97 |
| 214822_at | family with sequence similarity 5, member B | FAM5B | 0.00007 | 1.70 |
| 213689_x_at | family with sequence similarity 69, member A | FAM69A | 0.00005 | 2.50 |
| 241456_at | family with sequence similarity 78, member B | FAM78B | 0.00061 | 2.81 |
| 225667_s_at | family with sequence similarity 84, member A | FAM84A | 0.00013 | 3.76 |
| 203420_at | family with sequence similarity 8, member A1 | FAM8A1 | 0.00079 | 1.98 |
| 228011_at | family with sequence similarity 92, member A1 | FAM92A1 | 0.00045 | 2.08 |
| 235391_at | family with sequence similarity 92, member A1 | FAM92A1 | 0.00063 | 3.19 |
| 220615_s_at | fatty acyl CoA reductase 2 | FAR2 | 0.00006 | 5.09 |
| 239108_at | fatty acyl CoA reductase 2 | FAR2 | 0.00023 | 5.32 |
| 224644_at | FGD5 antisense RNA 1 | FGD5-AS1 | 0.00049 | 1.39 |
| 203033_x_at | fumarate hydratase | FH | 0.00089 | 1.94 |
| 214170_x_at | fumarate hydratase | FH | 0.00020 | 2.25 |
| 203656_at | FIG4 homolog, SAC1 lipid phosphatase domain containing (S. cerevisiae) | FIG4 | 0.00012 | 3.34 |
| 222853_at | fibronectin leucine rich transmembrane protein 3 | FLRT3 | 0.00025 | 2.40 |
| 226096_at | fibronectin type III domain containing 5 | FNDC5 | 0.00064 | 2.11 |
| 218503_at | focadhesin | FOCAD | 0.00002 | 2.28 |
| 206018_at | forkhead box G1 | FOXG1 | 0.00020 | 2.38 |
| 206015_s_at | forkhead box J3 | FOXJ3 | 0.00028 | 2.13 |
| 226715_at | forkhead box K1 | FOXK1 | 0.00067 | 1.63 |
| 238596_at | fragile site, folic acid type, rare, fra(10)(q23.3) or fra(10)(q24.2) candidate 1 | FRA10AC1 | 0.00046 | 1.85 |
| 209702_at | fat mass and obesity associated | FTO | 0.00054 | 2.12 |
| 205324_s_at | FtsJ RNA methyltransferase homolog 1 (E. coli) | FTSJ1 | 0.00074 | 2.07 |
| 235960_at | fragile X mental retardation, autosomal homolog 1 | FXR1 | 0.00087 | 4.79 |
| 224641_at | forty-two-three domain containing 1 | FYTTD1 | 0.00041 | 2.39 |
| 239082_at | frizzled family receptor 3 | FZD3 | 0.00012 | 1.65 |
| 212802_s_at | GTPase activating protein and VPS9 domains 1 | GAPVD1 | 0.00034 | 1.97 |
| 218912_at | GRIP and coiled-coil domain containing 1 | GCC1 | 0.00031 | 1.32 |
| 226269_at | ganglioside induced differentiation associated protein 1 | GDAP1 | 0.00089 | 3.83 |
| 224808_s_at | golgi to ER traffic protein 4 homolog (S. cerevisiae) | GET4 | 0.00056 | 1.65 |
| 226886_at | glutamine--fructose-6-phosphate transaminase 1 | GFPT1 | 0.00003 | 3.93 |
| 203560_at | gamma-glutamyl hydrolase (conjugase, folylpolygammaglutamyl hydrolase) | GGH | 0.00066 | 4.60 |
| 233936_s_at | gametogenetin binding protein 2 | GGNBP2 | 0.00026 | 1.78 |
| 226470_at | gamma-glutamyltransferase 7 | GGT7 | 0.00085 | 1.58 |
| 225376_at | GID complex subunit 8 homolog (S. cerevisiae) | GID8 | 0.00039 | 1.88 |
| 206102_at | GINS complex subunit 1 (Psf1 homolog) | GINS1 | 0.00030 | 3.17 |
| 225558_at | G protein-coupled receptor kinase interacting ArfGAP 2 | GIT2 | 0.00072 | 2.52 |
| 225706_at | glucocorticoid induced transcript 1 | GLCCI1 | 0.00022 | 2.65 |
| 227525_at | glucocorticoid induced transcript 1 | GLCCI1 | 0.00034 | 4.94 |
| 206662_at | glutaredoxin (thioltransferase) | GLRX | 0.00026 | 2.50 |
| 203159_at | glutaminase | GLS | 0.00019 | 4.64 |
| 214071_at | guanine nucleotide binding protein (G protein), alpha activating activity polypeptide, olfactory type | GNAL | 0.00095 | 2.57 |
| 204000_at | guanine nucleotide binding protein (G protein), beta 5 | GNB5 | 0.00044 | 1.44 |
| 201921_at | guanine nucleotide binding protein (G protein), gamma 10 | GNG10 | 0.00095 | 1.65 |
| 227022_at | glucosamine-6-phosphate deaminase 2 | GNPDA2 | 0.00002 | 2.57 |
| 202106_at | golgin A3 | GOLGA3 | 0.00030 | 1.92 |
| 217771_at | golgi membrane protein 1 | GOLM1 | 0.00065 | 3.13 |
| 208813_at | glutamic-oxaloacetic transaminase 1, soluble | GOT1 | 0.00021 | 3.62 |
| 208308_s_at | glucose-6-phosphate isomerase | GPI | 0.00064 | 3.41 |
| 232195_at | G protein-coupled receptor 158 | GPR158 | 0.00012 | 4.65 |
| 244493_at | G protein-coupled receptor 22 | GPR22 | 0.00013 | 9.45 |
| 220313_at | G protein-coupled receptor 88 | GPR88 | 0.00002 | 6.44 |
| 222830_at | grainyhead-like 1 (Drosophila) | GRHL1 | 0.00039 | 3.31 |
| 223239_at | GSK3B interacting protein | GSKIP | 0.00003 | 5.60 |
| 218343_s_at | general transcription factor IIIC, polypeptide 3, 102kDa | GTF3C3 | 0.00028 | 1.67 |
| 225543_at | general transcription factor IIIC, polypeptide 4, 90kDa | GTF3C4 | 0.00097 | 2.99 |
| 204237_at | GULP, engulfment adaptor PTB domain containing 1 | GULP1 | 0.00028 | 5.11 |
| 207168_s_at | H2A histone family, member Y | H2AFY | 0.00088 | 2.37 |
| 203745_at | holocytochrome c synthase | HCCS | 0.00051 | 2.90 |
| 228813_at | histone deacetylase 4 | HDAC4 | 0.00026 | 2.07 |
| 209524_at | hepatoma-derived growth factor, related protein 3 | HDGFRP3 | 0.00052 | 1.91 |
| 223155_at | haloacid dehalogenase-like hydrolase domain containing 2 | HDHD2 | 0.00097 | 1.89 |
| 225012_at | high density lipoprotein binding protein | HDLBP | 0.00027 | 1.82 |
| 233642_s_at | HEAT repeat containing 5B | HEATR5B | 0.00064 | 3.39 |
| 217845_x_at | HIG1 hypoxia inducible domain family, member 1A | HIGD1A | 0.00088 | 2.13 |
| 221896_s_at | HIG1 hypoxia inducible domain family, member 1A | HIGD1A | 0.00086 | 2.34 |
| 242317_at | HIG1 hypoxia inducible domain family, member 1A | HIGD1A | 0.00004 | 5.75 |
| 204753_s_at | hepatic leukemia factor | HLF | 0.00085 | 5.36 |
| 222396_at | hematological and neurological expressed 1 | HN1 | 0.00001 | 2.86 |
| 225405_at | heterogeneous nuclear ribonucleoprotein U-like 2 | HNRNPUL2 | 0.00092 | 2.07 |
| 219671_at | hippocalcin like 4 | HPCAL4 | 0.00030 | 2.80 |
| 219697_at | heparan sulfate (glucosamine) 3-O-sulfotransferase 2 | HS3ST2 | 0.00032 | 3.70 |
| 232275_s_at | heparan sulfate 6-O-sulfotransferase 3 | HS6ST3 | 0.00079 | 5.64 |
| 232276_at | heparan sulfate 6-O-sulfotransferase 3 | HS6ST3 | 0.00000 | 1.85 |
| 209657_s_at | heat shock transcription factor 2 | HSF2 | 0.00077 | 2.81 |
| 244130_at | 5-hydroxytryptamine (serotonin) receptor 2A, G protein-coupled | HTR2A | 0.00095 | 7.26 |
| 218611_at | immediate early response 5 | IER5 | 0.00014 | 2.71 |
| 203153_at | interferon-induced protein with tetratricopeptide repeats 1 | IFIT1 | 0.00007 | 5.10 |
| 226977_at | IgA-inducing protein homolog (Bos taurus) | IGIP | 0.00013 | 2.23 |
| 224743_at | inositol monophosphatase domain containing 1 | IMPAD1 | 0.00010 | 2.52 |
| 208415_x_at | inhibitor of growth family, member 1 | ING1 | 0.00099 | 1.84 |
| 242293_at | inhibitor of growth family, member 3 | ING3 | 0.00052 | 1.91 |
| 228287_at | inhibitor of growth family, member 5 | ING5 | 0.00013 | 1.84 |
| 203607_at | inositol polyphosphate-5-phosphatase F | INPP5F | 0.00060 | 3.42 |
| 229632_s_at | integrator complex subunit 10 | INTS10 | 0.00002 | 1.94 |
| 213447_at | imprinted in Prader-Willi syndrome (non-protein coding) /// uncharacterized LOC100506948 /// small nucleolar RNA, C/D box 107 /// small nucleolar RNA, C/D box 115-13 /// small nucleolar RNA, C/D box 115-26 /// small nucleolar RNA, C/D box 115-7 /// small nucleolar RNA, C/D box 116-28 /// small nuclear ribonucleoprotein polypeptide N | IPW /// LOC100506948 /// SNORD107 /// SNORD115-13 /// SNORD115-26 /// SNORD115-7 /// SNORD116-28 /// SNRPN | 0.00094 | 2.00 |
| 204202_at | IQ motif containing E | IQCE | 0.00098 | 2.36 |
| 204030_s_at | IQCJ-SCHIP1 readthrough /// schwannomin interacting protein 1 | IQCJ-SCHIP1 /// SCHIP1 | 0.00091 | 2.81 |
| 203906_at | IQ motif and Sec7 domain 1 | IQSEC1 | 0.00082 | 2.27 |
| 203907_s_at | IQ motif and Sec7 domain 1 | IQSEC1 | 0.00063 | 2.06 |
| 1556151_at | integrin alpha FG-GAP repeat containing 1 | ITFG1 | 0.00024 | 1.92 |
| 226295_at | integrin alpha FG-GAP repeat containing 2 /// uncharacterized LOC100507424 | ITFG2 /// LOC100507424 | 0.00070 | 1.59 |
| 223104_at | jagunal homolog 1 (Drosophila) | JAGN1 | 0.00078 | 2.01 |
| 228793_at | jumonji domain containing 1C | JMJD1C | 0.00047 | 2.61 |
| 204301_at | kelch repeat and BTB (POZ) domain containing 11 | KBTBD11 | 0.00002 | 2.67 |
| 226479_at | kelch repeat and BTB (POZ) domain containing 6 | KBTBD6 | 0.00002 | 5.38 |
| 229298_at | kelch repeat and BTB (POZ) domain containing 7 | KBTBD7 | 0.00023 | 2.75 |
| 229970_at | kelch repeat and BTB (POZ) domain containing 7 | KBTBD7 | 0.00021 | 6.23 |
| 239118_at | potassium voltage-gated channel, shaker-related subfamily, member 2 | KCNA2 | 0.00076 | 2.63 |
| 210078_s_at | potassium voltage-gated channel, shaker-related subfamily, beta member 1 | KCNAB1 | 0.00055 | 4.47 |
| 231053_at | potassium voltage-gated channel, Shab-related subfamily, member 1 | KCNB1 | 0.00017 | 2.09 |
| 232401_at | potassium voltage-gated channel, delayed-rectifier, subfamily S, member 2 | KCNS2 | 0.00040 | 3.54 |
| 209984_at | lysine (K)-specific demethylase 4C | KDM4C | 0.00093 | 2.28 |
| 229850_at | 3-ketodihydrosphingosine reductase | KDSR | 0.00067 | 2.15 |
| 209781_s_at | KH domain containing, RNA binding, signal transduction associated 3 | KHDRBS3 | 0.00042 | 1.84 |
| 230249_at | KH domain containing, RNA binding, signal transduction associated 3 | KHDRBS3 | 0.00013 | 2.29 |
| 206017_at | KIAA0319 | KIAA0319 | 0.00002 | 2.97 |
| 204546_at | KIAA0513 | KIAA0513 | 0.00053 | 3.24 |
| 213424_at | KIAA0895 | KIAA0895 | 0.00089 | 4.17 |
| 213636_at | KIAA1045 | KIAA1045 | 0.00005 | 2.88 |
| 37566_at | KIAA1045 | KIAA1045 | 0.00020 | 2.34 |
| 214098_at | KIAA1107 | KIAA1107 | 0.00093 | 4.21 |
| 228067_at | KIAA1211-like | KIAA1211L | 0.00012 | 2.27 |
| 230765_at | KIAA1239 | KIAA1239 | 0.00004 | 7.25 |
| 236325_at | KIAA1377 | KIAA1377 | 0.00046 | 4.47 |
| 225508_at | KIAA1468 | KIAA1468 | 0.00020 | 3.29 |
| 214772_at | KIAA1549-like | KIAA1549L | 0.00030 | 4.65 |
| 235486_at | KIAA1549-like | KIAA1549L | 0.00010 | 3.77 |
| 209255_at | kelch domain containing 10 | KLHDC10 | 0.00028 | 4.93 |
| 210111_s_at | kelch domain containing 10 | KLHDC10 | 0.00069 | 1.46 |
| 212882_at | kelch-like family member 18 | KLHL18 | 0.00009 | 1.90 |
| 229310_at | kelch-like family member 29 | KLHL29 | 0.00004 | 5.99 |
| 223250_at | kelch-like family member 7 | KLHL7 | 0.00015 | 5.05 |
| 206583_at | KRAB box domain containing 4 | KRBOX4 | 0.00053 | 2.36 |
| 201505_at | laminin, beta 1 | LAMB1 | 0.00032 | 4.91 |
| 219463_at | lysosomal-associated membrane protein family, member 5 | LAMP5 | 0.00098 | 3.62 |
| 243856_at | LanC lantibiotic synthetase component C-like 3 (bacterial) | LANCL3 | 0.00019 | 6.95 |
| 231824_at | La ribonucleoprotein domain family, member 1B | LARP1B | 0.00011 | 2.71 |
| 1561691_at | long intergenic non-protein coding RNA 326 | LINC00326 | 0.00079 | 3.49 |
| 1553357_at | long intergenic non-protein coding RNA 889 | LINC00889 | 0.00009 | 4.86 |
| 228348_at | lines homolog (Drosophila) | LINS | 0.00059 | 1.40 |
| 226779_at | LMBR1 domain containing 2 | LMBRD2 | 0.00095 | 5.21 |
| 204424_s_at | LIM domain only 3 (rhombotin-like 2) | LMO3 | 0.00050 | 2.24 |
| 227155_at | LIM domain only 4 | LMO4 | 0.00053 | 2.87 |
| 229537_at | LIM domain only 4 | LMO4 | 0.00051 | 3.83 |
| 202674_s_at | LIM domain 7 | LMO7 | 0.00044 | 3.28 |
| 1556444_a_at | uncharacterized LOC100288310 | LOC100288310 | 0.00067 | 4.73 |
| 235171_at | uncharacterized LOC100505501 | LOC100505501 | 0.00031 | 3.65 |
| 222942_s_at | uncharacterized LOC100505519 /// T-cell lymphoma invasion and metastasis 2 | LOC100505519 /// TIAM2 | 0.00000 | 2.45 |
| 231431_s_at | uncharacterized LOC100505573 | LOC100505573 | 0.00046 | 1.74 |
| 221973_at | uncharacterized LOC100506076 /// uncharacterized LOC100506123 | LOC100506076 /// LOC100506123 | 0.00028 | 4.27 |
| 225916_at | uncharacterized LOC100506639 /// zinc finger protein 131 | LOC100506639 /// ZNF131 | 0.00005 | 3.13 |
| 225635_s_at | endogenous Bornavirus-like nucleoprotein 2 pseudogene | LOC100506710 | 0.00081 | 2.58 |
| 1559342_a_at | uncharacterized LOC100506948 /// small nucleolar RNA, C/D box 107 /// small nucleolar RNA, C/D box 115-13 /// small nucleolar RNA, C/D box 115-26 /// small nucleolar RNA, C/D box 115-7 /// small nucleolar RNA, C/D box 116-28 /// small nuclear ribonucleoprotein polypeptide N | LOC100506948 /// SNORD107 /// SNORD115-13 /// SNORD115-26 /// SNORD115-7 /// SNORD116-28 /// SNRPN | 0.00039 | 2.64 |
| 1559343_at | uncharacterized LOC100506948 /// small nucleolar RNA, C/D box 107 /// small nucleolar RNA, C/D box 115-13 /// small nucleolar RNA, C/D box 115-26 /// small nucleolar RNA, C/D box 115-7 /// small nucleolar RNA, C/D box 116-28 /// small nuclear ribonucleoprotein polypeptide N | LOC100506948 /// SNORD107 /// SNORD115-13 /// SNORD115-26 /// SNORD115-7 /// SNORD116-28 /// SNRPN | 0.00004 | 3.06 |
| 1559545_at | uncharacterized LOC100506948 /// small nucleolar RNA, C/D box 107 /// small nucleolar RNA, C/D box 115-13 /// small nucleolar RNA, C/D box 115-26 /// small nucleolar RNA, C/D box 115-7 /// small nucleolar RNA, C/D box 116-28 /// small nuclear ribonucleoprotein polypeptide N | LOC100506948 /// SNORD107 /// SNORD115-13 /// SNORD115-26 /// SNORD115-7 /// SNORD116-28 /// SNRPN | 0.00067 | 2.25 |
| 229890_at | uncharacterized LOC100507547 /// proline-rich transmembrane protein 1 | LOC100507547 /// PRRT1 | 0.00095 | 2.11 |
| 1558801_at | uncharacterized LOC100652772 | LOC100652772 | 0.00002 | 2.70 |
| 1558587_at | uncharacterized LOC100996385 | LOC100996385 | 0.00087 | 6.81 |
| 239952_at | uncharacterized LOC100996668 /// zinc finger E-box binding homeobox 1 | LOC100996668 /// ZEB1 | 0.00052 | 2.61 |
| 230495_at | uncharacterized LOC150568 | LOC150568 | 0.00072 | 2.53 |
| 1557113_at | uncharacterized LOC283588 | LOC283588 | 0.00041 | 3.95 |
| 236166_at | uncharacterized LOC285147 | LOC285147 | 0.00027 | 4.35 |
| 242852_at | uncharacterized LOC285147 | LOC285147 | 0.00026 | 3.77 |
| 1560692_at | uncharacterized LOC285878 | LOC285878 | 0.00038 | 3.40 |
| 225040_s_at | rcRPE /// ribulose-5-phosphate-3-epimerase | LOC729020 /// RPE | 0.00001 | 2.80 |
| 228515_at | uncharacterized LOC90784 /// polymerase (RNA) I polypeptide A, 194kDa | LOC90784 /// POLR1A | 0.00055 | 1.95 |
| 213078_x_at | lysophosphatidylcholine acyltransferase 4 | LPCAT4 | 0.00018 | 2.06 |
| 40472_at | lysophosphatidylcholine acyltransferase 4 | LPCAT4 | 0.00003 | 3.41 |
| 206953_s_at | latrophilin 2 | LPHN2 | 0.00054 | 3.03 |
| 236264_at | latrophilin 3 | LPHN3 | 0.00035 | 3.88 |
| 212276_at | lipin 1 | LPIN1 | 0.00085 | 2.29 |
| 213496_at | lipid phosphate phosphatase-related protein type 4 | LPPR4 | 0.00005 | 5.92 |
| 231397_at | lipid phosphate phosphatase-related protein type 5 | LPPR5 | 0.00000 | 5.53 |
| 230644_at | leucine rich repeat and fibronectin type III domain containing 5 | LRFN5 | 0.00012 | 2.97 |
| 35974_at | lymphoid-restricted membrane protein | LRMP | 0.00009 | 3.14 |
| 233499_at | leucine rich repeat containing 7 | LRRC7 | 0.00001 | 7.19 |
| 212978_at | leucine rich repeat containing 8 family, member B | LRRC8B | 0.00073 | 5.54 |
| 209841_s_at | leucine rich repeat neuronal 3 | LRRN3 | 0.00002 | 2.43 |
| 203534_at | LSM1 homolog, U6 small nuclear RNA associated (S. cerevisiae) | LSM1 | 0.00095 | 1.56 |
| 226826_at | LSM11, U7 small nuclear RNA associated | LSM11 | 0.00063 | 3.78 |
| 202737_s_at | LSM4 homolog, U6 small nuclear RNA associated (S. cerevisiae) | LSM4 | 0.00059 | 2.24 |
| 1556366_s_at | LY86 antisense RNA 1 | LY86-AS1 | 0.00046 | 6.41 |
| 218561_s_at | LYR motif containing 4 | LYRM4 | 0.00026 | 2.56 |
| 218437_s_at | leucine zipper transcription factor-like 1 | LZTFL1 | 0.00012 | 2.55 |
| 235278_at | MACRO domain containing 2 | MACROD2 | 0.00005 | 3.27 |
| 219894_at | MAGE-like 2 | MAGEL2 | 0.00031 | 2.30 |
| 217922_at | mannosidase, alpha, class 1A, member 2 | MAN1A2 | 0.00080 | 2.18 |
| 228262_at | MAP7 domain containing 2 | MAP7D2 | 0.00005 | 5.20 |
| 201153_s_at | muscleblind-like splicing regulator 1 | MBNL1 | 0.00096 | 1.78 |
| 230498_at | melanin-concentrating hormone receptor 1 | MCHR1 | 0.00004 | 2.83 |
| 235740_at | multiple C2 domains, transmembrane 1 | MCTP1 | 0.00095 | 5.08 |
| 227451_s_at | mitochondrial calcium uniporter regulator 1 | MCUR1 | 0.00022 | 3.15 |
| 1558077_s_at | malate dehydrogenase 1B, NAD (soluble) | MDH1B | 0.00018 | 2.72 |
| 215167_at | mediator complex subunit 14 | MED14 | 0.00054 | 2.66 |
| 221517_s_at | mediator complex subunit 17 | MED17 | 0.00050 | 3.12 |
| 212535_at | myocyte enhancer factor 2A | MEF2A | 0.00022 | 2.22 |
| 212830_at | multiple EGF-like-domains 9 | MEGF9 | 0.00083 | 3.37 |
| 213528_at | methyltransferase like 18 | METTL18 | 0.00006 | 2.40 |
| 229018_at | methyltransferase like 25 | METTL25 | 0.00038 | 1.86 |
| 222447_at | methyltransferase like 9 | METTL9 | 0.00031 | 1.93 |
| 229254_at | major facilitator superfamily domain containing 4 | MFSD4 | 0.00046 | 3.10 |
| 238862_at | major facilitator superfamily domain containing 4 | MFSD4 | 0.00015 | 6.48 |
| 242372_s_at | major facilitator superfamily domain containing 4 | MFSD4 | 0.00035 | 5.18 |
| 224598_at | mannosyl (alpha-1,3-)-glycoprotein beta-1,4-N-acetylglucosaminyltransferase, isozyme B | MGAT4B | 0.00044 | 2.05 |
| 216903_s_at | mitochondrial calcium uptake 1 | MICU1 | 0.00022 | 2.24 |
| 238458_at | mitochondrial calcium uptake family, member 3 | MICU3 | 0.00068 | 2.55 |
| 229132_at | MYC induced nuclear antigen | MINA | 0.00094 | 3.32 |
| 209585_s_at | multiple inositol-polyphosphate phosphatase 1 | MINPP1 | 0.00041 | 2.45 |
| 223522_at | microRNA 600 /// MIR600 host gene (non-protein coding) | MIR600 /// MIR600HG | 0.00063 | 1.75 |
| 209845_at | makorin ring finger protein 1 | MKRN1 | 0.00023 | 4.81 |
| 239468_at | mohawk homeobox | MKX | 0.00078 | 4.74 |
| 230122_at | myeloid/lymphoid or mixed-lineage leukemia (trithorax homolog, Drosophila); translocated to, 10 | MLLT10 | 0.00057 | 2.21 |
| 211071_s_at | myeloid/lymphoid or mixed-lineage leukemia (trithorax homolog, Drosophila); translocated to, 11 | MLLT11 | 0.00004 | 5.96 |
| 212508_at | modulator of apoptosis 1 | MOAP1 | 0.00007 | 3.51 |
| 202919_at | MOB family member 4, phocein | MOB4 | 0.00027 | 4.30 |
| 218212_s_at | molybdenum cofactor synthesis 2 | MOCS2 | 0.00032 | 2.51 |
| 227669_at | mitochondrial pyruvate carrier 2 | MPC2 | 0.00004 | 6.00 |
| 203740_at | M-phase phosphoprotein 6 | MPHOSPH6 | 0.00011 | 1.77 |
| 218586_at | MRG/MORF4L binding protein | MRGBP | 0.00020 | 1.99 |
| 218027_at | mitochondrial ribosomal protein L15 | MRPL15 | 0.00078 | 3.83 |
| 203465_at | mitochondrial ribosomal protein L19 | MRPL19 | 0.00045 | 2.41 |
| 232071_at | mitochondrial ribosomal protein L19 | MRPL19 | 0.00092 | 3.24 |
| 218339_at | mitochondrial ribosomal protein L22 | MRPL22 | 0.00079 | 2.00 |
| 209609_s_at | mitochondrial ribosomal protein L9 | MRPL9 | 0.00020 | 2.27 |
| 226296_s_at | mitochondrial ribosomal protein S15 | MRPS15 | 0.00027 | 2.20 |
| 222403_at | mitochondrial carrier 2 | MTCH2 | 0.00002 | 3.17 |
| 212248_at | metadherin | MTDH | 0.00008 | 3.05 |
| 227277_at | metadherin | MTDH | 0.00003 | 2.18 |
| 219363_s_at | MTERF domain containing 1 | MTERFD1 | 0.00000 | 2.79 |
| 203347_s_at | metal response element binding transcription factor 2 | MTF2 | 0.00006 | 3.89 |
| 216095_x_at | myotubularin related protein 1 | MTMR1 | 0.00029 | 1.86 |
| 213278_at | myotubularin related protein 9 | MTMR9 | 0.00003 | 2.80 |
| 225673_at | myeloid-associated differentiation marker | MYADM | 0.00027 | 2.17 |
| 210016_at | myelin transcription factor 1-like | MYT1L | 0.00085 | 5.34 |
| 221867_at | NEDD4 binding protein 1 | N4BP1 | 0.00055 | 1.55 |
| 225888_at | N(alpha)-acetyltransferase 25, NatB auxiliary subunit | NAA25 | 0.00050 | 2.32 |
| 220925_at | N(alpha)-acetyltransferase 35, NatC auxiliary subunit | NAA35 | 0.00062 | 2.94 |
| 217745_s_at | N(alpha)-acetyltransferase 50, NatE catalytic subunit | NAA50 | 0.00041 | 2.60 |
| 1556121_at | nucleosome assembly protein 1-like 1 | NAP1L1 | 0.00047 | 4.80 |
| 208753_s_at | nucleosome assembly protein 1-like 1 | NAP1L1 | 0.00078 | 2.55 |
| 208754_s_at | nucleosome assembly protein 1-like 1 | NAP1L1 | 0.00084 | 3.11 |
| 204749_at | nucleosome assembly protein 1-like 3 | NAP1L3 | 0.00042 | 6.43 |
| 228062_at | nucleosome assembly protein 1-like 5 | NAP1L5 | 0.00018 | 14.07 |
| 228063_s_at | nucleosome assembly protein 1-like 5 | NAP1L5 | 0.00034 | 4.12 |
| 211685_s_at | neurocalcin delta | NCALD | 0.00053 | 4.95 |
| 237002_at | neurochondrin | NCDN | 0.00083 | 1.51 |
| 225847_at | neutral cholesterol ester hydrolase 1 | NCEH1 | 0.00023 | 9.83 |
| 203315_at | NCK adaptor protein 2 | NCK2 | 0.00043 | 1.66 |
| 225344_at | nuclear receptor coactivator 7 | NCOA7 | 0.00081 | 4.43 |
| 217800_s_at | Nedd4 family interacting protein 1 | NDFIP1 | 0.00006 | 6.28 |
| 224801_at | Nedd4 family interacting protein 2 | NDFIP2 | 0.00062 | 5.28 |
| 217286_s_at | NDRG family member 3 | NDRG3 | 0.00031 | 2.32 |
| 218160_at | NADH dehydrogenase (ubiquinone) 1 alpha subcomplex, 8, 19kDa | NDUFA8 | 0.00049 | 2.85 |
| 228355_s_at | NADH dehydrogenase (ubiquinone) complex I, assembly factor 2 | NDUFAF2 | 0.00055 | 1.51 |
| 223112_s_at | NADH dehydrogenase (ubiquinone) 1 beta subcomplex, 10, 22kDa | NDUFB10 | 0.00041 | 3.09 |
| 203613_s_at | NADH dehydrogenase (ubiquinone) 1 beta subcomplex, 6, 17kDa | NDUFB6 | 0.00080 | 2.44 |
| 226616_s_at | NADH dehydrogenase (ubiquinone) flavoprotein 3, 10kDa | NDUFV3 | 0.00066 | 2.41 |
| 233305_at | N-terminal EF-hand calcium binding protein 1 | NECAB1 | 0.00039 | 4.78 |
| 243357_at | neuronal growth regulator 1 | NEGR1 | 0.00048 | 4.07 |
| 203413_at | NEL-like 2 (chicken) | NELL2 | 0.00008 | 12.76 |
| 200758_s_at | nuclear factor (erythroid-derived 2)-like 1 | NFE2L1 | 0.00075 | 1.68 |
| 223397_s_at | NIP7, nucleolar pre-rRNA processing protein | NIP7 | 0.00010 | 2.89 |
| 212129_at | non imprinted in Prader-Willi/Angelman syndrome 2 | NIPA2 | 0.00085 | 2.04 |
| 205004_at | NFKB repressing factor | NKRF | 0.00079 | 1.80 |
| 1554689_a_at | neuroligin 4, X-linked | NLGN4X | 0.00070 | 1.62 |
| 228797_at | neurolysin (metallopeptidase M3 family) | NLN | 0.00047 | 2.32 |
| 202882_x_at | nucleolar protein 7, 27kDa | NOL7 | 0.00052 | 2.01 |
| 210097_s_at | nucleolar protein 7, 27kDa | NOL7 | 0.00055 | 2.32 |
| 39549_at | neuronal PAS domain protein 2 | NPAS2 | 0.00007 | 1.93 |
| 229014_at | NR2F1 antisense RNA 1 | NR2F1-AS1 | 0.00016 | 2.37 |
| 211671_s_at | nuclear receptor subfamily 3, group C, member 1 (glucocorticoid receptor) | NR3C1 | 0.00055 | 3.94 |
| 202599_s_at | nuclear receptor interacting protein 1 | NRIP1 | 0.00015 | 4.06 |
| 201922_at | NSA2 ribosome biogenesis homolog (S. cerevisiae) | NSA2 | 0.00091 | 2.01 |
| 209570_s_at | neuron specific gene family member 1 | NSG1 | 0.00028 | 5.59 |
| 225439_at | NudC domain containing 1 | NUDCD1 | 0.00073 | 1.71 |
| 219855_at | nudix (nucleoside diphosphate linked moiety X)-type motif 11 | NUDT11 | 0.00029 | 4.42 |
| 225225_at | OIP5 antisense RNA 1 | OIP5-AS1 | 0.00055 | 4.24 |
| 1554524_a_at | olfactomedin 3 | OLFM3 | 0.00037 | 3.96 |
| 1554526_at | olfactomedin 3 | OLFM3 | 0.00096 | 5.54 |
| 214111_at | opioid binding protein/cell adhesion molecule-like | OPCML | 0.00073 | 3.01 |
| 219032_x_at | opsin 3 | OPN3 | 0.00088 | 5.35 |
| 1554706_at | olfactory receptor, family 2, subfamily L, member 13 | OR2L13 | 0.00041 | 4.64 |
| 209627_s_at | oxysterol binding protein-like 3 | OSBPL3 | 0.00022 | 2.88 |
| 236261_at | oxysterol binding protein-like 6 | OSBPL6 | 0.00010 | 2.52 |
| 218196_at | osteopetrosis associated transmembrane protein 1 | OSTM1 | 0.00002 | 3.04 |
| 202780_at | 3-oxoacid CoA transferase 1 | OXCT1 | 0.00064 | 4.62 |
| 218197_s_at | oxidation resistance 1 | OXR1 | 0.00070 | 8.11 |
| 222553_x_at | oxidation resistance 1 | OXR1 | 0.00080 | 7.25 |
| 214204_at | PARK2 co-regulated | PACRG | 0.00054 | 2.43 |
| 208051_s_at | poly(A) binding protein interacting protein 1 | PAIP1 | 0.00086 | 2.91 |
| 226843_s_at | PAP associated domain containing 5 | PAPD5 | 0.00050 | 1.49 |
| 225809_at | prostate androgen-regulated mucin-like protein 1 | PARM1 | 0.00055 | 2.70 |
| 244008_at | poly (ADP-ribose) polymerase family, member 8 | PARP8 | 0.00020 | 3.10 |
| 215972_at | prostate androgen-regulated transcript 1 (non-protein coding) | PART1 | 0.00023 | 2.40 |
| 225466_at | protein associated with topoisomerase II homolog 1 (yeast) | PATL1 | 0.00049 | 1.92 |
| 212825_at | PAX interacting (with transcription-activation domain) protein 1 | PAXIP1 | 0.00018 | 2.15 |
| 223294_at | polysaccharide biosynthesis domain containing 1 | PBDC1 | 0.00093 | 3.34 |
| 212148_at | pre-B-cell leukemia homeobox 1 | PBX1 | 0.00098 | 1.68 |
| 228640_at | protocadherin 7 | PCDH7 | 0.00000 | 9.30 |
| 210674_s_at | protocadherin alpha 1 | PCDHA1 | 0.00035 | 4.80 |
| 223435_s_at | protocadherin alpha 1 | PCDHA1 | 0.00003 | 2.70 |
| 205202_at | protein-L-isoaspartate (D-aspartate) O-methyltransferase | PCMT1 | 0.00055 | 4.32 |
| 208857_s_at | protein-L-isoaspartate (D-aspartate) O-methyltransferase | PCMT1 | 0.00002 | 6.97 |
| 229287_at | pecanex homolog (Drosophila) | PCNX | 0.00053 | 2.77 |
| 241382_at | Purkinje cell protein 4 like 1 | PCP4L1 | 0.00012 | 3.23 |
| 227751_at | programmed cell death 5 | PDCD5 | 0.00094 | 2.71 |
| 200979_at | pyruvate dehydrogenase (lipoamide) alpha 1 | PDHA1 | 0.00001 | 3.30 |
| 228959_at | pyruvate dehydrogenase kinase, isozyme 3 | PDK3 | 0.00006 | 3.45 |
| 222572_at | pyruvate dehyrogenase phosphatase catalytic subunit 1 | PDP1 | 0.00092 | 7.06 |
| 212140_at | PDS5, regulator of cohesion maintenance, homolog A (S. cerevisiae) | PDS5A | 0.00034 | 1.55 |
| 209242_at | paternally expressed 3 | PEG3 | 0.00018 | 3.43 |
| 209243_s_at | paternally expressed 3 | PEG3 | 0.00055 | 3.49 |
| 218336_at | prefoldin subunit 2 | PFDN2 | 0.00096 | 2.06 |
| 219225_at | piggyBac transposable element derived 5 | PGBD5 | 0.00014 | 2.79 |
| 229256_at | phosphoglucomutase 2-like 1 | PGM2L1 | 0.00015 | 9.93 |
| 229553_at | phosphoglucomutase 2-like 1 | PGM2L1 | 0.00058 | 4.91 |
| 238417_at | phosphoglucomutase 2-like 1 | PGM2L1 | 0.00006 | 4.29 |
| 225005_at | PHD finger protein 13 | PHF13 | 0.00062 | 2.04 |
| 212660_at | PHD finger protein 15 | PHF15 | 0.00008 | 1.99 |
| 231967_at | PHD finger protein 20-like 1 | PHF20L1 | 0.00048 | 4.66 |
| 213407_at | PH domain and leucine rich repeat protein phosphatase 2 | PHLPP2 | 0.00046 | 2.69 |
| 226623_at | phytanoyl-CoA 2-hydroxylase interacting protein-like | PHYHIPL | 0.00002 | 2.41 |
| 205273_s_at | pitrilysin metallopeptidase 1 | PITRM1 | 0.00072 | 2.40 |
| 204612_at | protein kinase (cAMP-dependent, catalytic) inhibitor alpha | PKIA | 0.00010 | 3.43 |
| 226864_at | protein kinase (cAMP-dependent, catalytic) inhibitor alpha | PKIA | 0.00023 | 5.85 |
| 235394_at | phospholipase A2-activating protein | PLAA | 0.00004 | 3.28 |
| 213309_at | phospholipase C-like 2 | PLCL2 | 0.00028 | 3.32 |
| 235230_at | phosphatidylinositol-specific phospholipase C, X domain containing 2 | PLCXD2 | 0.00073 | 5.25 |
| 219024_at | pleckstrin homology domain containing, family A (phosphoinositide binding specific) member 1 | PLEKHA1 | 0.00051 | 2.57 |
| 226247_at | pleckstrin homology domain containing, family A (phosphoinositide binding specific) member 1 | PLEKHA1 | 0.00031 | 2.78 |
| 201410_at | pleckstrin homology domain containing, family B (evectins) member 2 | PLEKHB2 | 0.00052 | 4.59 |
| 235360_at | pleckstrin homology domain containing, family M, member 3 | PLEKHM3 | 0.00082 | 3.33 |
| 209598_at | paraneoplastic Ma antigen 2 | PNMA2 | 0.00018 | 13.42 |
| 235758_at | paraneoplastic Ma antigen family member 6A /// paraneoplastic Ma antigen family member 6B /// paraneoplastic Ma antigen family member 6C /// paraneoplastic Ma antigen family member 6D | PNMA6A /// PNMA6B /// PNMA6C /// PNMA6D | 0.00007 | 1.77 |
| 218824_at | paraneoplastic Ma antigen family-like 1 | PNMAL1 | 0.00069 | 6.14 |
| 242905_at | partner of NOB1 homolog (S. cerevisiae) | PNO1 | 0.00054 | 2.65 |
| 217806_s_at | polymerase (DNA-directed), delta interacting protein 2 | POLDIP2 | 0.00042 | 1.88 |
| 209317_at | polymerase (RNA) I polypeptide C, 30kDa | POLR1C | 0.00042 | 2.58 |
| 224874_at | polymerase (RNA) I polypeptide D, 16kDa | POLR1D | 0.00035 | 1.58 |
| 1555837_s_at | polymerase (RNA) II (DNA directed) polypeptide B, 140kDa | POLR2B | 0.00077 | 2.57 |
| 219459_at | polymerase (RNA) III (DNA directed) polypeptide B | POLR3B | 0.00007 | 2.50 |
| 209482_at | processing of precursor 7, ribonuclease P/MRP subunit (S. cerevisiae) | POP7 | 0.00066 | 2.55 |
| 232181_at | peroxisome proliferator-activated receptor gamma, coactivator 1 beta | PPARGC1B | 0.00009 | 4.24 |
| 212686_at | protein phosphatase, Mg2+/Mn2+ dependent, 1H | PPM1H | 0.00076 | 4.85 |
| 229506_at | protein phosphatase, Mg2+/Mn2+ dependent, 1L | PPM1L | 0.00000 | 4.06 |
| 1553955_at | protein phosphatase 1, regulatory subunit 21 | PPP1R21 | 0.00085 | 3.15 |
| 228013_at | protein phosphatase 2, regulatory subunit B, alpha | PPP2R2A | 0.00074 | 2.12 |
| 233002_at | protein phosphatase 4, regulatory subunit 4 | PPP4R4 | 0.00009 | 9.63 |
| 225204_at | PTC7 protein phosphatase homolog (S. cerevisiae) | PPTC7 | 0.00011 | 2.03 |
| 225213_at | PTC7 protein phosphatase homolog (S. cerevisiae) | PPTC7 | 0.00091 | 6.89 |
| 205277_at | PR domain containing 2, with ZNF domain | PRDM2 | 0.00015 | 2.35 |
| 229156_s_at | PRKAG2 antisense RNA 1 | PRKAG2-AS1 | 0.00027 | 4.44 |
| 229157_at | PRKAG2 antisense RNA 1 | PRKAG2-AS1 | 0.00002 | 3.51 |
| 209323_at | protein-kinase, interferon-inducible double stranded RNA dependent inhibitor, repressor of (P58 repressor) | PRKRIR | 0.00031 | 2.37 |
| 220553_s_at | PRP39 pre-mRNA processing factor 39 homolog (S. cerevisiae) | PRPF39 | 0.00028 | 2.08 |
| 1558097_at | proline rich 14-like | PRR14L | 0.00037 | 1.86 |
| 212805_at | prune homolog 2 (Drosophila) | PRUNE2 | 0.00099 | 2.86 |
| 223363_at | proteasome (prosome, macropain) assembly chaperone 3 | PSMG3 | 0.00012 | 2.40 |
| 201433_s_at | phosphatidylserine synthase 1 | PTDSS1 | 0.00012 | 2.56 |
| 221840_at | protein tyrosine phosphatase, receptor type, E | PTPRE | 0.00038 | 2.41 |
| 203029_s_at | protein tyrosine phosphatase, receptor type, N polypeptide 2 | PTPRN2 | 0.00050 | 5.05 |
| 226571_s_at | protein tyrosine phosphatase, receptor type, S | PTPRS | 0.00016 | 1.70 |
| 205948_at | protein tyrosine phosphatase, receptor type, T | PTPRT | 0.00000 | 4.05 |
| 201166_s_at | pumilio homolog 1 (Drosophila) | PUM1 | 0.00026 | 1.82 |
| 225120_at | purine-rich element binding protein B | PURB | 0.00020 | 2.62 |
| 213325_at | poliovirus receptor-related 3 | PVRL3 | 0.00098 | 2.49 |
| 201608_s_at | PWP1 homolog (S. cerevisiae) | PWP1 | 0.00028 | 1.63 |
| 213878_at | pyridine nucleotide-disulphide oxidoreductase domain 1 | PYROXD1 | 0.00097 | 2.93 |
| 202754_at | R3H domain containing 1 | R3HDM1 | 0.00015 | 3.39 |
| 229300_at | RAB3C, member RAS oncogene family | RAB3C | 0.00009 | 8.45 |
| 203020_at | RAB GTPase activating protein 1-like | RABGAP1L | 0.00001 | 2.93 |
| 221736_at | Ral GTPase activating protein, beta subunit (non-catalytic) | RALGAPB | 0.00001 | 2.12 |
| 213280_at | RAP1 GTPase activating protein 2 | RAP1GAP2 | 0.00014 | 1.86 |
| 227036_at | RAS protein activator like 2 | RASAL2 | 0.00040 | 2.44 |
| 230563_at | RasGEF domain family, member 1A | RASGEF1A | 0.00001 | 2.07 |
| 203498_at | regulator of calcineurin 2 | RCAN2 | 0.00049 | 5.13 |
| 222605_at | REST corepressor 3 | RCOR3 | 0.00023 | 5.38 |
| 208873_s_at | receptor accessory protein 5 | REEP5 | 0.00008 | 3.31 |
| 1564031_a_at | RELT-like 2 | RELL2 | 0.00054 | 1.24 |
| 224366_s_at | RALBP1 associated Eps domain containing 1 | REPS1 | 0.00072 | 2.35 |
| 227425_at | RALBP1 associated Eps domain containing 2 | REPS2 | 0.00024 | 8.20 |
| 212646_at | raftlin, lipid raft linker 1 | RFTN1 | 0.00081 | 2.34 |
| 204337_at | regulator of G-protein signaling 4 | RGS4 | 0.00014 | 11.43 |
| 230755_at | rhomboid, veinlet-like 3 (Drosophila) | RHBDL3 | 0.00071 | 1.87 |
| 221647_s_at | resistance to inhibitors of cholinesterase 8 homolog A (C. elegans) | RIC8A | 0.00036 | 1.46 |
| 222663_at | RIO kinase 2 | RIOK2 | 0.00065 | 2.76 |
| 236771_at | ripply2 homolog (zebrafish) | RIPPLY2 | 0.00033 | 2.89 |
| 226433_at | ring finger protein 157 | RNF157 | 0.00096 | 1.67 |
| 230143_at | ring finger protein 165 | RNF165 | 0.00037 | 2.56 |
| 226766_at | roundabout, axon guidance receptor, homolog 2 (Drosophila) | ROBO2 | 0.00011 | 3.58 |
| 224738_x_at | ribosomal protein L7-like 1 | RPL7L1 | 0.00008 | 1.63 |
| 225515_s_at | ribosomal protein L7-like 1 /// WAC antisense RNA 1 (head to head) | RPL7L1 /// WAC-AS1 | 0.00024 | 1.88 |
| 1556061_at | ribonuclease P/MRP 30kDa subunit | RPP30 | 0.00095 | 2.24 |
| 228503_at | ribosomal protein S6 kinase, 90kDa, polypeptide 6 | RPS6KA6 | 0.00067 | 2.71 |
| 1554080_at | RCD1 required for cell differentiation1 homolog (S. pombe) | RQCD1 | 0.00071 | 3.21 |
| 218307_at | radical S-adenosyl methionine domain containing 1 | RSAD1 | 0.00020 | 2.03 |
| 218166_s_at | remodeling and spacing factor 1 | RSF1 | 0.00082 | 2.06 |
| 213750_at | ribosomal L1 domain containing 1 | RSL1D1 | 0.00005 | 5.08 |
| 225773_at | ring finger and SPRY domain containing 1 | RSPRY1 | 0.00074 | 1.64 |
| 230469_at | rhotekin 2 | RTKN2 | 0.00022 | 3.83 |
| 203485_at | reticulon 1 | RTN1 | 0.00064 | 1.76 |
| 210222_s_at | reticulon 1 | RTN1 | 0.00088 | 8.17 |
| 219549_s_at | reticulon 3 | RTN3 | 0.00035 | 5.84 |
| 224564_s_at | reticulon 3 | RTN3 | 0.00079 | 2.55 |
| 1554133_at | RUN and FYVE domain containing 2 | RUFY2 | 0.00038 | 2.26 |
| 213939_s_at | RUN and FYVE domain containing 3 | RUFY3 | 0.00067 | 2.08 |
| 226298_at | RUN domain containing 1 | RUNDC1 | 0.00001 | 4.41 |
| 206196_s_at | RUN domain containing 3A | RUNDC3A | 0.00003 | 3.19 |
| 241703_at | RUN domain containing 3B | RUNDC3B | 0.00093 | 2.66 |
| 202797_at | SAC1 suppressor of actin mutations 1-like (yeast) | SACM1L | 0.00065 | 2.60 |
| 241365_at | SATB homeobox 1 | SATB1 | 0.00017 | 4.77 |
| 213435_at | SATB homeobox 2 | SATB2 | 0.00075 | 5.77 |
| 211423_s_at | sterol-C5-desaturase | SC5D | 0.00092 | 2.46 |
| 216399_s_at | S-phase cyclin A-associated protein in the ER | SCAPER | 0.00083 | 2.88 |
| 237790_at | sodium channel, voltage gated, type VIII, alpha subunit | SCN8A | 0.00006 | 3.43 |
| 202061_s_at | sel-1 suppressor of lin-12-like (C. elegans) | SEL1L | 0.00051 | 2.10 |
| 202063_s_at | sel-1 suppressor of lin-12-like (C. elegans) | SEL1L | 0.00079 | 2.65 |
| 222883_at | Sel1 repeat containing 1 | SELRC1 | 0.00091 | 1.91 |
| 232183_at | serine active site containing 1 | SERAC1 | 0.00006 | 3.13 |
| 205352_at | serpin peptidase inhibitor, clade I (neuroserpin), member 1 | SERPINI1 | 0.00013 | 10.54 |
| 230660_at | SERTA domain containing 4 | SERTAD4 | 0.00054 | 3.58 |
| 227041_at | SEC14 and spectrin domains 1 | SESTD1 | 0.00034 | 1.96 |
| 40189_at | SET nuclear oncogene | SET | 0.00023 | 3.25 |
| 202775_s_at | splicing factor, suppressor of white-apricot homolog (Drosophila) | SFSWAP | 0.00073 | 1.73 |
| 223391_at | sphingosine-1-phosphate phosphatase 1 | SGPP1 | 0.00075 | 2.78 |
| 230287_at | small G protein signaling modulator 1 | SGSM1 | 0.00098 | 3.53 |
| 228745_at | small glutamine-rich tetratricopeptide repeat (TPR)-containing, beta | SGTB | 0.00032 | 4.05 |
| 225354_s_at | SH3 domain binding glutamic acid-rich protein like 2 | SH3BGRL2 | 0.00087 | 3.43 |
| 205751_at | SH3-domain GRB2-like 2 | SH3GL2 | 0.00095 | 5.74 |
| 202777_at | soc-2 suppressor of clear homolog (C. elegans) | SHOC2 | 0.00090 | 2.70 |
| 228026_at | suppressor of IKBKE 1 | SIKE1 | 0.00029 | 2.72 |
| 228805_at | SUMO-interacting motifs containing 1 | SIMC1 | 0.00096 | 3.01 |
| 238029_s_at | solute carrier family 16, member 14 (monocarboxylic acid transporter 14) | SLC16A14 | 0.00061 | 3.79 |
| 230003_at | solute carrier family 16, member 7 (monocarboxylic acid transporter 2) | SLC16A7 | 0.00014 | 5.94 |
| 214821_at | solute carrier family 25 (mitochondrial carrier; adenine nucleotide translocator), member 4 | SLC25A4 | 0.00036 | 2.99 |
| 227012_at | solute carrier family 25, member 40 | SLC25A40 | 0.00043 | 5.82 |
| 212833_at | solute carrier family 25, member 46 | SLC25A46 | 0.00090 | 3.79 |
| 226831_at | solute carrier family 25, member 46 | SLC25A46 | 0.00018 | 4.17 |
| 1557107_at | SLC26A4 antisense RNA 1 | SLC26A4-AS1 | 0.00029 | 5.38 |
| 227176_at | solute carrier family 2 (facilitated glucose transporter), member 13 | SLC2A13 | 0.00014 | 4.09 |
| 203164_at | solute carrier family 33 (acetyl-CoA transporter), member 1 | SLC33A1 | 0.00082 | 2.90 |
| 225881_at | solute carrier family 35, member B4 | SLC35B4 | 0.00084 | 2.90 |
| 1568623_a_at | solute carrier family 35, member E4 | SLC35E4 | 0.00000 | 1.94 |
| 230874_at | solute carrier family 36 (proton/amino acid symporter), member 4 | SLC36A4 | 0.00086 | 5.23 |
| 223304_at | solute carrier family 37 (glycerol-3-phosphate transporter), member 3 | SLC37A3 | 0.00040 | 1.72 |
| 235763_at | solute carrier family 44, member 5 | SLC44A5 | 0.00009 | 3.93 |
| 1558201_s_at | solute carrier family 4 (anion exchanger), member 1, adaptor protein | SLC4A1AP | 0.00041 | 4.79 |
| 228175_at | solute carrier family 4, sodium bicarbonate cotransporter, member 8 | SLC4A8 | 0.00099 | 1.97 |
| 239352_at | solute carrier family 6 (neutral amino acid transporter), member 15 | SLC6A15 | 0.00008 | 3.67 |
| 226550_at | solute carrier family 9, subfamily A (NHE7, cation proton antiporter 7), member 7 | SLC9A7 | 0.00025 | 2.45 |
| 230680_at | SLIT and NTRK-like family, member 4 | SLITRK4 | 0.00061 | 6.70 |
| 227990_at | SLU7 splicing factor homolog (S. cerevisiae) | SLU7 | 0.00023 | 6.18 |
| 218137_s_at | small ArfGAP 1 | SMAP1 | 0.00012 | 3.27 |
| 225282_at | small ArfGAP2 | SMAP2 | 0.00025 | 2.72 |
| 219806_s_at | single-pass membrane protein with coiled-coil domains 4 | SMCO4 | 0.00067 | 3.20 |
| 227124_at | small integral membrane protein 13 | SMIM13 | 0.00048 | 4.40 |
| 219772_s_at | small muscle protein, X-linked | SMPX | 0.00094 | 3.42 |
| 233946_at | smu-1 suppressor of mec-8 and unc-52 homolog (C. elegans) | SMU1 | 0.00030 | 3.46 |
| 212922_s_at | SET and MYND domain containing 2 | SMYD2 | 0.00077 | 4.02 |
| 204001_at | small nuclear RNA activating complex, polypeptide 3, 50kDa | SNAPC3 | 0.00039 | 3.16 |
| 213203_at | small nuclear RNA activating complex, polypeptide 5, 19kDa | SNAPC5 | 0.00055 | 2.83 |
| 236081_at | synuclein, alpha (non A4 component of amyloid precursor) | SNCA | 0.00006 | 12.35 |
| 218032_at | stannin | SNN | 0.00025 | 2.46 |
| 218033_s_at | stannin | SNN | 0.00008 | 1.94 |
| 212440_at | small nuclear ribonucleoprotein 27kDa (U4/U6.U5) | SNRNP27 | 0.00065 | 2.06 |
| 213358_at | SOGA family member 2 | SOGA2 | 0.00009 | 4.78 |
| 212560_at | sortilin-related receptor, L(DLR class) A repeats containing | SORL1 | 0.00062 | 3.13 |
| 228509_at | SPHK1 interactor, AKAP domain containing | SPHKAP | 0.00009 | 3.51 |
| 204675_at | steroid-5-alpha-reductase, alpha polypeptide 1 (3-oxo-5 alpha-steroid delta 4-dehydrogenase alpha 1) | SRD5A1 | 0.00087 | 9.21 |
| 211056_s_at | steroid-5-alpha-reductase, alpha polypeptide 1 (3-oxo-5 alpha-steroid delta 4-dehydrogenase alpha 1) | SRD5A1 | 0.00004 | 2.47 |
| 235611_at | splicing regulatory glutamine/lysine-rich protein 1 | SREK1 | 0.00084 | 3.50 |
| 230010_at | serine/arginine repetitive matrix 4 | SRRM4 | 0.00001 | 2.73 |
| 213140_s_at | synovial sarcoma translocation gene on chromosome 18-like 1 | SS18L1 | 0.00089 | 3.40 |
| 222482_at | single stranded DNA binding protein 3 | SSBP3 | 0.00003 | 3.24 |
| 223051_at | SSU72 RNA polymerase II CTD phosphatase homolog (S. cerevisiae) | SSU72 | 0.00055 | 2.69 |
| 203018_s_at | synovial sarcoma, X breakpoint 2 interacting protein | SSX2IP | 0.00030 | 5.05 |
| 210871_x_at | synovial sarcoma, X breakpoint 2 interacting protein | SSX2IP | 0.00041 | 3.28 |
| 220979_s_at | ST6 (alpha-N-acetyl-neuraminyl-2,3-beta-galactosyl-1,3)-N-acetylgalactosaminide alpha-2,6-sialyltransferase 5 | ST6GALNAC5 | 0.00041 | 6.40 |
| 207871_s_at | suppression of tumorigenicity 7 /// ST7 overlapping transcript 3 (non-protein coding) | ST7 /// ST7-OT3 | 0.00051 | 2.99 |
| 230262_at | ST8 alpha-N-acetyl-neuraminide alpha-2,8-sialyltransferase 3 | ST8SIA3 | 0.00064 | 2.95 |
| 226390_at | StAR-related lipid transfer (START) domain containing 4 | STARD4 | 0.00004 | 2.62 |
| 203000_at | stathmin-like 2 | STMN2 | 0.00020 | 7.74 |
| 203001_s_at | stathmin-like 2 | STMN2 | 0.00007 | 9.54 |
| 222557_at | stathmin-like 3 | STMN3 | 0.00005 | 1.88 |
| 226822_at | storkhead box 2 | STOX2 | 0.00099 | 3.17 |
| 242989_at | striatin, calmodulin binding protein | STRN | 0.00037 | 2.61 |
| 203767_s_at | steroid sulfatase (microsomal), isozyme S | STS | 0.00064 | 8.62 |
| 235227_at | syntaxin binding protein 5 (tomosyn) | STXBP5 | 0.00001 | 4.51 |
| 213505_s_at | SURP and G patch domain containing 2 | SUGP2 | 0.00077 | 1.56 |
| 214092_x_at | SURP and G patch domain containing 2 | SUGP2 | 0.00034 | 1.60 |
| 219425_at | sulfotransferase family 4A, member 1 | SULT4A1 | 0.00033 | 2.40 |
| 219389_at | sushi domain containing 4 | SUSD4 | 0.00013 | 3.55 |
| 214954_at | sushi domain containing 5 | SUSD5 | 0.00092 | 2.84 |
| 226086_at | synaptotagmin XIII | SYT13 | 0.00005 | 4.97 |
| 227357_at | TGF-beta activated kinase 1/MAP3K7 binding protein 3 | TAB3 | 0.00005 | 4.67 |
| 225455_at | transcriptional adaptor 1 | TADA1 | 0.00026 | 4.20 |
| 235268_at | transcriptional adaptor 2A | TADA2A | 0.00055 | 4.94 |
| 204743_at | transgelin 3 | TAGLN3 | 0.00043 | 3.54 |
| 224952_at | tetratricopeptide repeat, ankyrin repeat and coiled-coil containing 2 | TANC2 | 0.00041 | 5.72 |
| 209451_at | TRAF family member-associated NFKB activator | TANK | 0.00064 | 2.18 |
| 236637_at | taste receptor, type 2, member 14 | TAS2R14 | 0.00052 | 1.94 |
| 218268_at | TBC1 domain family, member 15 | TBC1D15 | 0.00017 | 1.98 |
| 226409_at | TBC1 domain family, member 20 | TBC1D20 | 0.00034 | 1.63 |
| 227908_at | TBC1 domain family, member 24 | TBC1D24 | 0.00026 | 2.82 |
| 213912_at | TBC1 domain family, member 30 | TBC1D30 | 0.00000 | 6.76 |
| 212956_at | TBC1 domain family, member 9 (with GRAM domain) | TBC1D9 | 0.00030 | 11.86 |
| 235890_at | transducin (beta)-like 1 X-linked receptor 1 | TBL1XR1 | 0.00020 | 3.59 |
| 208398_s_at | TBP-like 1 | TBPL1 | 0.00008 | 3.11 |
| 227705_at | transcription elongation factor A (SII)-like 7 | TCEAL7 | 0.00090 | 6.50 |
| 224819_at | transcription elongation factor A (SII)-like 8 | TCEAL8 | 0.00079 | 2.26 |
| 231257_at | transcription elongation regulator 1-like | TCERG1L | 0.00015 | 3.74 |
| 225840_at | thyrotrophic embryonic factor | TEF | 0.00020 | 1.51 |
| 213346_at | testis expressed 30 | TEX30 | 0.00061 | 1.73 |
| 221235_s_at | transforming growth factor, beta receptor associated protein 1 | TGFBRAP1 | 0.00008 | 2.29 |
| 219596_at | THAP domain containing 10 | THAP10 | 0.00050 | 3.73 |
| 229657_at | thyroid hormone receptor, beta | THRB | 0.00028 | 3.71 |
| 219248_at | THUMP domain containing 2 | THUMPD2 | 0.00081 | 1.90 |
| 208850_s_at | Thy-1 cell surface antigen | THY1 | 0.00002 | 1.81 |
| 208851_s_at | Thy-1 cell surface antigen | THY1 | 0.00002 | 1.92 |
| 213869_x_at | Thy-1 cell surface antigen | THY1 | 0.00003 | 2.03 |
| 238793_at | tigger transposable element derived 7 | TIGD7 | 0.00009 | 2.53 |
| 1552426_a_at | TM2 domain containing 3 | TM2D3 | 0.00035 | 3.80 |
| 221702_s_at | TM2 domain containing 3 | TM2D3 | 0.00075 | 3.87 |
| 201078_at | transmembrane 9 superfamily member 2 | TM9SF2 | 0.00054 | 3.81 |
| 213351_s_at | transmembrane and coiled-coil domain family 1 | TMCC1 | 0.00054 | 1.90 |
| 227112_at | transmembrane and coiled-coil domain family 1 | TMCC1 | 0.00037 | 2.36 |
| 225343_at | transmembrane emp24 protein transport domain containing 8 | TMED8 | 0.00071 | 1.65 |
| 226529_at | transmembrane protein 106B | TMEM106B | 0.00032 | 1.97 |
| 223523_at | transmembrane protein 108 | TMEM108 | 0.00062 | 2.86 |
| 221622_s_at | transmembrane protein 126B | TMEM126B | 0.00065 | 4.71 |
| 236468_at | transmembrane protein 132B | TMEM132B | 0.00056 | 2.92 |
| 236377_at | transmembrane protein 132D | TMEM132D | 0.00027 | 5.13 |
| 222209_s_at | transmembrane protein 135 | TMEM135 | 0.00069 | 2.34 |
| 218477_at | transmembrane protein 14A | TMEM14A | 0.00018 | 4.60 |
| 230137_at | transmembrane protein 155 | TMEM155 | 0.00005 | 6.80 |
| 213338_at | transmembrane protein 158 (gene/pseudogene) | TMEM158 | 0.00004 | 2.01 |
| 235798_at | transmembrane protein 170B | TMEM170B | 0.00070 | 5.63 |
| 212164_at | transmembrane protein 183A /// transmembrane protein 183B | TMEM183A /// TMEM183B | 0.00036 | 3.61 |
| 1555790_a_at | transmembrane protein 192 /// zinc finger protein 320 | TMEM192 /// ZNF320 | 0.00092 | 2.73 |
| 225052_at | transmembrane protein 203 | TMEM203 | 0.00085 | 2.57 |
| 239776_at | transmembrane protein 232 | TMEM232 | 0.00053 | 4.30 |
| 204215_at | transmembrane protein 243, mitochondrial | TMEM243 | 0.00001 | 3.86 |
| 223006_s_at | transmembrane protein 245 | TMEM245 | 0.00025 | 2.91 |
| 223008_s_at | transmembrane protein 245 | TMEM245 | 0.00019 | 2.72 |
| 213386_at | transmembrane protein 246 | TMEM246 | 0.00049 | 2.81 |
| 224458_at | transmembrane protein 246 | TMEM246 | 0.00037 | 2.26 |
| 226647_at | transmembrane protein 25 | TMEM25 | 0.00007 | 1.85 |
| 217743_s_at | transmembrane protein 30A | TMEM30A | 0.00011 | 5.20 |
| 217766_s_at | transmembrane protein 50A | TMEM50A | 0.00007 | 2.08 |
| 219449_s_at | transmembrane protein 70 | TMEM70 | 0.00047 | 6.42 |
| 227420_at | tumor necrosis factor, alpha-induced protein 8-like 1 | TNFAIP8L1 | 0.00014 | 3.61 |
| 218856_at | tumor necrosis factor receptor superfamily, member 21 | TNFRSF21 | 0.00035 | 3.10 |
| 207305_s_at | trafficking protein particle complex 8 | TRAPPC8 | 0.00022 | 2.50 |
| 1560698_a_at | TRHDE antisense RNA 1 | TRHDE-AS1 | 0.00002 | 1.53 |
| 213009_s_at | tripartite motif containing 37 | TRIM37 | 0.00010 | 4.59 |
| 217760_at | tripartite motif containing 44 | TRIM44 | 0.00045 | 2.32 |
| 230280_at | tripartite motif containing 9 | TRIM9 | 0.00008 | 5.43 |
| 225599_s_at | triple QxxK/R motif containing | TRIQK | 0.00058 | 2.68 |
| 223267_at | tRNA methyltransferase 10 homolog C (S. cerevisiae) | TRMT10C | 0.00048 | 3.81 |
| 223282_at | teashirt zinc finger homeobox 1 | TSHZ1 | 0.00011 | 3.32 |
| 217979_at | tetraspanin 13 | TSPAN13 | 0.00097 | 3.27 |
| 225387_at | tetraspanin 5 | TSPAN5 | 0.00018 | 3.97 |
| 1560648_s_at | TSPY-like 1 | TSPYL1 | 0.00017 | 8.94 |
| 221493_at | TSPY-like 1 | TSPYL1 | 0.00010 | 4.51 |
| 212928_at | TSPY-like 4 | TSPYL4 | 0.00061 | 4.44 |
| 213122_at | TSPY-like 5 | TSPYL5 | 0.00034 | 3.59 |
| 201434_at | tetratricopeptide repeat domain 1 | TTC1 | 0.00094 | 2.34 |
| 208073_x_at | tetratricopeptide repeat domain 3 /// tetratricopeptide repeat domain 3 pseudogene 1 | TTC3 /// TTC3P1 | 0.00006 | 1.92 |
| 210645_s_at | tetratricopeptide repeat domain 3 /// tetratricopeptide repeat domain 3 pseudogene 1 | TTC3 /// TTC3P1 | 0.00063 | 2.48 |
| 219421_at | tetratricopeptide repeat domain 33 | TTC33 | 0.00093 | 2.50 |
| 226152_at | tetratricopeptide repeat domain 7B | TTC7B | 0.00001 | 3.10 |
| 213172_at | tetratricopeptide repeat domain 9 | TTC9 | 0.00025 | 4.07 |
| 213423_x_at | tumor suppressor candidate 3 | TUSC3 | 0.00069 | 4.39 |
| 218446_s_at | trans-golgi network vesicle protein 23 homolog B (S. cerevisiae) | TVP23B | 0.00025 | 2.30 |
| 216609_at | thioredoxin | TXN | 0.00034 | 2.46 |
| 226747_at | thioredoxin domain containing 16 | TXNDC16 | 0.00009 | 3.87 |
| 201588_at | thioredoxin-like 1 | TXNL1 | 0.00008 | 2.06 |
| 46270_at | ubiquitin associated protein 1 | UBAP1 | 0.00024 | 2.05 |
| 241887_at | ubiquitin-conjugating enzyme E2W (putative) | UBE2W | 0.00002 | 3.35 |
| 227413_at | ubiquitin-like domain containing CTD phosphatase 1 | UBLCP1 | 0.00017 | 3.95 |
| 222990_at | ubiquilin 1 | UBQLN1 | 0.00049 | 4.24 |
| 222991_s_at | ubiquilin 1 | UBQLN1 | 0.00041 | 2.92 |
| 239888_at | NULL | UBQLN2 | 0.00036 | 3.03 |
| 224967_at | UDP-glucose ceramide glucosyltransferase | UGCG | 0.00042 | 4.36 |
| 238542_at | UL16 binding protein 2 | ULBP2 | 0.00017 | 3.52 |
| 217935_s_at | ubiquinol-cytochrome c reductase complex chaperone | UQCC | 0.00045 | 2.19 |
| 244293_at | ubiquinol-cytochrome c reductase binding protein | UQCRB | 0.00022 | 2.41 |
| 219675_s_at | UDP-glucuronate decarboxylase 1 | UXS1 | 0.00019 | 1.79 |
| 213480_at | vesicle-associated membrane protein 4 | VAMP4 | 0.00083 | 1.99 |
| 226415_at | vesicle amine transport protein 1 homolog (T. californica)-like | VAT1L | 0.00075 | 2.96 |
| 213686_at | vacuolar protein sorting 13 homolog A (S. cerevisiae) | VPS13A | 0.00045 | 3.31 |
| 212326_at | vacuolar protein sorting 13 homolog D (S. cerevisiae) | VPS13D | 0.00051 | 2.19 |
| 203797_at | visinin-like 1 | VSNL1 | 0.00024 | 6.98 |
| 203798_s_at | visinin-like 1 | VSNL1 | 0.00070 | 6.87 |
| 230117_at | V-set and transmembrane domain containing 2A | VSTM2A | 0.00021 | 12.55 |
| 217742_s_at | WW domain containing adaptor with coiled-coil | WAC | 0.00100 | 2.35 |
| 230154_at | WW domain containing adaptor with coiled-coil | WAC | 0.00009 | 2.80 |
| 203855_at | WD repeat domain 47 | WDR47 | 0.00094 | 4.63 |
| 221531_at | WD repeat domain 61 | WDR61 | 0.00069 | 2.51 |
| 214061_at | WD repeat domain 67 | WDR67 | 0.00005 | 2.48 |
| 209053_s_at | Wolf-Hirschhorn syndrome candidate 1 | WHSC1 | 0.00083 | 2.19 |
| 222778_s_at | Wolf-Hirschhorn syndrome candidate 1 | WHSC1 | 0.00031 | 3.00 |
| 224076_s_at | Wolf-Hirschhorn syndrome candidate 1-like 1 | WHSC1L1 | 0.00036 | 1.86 |
| 229849_at | WAS/WASL interacting protein family, member 3 | WIPF3 | 0.00010 | 2.73 |
| 206698_at | X-linked Kx blood group (McLeod syndrome) | XK | 0.00045 | 4.89 |
| 217783_s_at | yippee-like 5 (Drosophila) | YPEL5 | 0.00060 | 3.41 |
| 222408_s_at | yippee-like 5 (Drosophila) | YPEL5 | 0.00076 | 1.79 |
| 218647_s_at | yrdC domain containing (E. coli) | YRDC | 0.00085 | 1.76 |
| 227049_at | zinc binding alcohol dehydrogenase domain containing 2 | ZADH2 | 0.00030 | 1.98 |
| 204847_at | zinc finger and BTB domain containing 11 | ZBTB11 | 0.00033 | 4.62 |
| 205308_at | zinc finger, C2HC-type containing 1A | ZC2HC1A | 0.00018 | 1.97 |
| 213063_at | zinc finger CCCH-type containing 14 | ZC3H14 | 0.00065 | 2.31 |
| 212655_at | zinc finger, CCHC domain containing 14 | ZCCHC14 | 0.00061 | 1.85 |
| 225538_at | zinc finger, CCHC domain containing 9 | ZCCHC9 | 0.00034 | 2.33 |
| 229240_at | zinc finger, DHHC-type containing 21 | ZDHHC21 | 0.00019 | 2.92 |
| 226650_at | zinc finger, AN1-type domain 2A | ZFAND2A | 0.00040 | 2.61 |
| 226807_at | ZFP1 zinc finger protein | ZFP1 | 0.00065 | 2.70 |
| 219929_s_at | zinc finger, FYVE domain containing 21 | ZFYVE21 | 0.00013 | 2.31 |
| 223214_s_at | zinc fingers and homeoboxes 1 | ZHX1 | 0.00063 | 2.94 |
| 225221_at | zinc finger with KRAB and SCAN domains 1 | ZKSCAN1 | 0.00093 | 1.94 |
| 228315_at | zinc finger, matrin-type 3 | ZMAT3 | 0.00072 | 2.94 |
| 202051_s_at | zinc finger, MYM-type 4 | ZMYM4 | 0.00072 | 2.50 |
| 217781_s_at | zinc finger protein 106 | ZNF106 | 0.00048 | 2.14 |
| 213452_at | zinc finger protein 184 | ZNF184 | 0.00029 | 4.69 |
| 214823_at | zinc finger protein 204, pseudogene | ZNF204P | 0.00029 | 10.51 |
| 200829_x_at | zinc finger protein 207 | ZNF207 | 0.00055 | 1.60 |
| 220497_at | zinc finger protein 214 | ZNF214 | 0.00089 | 2.02 |
| 230063_at | zinc finger protein 264 | ZNF264 | 0.00059 | 1.84 |
| 229360_at | zinc finger protein 280B | ZNF280B | 0.00046 | 2.27 |
| 230789_at | zinc finger protein 280B | ZNF280B | 0.00092 | 3.38 |
| 232014_at | zinc finger protein 30 | ZNF30 | 0.00096 | 2.49 |
| 218490_s_at | zinc finger protein 302 | ZNF302 | 0.00086 | 2.60 |
| 203521_s_at | zinc finger protein 318 | ZNF318 | 0.00040 | 2.35 |
| 219765_at | zinc finger protein 329 | ZNF329 | 0.00059 | 3.19 |
| 206448_at | zinc finger protein 365 | ZNF365 | 0.00073 | 5.23 |
| 202010_s_at | zinc finger protein 410 | ZNF410 | 0.00007 | 1.65 |
| 209944_at | zinc finger protein 410 | ZNF410 | 0.00048 | 1.52 |
| 226909_at | zinc finger protein 518B | ZNF518B | 0.00007 | 2.63 |
| 231940_at | zinc finger protein 529 | ZNF529 | 0.00039 | 3.97 |
| 1553696_s_at | zinc finger protein 569 | ZNF569 | 0.00009 | 2.88 |
| 241827_at | zinc finger protein 615 | ZNF615 | 0.00031 | 1.92 |
| 222623_s_at | zinc finger protein 639 | ZNF639 | 0.00054 | 3.53 |
| 225945_at | zinc finger protein 655 | ZNF655 | 0.00041 | 4.04 |
| 227080_at | zinc finger protein 697 | ZNF697 | 0.00002 | 2.28 |
| 228988_at | zinc finger protein 711 | ZNF711 | 0.00016 | 3.81 |
| 228652_at | zinc finger protein 776 | ZNF776 | 0.00009 | 2.08 |
| 228330_at | zinc finger with UFM1-specific peptidase domain | ZUFSP | 0.00029 | 2.50 |
| 222606_at | zwilch kinetochore protein | ZWILCH | 0.00029 | 4.08 |
| 228005_at | zinc finger, X-linked, duplicated B | ZXDB | 0.00047 | 3.70 |
| 1554108_at | --- | --- | 0.00013 | 3.21 |
| 1556194_a_at | --- | --- | 0.00007 | 5.16 |
| 1556261_a_at | --- | --- | 0.00029 | 2.20 |
| 1557667_at | --- | --- | 0.00092 | 3.20 |
| 1558445_at | --- | --- | 0.00010 | 4.01 |
| 1559745_at | --- | --- | 0.00062 | 1.40 |
| 1559939_at | --- | --- | 0.00050 | 1.92 |
| 1561242_at | --- | --- | 0.00000 | 9.81 |
| 212847_at | --- | --- | 0.00040 | 2.78 |
| 213484_at | --- | --- | 0.00001 | 2.03 |
| 214949_at | --- | --- | 0.00034 | 5.91 |
| 215423_at | --- | --- | 0.00054 | 2.83 |
| 215441_at | --- | --- | 0.00015 | 2.34 |
| 217651_at | --- | --- | 0.00079 | 4.21 |
| 226883_at | --- | --- | 0.00042 | 3.21 |
| 227417_at | --- | --- | 0.00050 | 3.09 |
| 228045_at | --- | --- | 0.00033 | 2.79 |
| 228694_at | --- | --- | 0.00006 | 2.48 |
| 228835_at | --- | --- | 0.00002 | 4.65 |
| 229108_at | --- | --- | 0.00003 | 3.45 |
| 229201_at | --- | --- | 0.00052 | 7.19 |
| 229202_at | --- | --- | 0.00057 | 2.19 |
| 229243_at | --- | --- | 0.00052 | 3.18 |
| 229810_at | --- | --- | 0.00036 | 5.23 |
| 231608_at | --- | --- | 0.00011 | 1.79 |
| 233185_at | --- | --- | 0.00084 | 2.85 |
| 235655_at | --- | --- | 0.00016 | 3.35 |
| 236194_at | --- | --- | 0.00049 | 3.40 |
| 236202_at | --- | --- | 0.00056 | 3.05 |
| 236453_at | --- | --- | 0.00050 | 4.61 |
| 236714_at | --- | --- | 0.00008 | 9.19 |
| 237476_at | --- | --- | 0.00023 | 1.75 |
| 238728_at | --- | --- | 0.00096 | 2.87 |
| 241417_at | --- | --- | 0.00082 | 3.07 |
| 241484_x_at | --- | --- | 0.00041 | 3.04 |
| 241758_at | --- | --- | 0.00055 | 4.65 |
| 241863_x_at | --- | --- | 0.00011 | 4.11 |
| 242096_at | --- | --- | 0.00060 | 3.06 |
| 242300_at | --- | --- | 0.00001 | 5.42 |
| 242481_at | --- | --- | 0.00034 | 5.60 |
| 242610_x_at | --- | --- | 0.00026 | 3.32 |
| 243163_at | --- | --- | 0.00052 | 6.49 |
| 243339_at | --- | --- | 0.00041 | 5.58 |
| 243484_x_at | --- | --- | 0.00017 | 2.99 |
| 243932_at | --- | --- | 0.00030 | 3.74 |
| 244873_s_at | --- | --- | 0.00022 | 6.01 |
