# Supplementary material for: Neuronal DNA damage response‐associated dysregulation of signalling pathways and cholesterol metabolism at the earliest stages of Alzheimer‐type pathology
Source: Neuropathol Appl Neurobiol. 2015 Jul 7;42(2):167–79. doi: 10.1111/nan.12252 (PMC5102584; doi:10.1111/nan.12252)
Supplement: Supplementary file 2 — Table S2. Down‐regulated genes in high versus low neuronal DDR cases at low Braak and Braak stages (P < 0.001). [file NAN-42-167-s002.docx]

**Supplementary Table 2.** Down-regulated genes in high versus low neuronal DDR cases at low Braak and Braak stages (p<0.001).

| **Probe set ID** | | **Gene** | **Gene Symbol** | ***p-value*** | **FC** |
| --- | --- | --- | --- | --- | --- |
| *Insulin signalling* |  | |  |  |  |
| 1552616_a_at | acetyl-CoA carboxylase beta | | ACACB | 0.00060 | -1.55 |
| 210019_at | calmodulin-like 3 | | CALML3 | 0.00047 | -1.45 |
| 221539_at | eukaryotic translation initiation factor 4E binding protein 1 | | EIF4EBP1 | 0.00049 | -1.40 |
| 215413_at | exocyst complex component 7 | | EXOC7 | 0.00019 | -1.72 |
| 241665_x_at | forkhead box O1 | | FOXO1 | 0.00029 | -1.64 |
| 241904_at | LIPE antisense RNA 1 | | LIPE-AS1 | 0.00005 | -2.02 |
| 211578_s_at | ribosomal protein S6 kinase, 70kDa, polypeptide 1 | | RPS6KB1 | 0.00099 | -1.28 |
| 210001_s_at | suppressor of cytokine signaling 1 | | SOCS1 | 0.00040 | -1.69 |
| 213337_s_at | suppressor of cytokine signaling 1 | | SOCS1 | 0.00033 | -1.80 |
| 238830_at | Son of sevenless homolog 2 (Drosophila) | | SOS2 | 0.00060 | -1.76 |
| 1558875_at | sterol regulatory element binding transcription factor 1 | | SREBF1 | 0.00036 | -1.46 |
|  |  | |  |  |  |
| *p53 signalling* |  | |  |  |  |
| 206083_at | brain-specific angiogenesis inhibitor 1 | | BAI1 | 0.00094 | -1.69 |
| 207039_at | cyclin-dependent kinase inhibitor 2A | | CDKN2A | 0.00040 | -1.80 |
| 217744_s_at | PERP, TP53 apoptosis effector | | PERP | 0.00051 | -2.25 |
| 235086_at | thrombospondin 1 | | THBS1 | 0.00100 | -1.44 |
| 209295_at | tumor necrosis factor receptor superfamily, member 10b | | TNFRSF10B | 0.00089 | -1.89 |
| 201746_at | tumor protein p53 | | TP53 | 0.00026 | -1.67 |
|  |  | |  |  |  |
| *Apoptosis* |  | |  |  |  |
| 220578_at | ADAMTS-like 4 /// uncharacterized LOC100996516 | | ADAMTSL4 /// LOC100996516 | 0.00090 | -1.56 |
| 211277_x_at | amyloid beta (A4) precursor protein | | APP | 0.00006 | -1.61 |
| 203728_at | BCL2-antagonist/killer 1 | | BAK1 | 0.00094 | -1.50 |
| 223514_at | caspase recruitment domain family, member 11 | | CARD11 | 0.00012 | -1.62 |
| 224113_at | caspase recruitment domain family, member 14 | | CARD14 | 0.00020 | -1.37 |
| 206450_at | dopamine beta-hydroxylase (dopamine beta-monooxygenase) | | DBH | 0.00043 | -1.44 |
| 214372_x_at | endoplasmic reticulum to nucleus signaling 2 | | ERN2 | 0.00018 | -1.84 |
| 211118_x_at | estrogen receptor 2 (ER beta) | | ESR2 | 0.00026 | -1.71 |
| 202676_x_at | Fas-activated serine/threonine kinase | | FASTK | 0.00003 | -1.57 |
| 216224_s_at | histone deacetylase 6 | | HDAC6 | 0.00061 | -1.65 |
| 219028_at | homeodomain interacting protein kinase 2 | | HIPK2 | 0.00059 | -2.51 |
| 201008_s_at | thioredoxin-interacting protein-like /// thioredoxin interacting protein | | LOC101060503 /// TXNIP | 0.00001 | -2.49 |
| 220464_at | MCF.2 cell line derived transforming sequence-like | | MCF2L | 0.00021 | -1.45 |
| 220641_at | NADPH oxidase, EF-hand calcium binding domain 5 | | NOX5 | 0.00003 | -1.61 |
| 202340_x_at | nuclear receptor subfamily 4, group A, member 1 | | NR4A1 | 0.00032 | -1.59 |
| 214203_s_at | proline dehydrogenase (oxidase) 1 | | PRODH | 0.00056 | -2.08 |
| 220691_at | sphingomyelin synthase 1 | | SGMS1 | 0.00050 | -1.57 |
| 202935_s_at | SRY (sex determining region Y)-box 9 | | SOX9 | 0.00001 | -3.06 |
| 206056_x_at | sialophorin | | SPN | 0.00028 | -1.88 |
| 1554564_a_at | signal peptide peptidase like 3 | | SPPL3 | 0.00078 | -2.02 |
| 225544_at | T-box 3 | | TBX3 | 0.00006 | -2.28 |
| 229565_x_at | T-box 3 | | TBX3 | 0.00099 | -1.58 |
| 240715_at | T-box 5 | | TBX5 | 0.00031 | -2.79 |
| 212084_at | testis expressed 261 | | TEX261 | 0.00013 | -1.69 |
| 227345_at | tumor necrosis factor receptor superfamily, member 10d, decoy with truncated death domain | | TNFRSF10D | 0.00056 | -1.56 |
| 237532_at | tumor necrosis factor receptor superfamily, member 8 | | TNFRSF8 | 0.00028 | -1.52 |
| 207536_s_at | tumor necrosis factor receptor superfamily, member 9 | | TNFRSF9 | 0.00015 | -1.41 |
| 207598_x_at | X-ray repair complementing defective repair in Chinese hamster cells 2 | | XRCC2 | 0.00044 | -2.04 |
|  |  | |  |  |  |
| *Lipid transport* |  | |  |  |  |
| 204450_x_at | apolipoprotein A-I | | APOA1 | 0.00034 | -1.42 |
| 239268_at | NADH dehydrogenase (ubiquinone) Fe-S protein 1, 75kDa (NADH-coenzyme Q reductase) | | NDUFS1 | 0.00023 | -1.72 |
| 221237_s_at | oxysterol binding protein 2 | | OSBP2 | 0.00043 | -1.43 |
| 241467_at | oxysterol binding protein-like 5 | | OSBPL5 | 0.00022 | -1.82 |
| 208163_s_at | oxysterol binding protein-like 7 | | OSBPL7 | 0.00016 | -1.62 |
| 226728_at | solute carrier family 27 (fatty acid transporter), member 1 | | SLC27A1 | 0.00010 | -1.74 |
|  |  | |  |  |  |
| *Cytoskeleton* |  | |  |  |  |
| 238939_at | adducin 1 (alpha) | | ADD1 | 0.00024 | -1.90 |
| 216123_x_at | capping protein (actin filament) muscle Z-line, alpha 1 | | CAPZA1 | 0.00087 | -1.39 |
| 238388_x_at | capping protein (actin filament) muscle Z-line, alpha 1 | | CAPZB | 0.00018 | -1.57 |
| 213426_s_at | caveolin 2 | | CAV2 | 0.00071 | -1.65 |
| 205341_at | EH-domain containing 2 | | EHD2 | 0.00026 | -1.48 |
| 215200_x_at | hypothetical protein LOC100129652; ezrin | | EZR | 0.00098 | -1.58 |
| 204789_at | formin-like 1 | | FMNL1 | 0.00050 | -1.53 |
| 220071_x_at | HAUS augmin-like complex, subunit 2 | | HAUS2 | 0.00056 | -2.35 |
| 232665_x_at | HAUS augmin-like complex, subunit 6 | | HAUS6 | 0.00019 | -1.48 |
| 232906_at | kelch-like 20 (Drosophila) | | KLHL20 | 0.00049 | -1.95 |
| 232168_x_at | microtubule-actin crosslinking factor 1 | | MACF1 | 0.00001 | -1.91 |
| 204889_s_at | neuralized homolog (Drosophila) | | NEURL | 0.00073 | -1.74 |
| 238618_at | neurofibromin 2 (merlin) | | NF2 | 0.00078 | -1.45 |
| 210767_at | neurofibromin 2 (merlin) | | NF2 | 0.00054 | -1.37 |
| 203859_s_at | paralemmin | | PALM | 0.00040 | -1.36 |
| 235627_at | profilin family, member 4 | | PFN4 | 0.00005 | -1.88 |
| 211253_x_at | peptide YY | | PYY | 0.00082 | -1.54 |
| 220483_s_at | ring finger protein 19A, E3 ubiquitin protein ligase | | RNF19A | 0.00011 | -1.66 |
| 215028_at | sema domain, transmembrane domain (TM), and cytoplasmic domain, (semaphorin) 6A | | SEMA6A | 0.00012 | -2.21 |
| 241935_at | shroom family member 1 | | SHROOM1 | 0.00037 | -1.83 |
| 216654_at | tenascin XB | | TNXB | 0.00096 | -1.23 |
| 216210_x_at | TRIO and F-actin binding protein | | TRIOBP | 0.00098 | -2.40 |
| 224562_at | WAS protein family, member 2 | | WASF2 | 0.00003 | -2.42 |
| 221725_at | WAS protein family, member 2 | | WASF2 | 0.00000 | -1.73 |
|  |  | |  |  |  |
| *Plasma membrane* | | |  |  |  |
| 242553_at | ATP-binding cassette, sub-family C (CFTR/MRP), member 3 | | ABCC3 | 0.00028 | -1.85 |
| 235277_at | angiomotin like 1 | | AMOTL1 | 0.00083 | -1.40 |
| 1555536_at | anthrax toxin receptor 2 | | ANTXR2 | 0.00055 | -1.32 |
| 1558792_x_at | adaptor-related protein complex 2, alpha 1 subunit | | AP2A1 | 0.00076 | -1.72 |
| 1560445_x_at | Rho guanine nucleotide exchange factor (GEF) 1 | | ARHGEF1 | 0.00046 | -2.01 |
| 228444_at | arrestin, beta 1 | | ARRB1 | 0.00074 | -1.76 |
| 209502_s_at | BAI1-associated protein 2 | | BAIAP2 | 0.00088 | -1.23 |
| 207050_at | calcium channel, voltage-dependent, alpha 2/delta subunit 1 | | CACNA2D1 | 0.00029 | -1.41 |
| 224137_at | calcium channel, voltage-dependent, gamma subunit 7 | | CACNG7 | 0.00066 | -1.26 |
| 207887_s_at | calcitonin receptor | | CALCR | 0.00076 | -1.40 |
| 38521_at | CD22 molecule | | CD22 | 0.00085 | -1.56 |
| 209543_s_at | CD34 molecule | | CD34 | 0.00069 | -1.69 |
| 236923_x_at | CD36 molecule (thrombospondin receptor) | | CD36 | 0.00045 | -1.71 |
| 1555748_x_at | CD79b molecule, immunoglobulin-associated beta | | CD79B | 0.00028 | -1.75 |
| 204677_at | cadherin 5, type 2 (vascular endothelium) | | CDH5 | 0.00087 | -1.92 |
| 219796_s_at | cadherin-related family member 5 | | CDHR5 | 0.00006 | -1.54 |
| 220496_at | C-type lectin domain family 1, member B | | CLEC1B | 0.00057 | -1.48 |
| 208488_s_at | complement component (3b/4b) receptor 1 (Knops blood group) | | CR1 | 0.00026 | -1.43 |
| 232609_at | crumbs homolog 3 (Drosophila) | | CRB3 | 0.00023 | -1.88 |
| 233315_at | C-terminal binding protein 2 | | CTBP1 | 0.00026 | -1.43 |
| 211130_x_at | ectodysplasin A | | EDA | 0.00000 | -1.77 |
| 237533_at | ecto-NOX disulfide-thiol exchanger 1 | | ENOX1 | 0.00073 | -2.01 |
| 233699_at | EPH receptor B2 | | EPHB2 | 0.00009 | -1.44 |
| 224401_s_at | Fc receptor-like 4 | | FCRL4 | 0.00040 | -1.51 |
| 224406_s_at | Fc receptor-like 5 | | FCRL5 | 0.00029 | -1.46 |
| 217487_x_at | folate hydrolase (prostate-specific membrane antigen) 1 /// folate hydrolase 1B | | FOLH1 /// FOLH1B | 0.00093 | -1.41 |
| 243265_at | glutamate decarboxylase 2 (pancreatic islets and brain, 65kDa) | | GAD2 | 0.00014 | -1.46 |
| 214302_x_at | gap junction protein, gamma 2, 47kDa | | GJC2 | 0.00008 | -1.49 |
| 230606_at | gap junction protein, delta 3, 31.9kDa | | GJD3 | 0.00062 | -1.26 |
| 239037_at | GNAS complex locus | | GNAS | 0.00014 | -1.86 |
| 215387_x_at | glypican 6 | | GOC6 | 0.00052 | -2.16 |
| 238062_at | glycosylphosphatidylinositol anchored high density lipoprotein binding protein 1 | | GPIHBP1 | 0.00017 | -1.76 |
| 220901_at | G protein-coupled receptor 157 | | GPR157 | 0.00031 | -1.78 |
| 232350_x_at | G protein-coupled receptor 161 | | GPR161 | 0.00029 | -1.57 |
| 208600_s_at | G protein-coupled receptor 39 | | GPR39 | 0.00041 | -1.57 |
| 210411_s_at | glutamate receptor, ionotropic, N-methyl D-aspartate 2B | | GRIN2B | 0.00096 | -1.50 |
| 238003_at | hepatic and glial cell adhesion molecule /// hepatocellular carcinoma, down-regulated 1 | | HEPACAM /// HEPN1 | 0.00001 | -2.26 |
| 217456_x_at | major histocompatibility complex, class I, E | | HLA-E | 0.00007 | -1.88 |
| 200905_x_at | major histocompatibility complex, class I, E | | HLA-E | 0.00046 | -1.90 |
| 217436_x_at | major histocompatibility complex, class I, J (pseudogene) | | HLA-J | 0.00032 | -1.39 |
| 233605_x_at | heterogeneous nuclear ribonucleoprotein M | | HNRNPM | 0.00096 | -1.51 |
| 215489_x_at | homer homolog 3 (Drosophila) | | HOMER3 | 0.00083 | -1.69 |
| 203308_x_at | Hermansky-Pudlak syndrome 1 | | HPS1 | 0.00022 | -1.40 |
| 220081_x_at | hydroxysteroid (17-beta) dehydrogenase 7 | | HSD17B7 | 0.00081 | -1.63 |
| 216939_s_at | 5-hydroxytryptamine (serotonin) receptor 4, G protein-coupled | | HTR4 | 0.00001 | -1.37 |
| 228178_s_at | hyaluronan synthase 3 | | HYAS3 | 0.00023 | -1.53 |
| 217212_s_at | interleukin 9 receptor | | IL9R | 0.00084 | -1.49 |
| 201015_s_at | junction plakoglobin | | JUP | 0.00020 | -1.76 |
| 221023_s_at | potassium voltage-gated channel, subfamily H (eag-related), member 6 | | KCNH6 | 0.00028 | -1.57 |
| 228581_at | potassium inwardly-rectifying channel, subfamily J, member 10 | | KCNJ10 | 0.00024 | -2.32 |
| 208404_x_at | potassium inwardly-rectifying channel, subfamily J, member 5 | | KCNJ5 | 0.00020 | -1.61 |
| 204486_at | KCNQ1 opposite strand/antisense transcript 1 (non-protein coding) | | KCNQ1OT1 | 0.00026 | -1.41 |
| 211768_at | linker for activation of T cells family, member 2 | | LAT2 | 0.00004 | -1.47 |
| 232716_at | lysophosphatidic acid receptor 1 | | LPA1 | 0.00020 | -2.73 |
| 205953_at | leucine-rich repeats and immunoglobulin-like domains 2 | | LRIG2 | 0.00037 | -1.20 |
| 204952_at | LY6/PLAUR domain containing 3 | | LYPD3 | 0.00017 | -1.45 |
| 225408_at | myelin basic protein | | MBP | 0.00095 | -1.38 |
| 236517_at | multiple EGF-like-domains 10 | | MEGF10 | 0.00058 | -1.93 |
| 232523_at | multiple EGF-like-domains 10 | | MEGF10 | 0.00088 | -2.17 |
| 1560334_at | multiple EGF-like-domains 11 | | MEGF11 | 0.00049 | -1.41 |
| 1569300_at | microfibrillar-associated protein 3-like | | MFAP3L | 0.00091 | -1.43 |
| 1560617_at | melanocortin 2 receptor accessory protein | | MRAP | 0.00001 | -1.46 |
| 227241_at | mucin 15, cell surface associated | | MUC15 | 0.00015 | -1.25 |
| 216660_at | myosin VIIB | | MYO7B | 0.00096 | -1.72 |
| 207380_x_at | NADPH oxidase 1 | | NOX1 | 0.00013 | -1.45 |
| 210808_s_at | NADPH oxidase 1 | | NOX1 | 0.00083 | -1.31 |
| 214680_at | neurotrophic tyrosine kinase, receptor, type 2 | | NTRK2 | 0.00024 | -1.51 |
| 208587_s_at | olfactory receptor, family 1, subfamily E, member 1 /// olfactory receptor, family 1, subfamily E, member 2 | | OR1E1 /// OR1E2 | 0.00076 | -1.27 |
| 214637_at | oncostatin M | | OSM | 0.00074 | -1.46 |
| 244471_x_at | pannexin 2 | | PANX2 | 0.00011 | -1.49 |
| 227626_at | progestin and adipoQ receptor family member VIII | | PAQR8 | 0.00028 | -1.60 |
| 221526_x_at | par-3 partitioning defective 3 homolog (C. elegans) | | PARD3 | 0.00035 | -2.15 |
| 210094_s_at | par-3 partitioning defective 3 homolog (C. elegans) | | PARD3 | 0.00086 | -2.69 |
| 1553190_s_at | par-3 partitioning defective 3 homolog B (C. elegans) | | PARD3B | 0.00014 | -1.55 |
| 219737_s_at | protocadherin 9 | | PCDH9 | 0.00054 | -1.44 |
| 1565601_at | protocadherin 9 | | PCDH9 | 0.00047 | -1.34 |
| 208205_at | protocadherin alpha 9 | | PCDHA9 | 0.00027 | -1.66 |
| 214564_s_at | protocadherin gamma subfamily C, 3 | | PCDHGC3 | 0.00040 | -1.44 |
| 177_at | phospholipase D1, phosphatidylcholine-specific | | PLD1 | 0.00011 | -1.26 |
| 221537_at | plexin A1 | | PLXNA1 | 0.00065 | -1.70 |
| 215668_s_at | plexin B1 | | PLXNB1 | 0.00042 | -1.40 |
| 239528_at | prominin 2 | | PROM2 | 0.00006 | -1.63 |
| 1555097_a_at | prostaglandin F receptor (FP) | | PTGFR | 0.00094 | -1.29 |
| 205911_at | parathyroid hormone 1 receptor | | PTH1R | 0.00015 | -1.45 |
| 211600_at | protein tyrosine phosphatase, receptor type, O | | PTPRO | 0.00070 | -3.39 |
| 241938_at | QKI, KH domain containing, RNA binding | | QKI | 0.00059 | -3.71 |
| 212636_at | QKI, KH domain containing, RNA binding | | QKI | 0.00001 | -1.85 |
| 217793_at | RAB11B, member RAS oncogene family | | RAB11B | 0.00096 | -1.74 |
| 233024_at | RAB18, member RAS oncogene family | | RAB18 | 0.00096 | -1.58 |
| 215507_x_at | RAB22A, member RAS oncogene family | | RAB22A | 0.00066 | -1.49 |
| 231000_at | receptor tyrosine kinase-like orphan receptor 2 | | ROR2 | 0.00033 | -1.51 |
| 205697_at | secretagogin, EF-hand calcium binding protein | | SCGN | 0.00069 | -1.49 |
| 222717_at | serum deprivation response | | SDPR | 0.00009 | -2.39 |
| 1556416_s_at | SEC31 homolog A (S. cerevisiae) | | SEC31A | 0.00055 | -1.68 |
| 210632_s_at | sarcoglycan, alpha (50kDa dystrophin-associated glycoprotein) | | SGCA | 0.00057 | -1.77 |
| 227506_at | solute carrier family 16, member 9 (monocarboxylic acid transporter 9) | | SLC16A9 | 0.00000 | -2.85 |
| 1569926_s_at | solute carrier family 34 (sodium phosphate), member 3 | | SLC34A3 | 0.00061 | -1.41 |
| 234973_at | solute carrier family 38, member 5 | | SLC38A5 | 0.00042 | -1.43 |
| 215401_at | solute carrier family 7, member 1 | | SLC7A1 | 0.00021 | -1.46 |
| 203516_at | syntrophin, alpha 1 | | SNTA1 | 0.00016 | -1.83 |
| 201060_x_at | stomatin | | STOM | 0.00022 | -1.97 |
| 202720_at | testis derived transcript (3 LIM domains) | | TES | 0.00053 | -1.67 |
| 217853_at | tensin 3 | | TNS3 | 0.00033 | -2.49 |
| 219360_s_at | transient receptor potential cation channel, subfamily M, member 4 | | TRPM4 | 0.00082 | -1.69 |
| 206827_s_at | transient receptor potential cation channel, subfamily V, member 6 | | TRPV6 | 0.00070 | -1.75 |
| 209263_x_at | tetraspanin 4 | | TSPAN4 | 0.00033 | -1.70 |
| 205854_at | tubby like protein 3 | | TULP3 | 0.00002 | -2.12 |
| 221291_at | UL16 binding protein 2 | | ULBP2 | 0.00012 | -1.57 |
| 238248_at | uromodulin | | UMOD | 0.00035 | -1.41 |
| 207263_x_at | vezatin, adherens junctions transmembrane protein | | VEZT | 0.00042 | -1.76 |
|  |  | |  |  |  |
| *Transcription* |  | |  |  |  |
| 1554487_a_at | activating transcription factor 6 beta | | ATF6B | 0.00023 | -1.87 |
| 239003_at | bromodomain and WD repeat domain containing 1 | | BRWD1 | 0.00043 | -2.61 |
| 220015_at | castor zinc finger 1 | | CASZ1 | 0.00056 | -1.57 |
| 225081_s_at | cell division cycle associated 7-like | | CDCA7L | 0.00004 | -2.47 |
| 229586_at | chromodomain helicase DNA binding protein 9 | | CHD9 | 0.00089 | -2.10 |
| 233345_at | EF-hand calcium binding domain 6 | | EFCAB6 | 0.00040 | -1.61 |
| 233244_at | eukaryotic translation initiation factor 2C, 2 | | EIF2C2 | 0.00069 | -1.60 |
| 235623_at | elongator acetyltransferase complex subunit 2 | | ELP2 | 0.00042 | -1.71 |
| 200878_at | endothelial PAS domain protein 1 | | EPAS1 | 0.00097 | -3.30 |
| 223937_at | forkhead box P1 | | FOXP1 | 0.00011 | -1.63 |
| 209710_at | GATA binding protein 2 | | GATA2 | 0.00054 | -1.92 |
| 230855_at | GATA binding protein 4 | | GATA4 | 0.00058 | -1.67 |
| 222104_x_at | general transcription factor IIH, polypeptide 3, 34kDa | | GTF2H3 | 0.00094 | -2.69 |
| 237868_x_at | general transcription factor IIH, polypeptide 5 | | GTF2H5 | 0.00094 | -2.18 |
| 236645_at | HMG-box transcription factor 1 | | HBP1 | 0.00053 | -2.28 |
| 1555665_at | hepatoma-derived growth factor | | HDGF | 0.00033 | -1.75 |
| 227347_x_at | hairy and enhancer of split 4 (Drosophila) | | HES4 | 0.00031 | -1.73 |
| 215933_s_at | hematopoietically expressed homeobox | | HHEX | 0.00057 | -1.32 |
| 214851_at | hepatocyte nuclear factor 4, alpha | | HNF4A | 0.00078 | -1.51 |
| 208604_s_at | homeobox A3 | | HOXA3 | 0.00006 | -1.73 |
| 1569501_at | heat shock transcription factor 1 | | HSF1 | 0.00034 | -1.52 |
| 239172_x_at | myoD family inhibitor domain containing | | MDFIC | 0.00028 | -1.31 |
| 215371_at | mediator complex subunit 27 | | MED27 | 0.00057 | -1.39 |
| 208196_x_at | nuclear factor of activated T-cells, cytoplasmic, calcineurin-dependent 1 | | NFATC1 | 0.00093 | -1.73 |
| 229834_at | nuclear factor I/X (CCAAT-binding transcription factor) | | NFIX | 0.00051 | -1.36 |
| 207535_s_at | nuclear factor of kappa light polypeptide gene enhancer in B-cells 2 (p49/p100) | | NFKB2 | 0.00099 | -1.51 |
| 241630_at | nuclear transcription factor Y, gamma | | NFYC | 0.00087 | -1.64 |
| 229281_at | neuronal PAS domain protein 3 | | NPAS3 | 0.00010 | -1.88 |
| 232700_at | nuclear respiratory factor | | NRF1 | 0.00053 | -1.92 |
| 228170_at | oligodendrocyte transcription factor 1 | | OLIG1 | 0.00053 | -1.88 |
| 211413_s_at | peptidyl arginine deiminase, type IV | | PADI4 | 0.00081 | -1.28 |
| 205646_s_at | paired box 6 | | PAX6 | 0.00022 | -2.08 |
| 1563241_at | PHD finger protein 12 | | PHF12 | 0.00013 | -1.73 |
| 225533_at | PHD finger protein 19 | | PHF19 | 0.00051 | -1.66 |
| 215718_s_at | PHD finger protein 3 | | PHF3 | 0.00060 | -1.71 |
| 225678_at | polymerase (RNA) III (DNA directed) polypeptide H (22.9kD) | | POLR3H | 0.00062 | -1.23 |
| 208286_x_at | POU class 5 homeobox 1 /// POU class 5 homeobox 1B /// POU class 5 homeobox 1 pseudogene 3 /// POU class 5 homeobox 1 pseudogene 4 | | POU5F1 /// POU5F1B /// POU5F1P3 /// POU5F1P4 | 0.00035 | -1.45 |
| 226978_at | peroxisome proliferator-activated receptor alpha | | PPARA | 0.00095 | -1.88 |
| 208044_s_at | peroxisome proliferator-activated receptor delta | | PPARD | 0.00031 | -1.81 |
| 239635_at | RNA binding motif protein 14 | | RBM14 | 0.00084 | -1.83 |
| 208133_at | replication factor C (activator 1) 1, 145kDa | | RFC1 | 0.00063 | -1.40 |
| 1556471_at | sex comb on midleg-like 4 (Drosophila) | | SCML4 | 0.00038 | -1.51 |
| 228898_s_at | SWI/SNF related, matrix associated, actin dependent regulator of chromatin, subfamily b, member 1 | | SMARCB1 | 0.00019 | -1.53 |
| 214404_x_at | SAM pointed domain containing ets transcription factor | | SPDEF | 0.00045 | -1.50 |
| 208528_x_at | synovial sarcoma, X breakpoint 5 | | SSX5 | 0.00021 | -1.58 |
| 213400_s_at | transducin (beta)-like 1X-linked | | TBL1X | 0.00048 | -2.48 |
| 208986_at | transcription factor 12 | | TCF12 | 0.00027 | -1.94 |
| 210776_x_at | transcription factor 3 | | TCF3 | 0.00059 | -1.34 |
| 1566932_x_at | transcription factor B2, mitochondrial | | TFB2M | 0.00100 | -1.67 |
| 205383_s_at | zinc finger and BTB domain containing 20 | | ZBTB20 | 0.00051 | -3.00 |
| 235308_at | zinc finger and BTB domain containing 20 | | ZBTB20 | 0.00003 | -3.67 |
| 233296_x_at | zinc finger and BTB domain containing 20 | | ZBTB20 | 0.00015 | -2.09 |
| 234338_s_at | zinc finger and BTB domain containing 47 | | ZBTB47 | 0.00009 | -1.33 |
| 206416_at | zinc finger protein 205 | | ZNF205 | 0.00048 | -1.84 |
| 215892_at | zinc finger protein 440 | | ZNF440 | 0.00041 | -1.62 |
| 1554160_a_at | zinc finger protein 446 | | ZNF446 | 0.00007 | -1.59 |
| 232029_at | zinc finger protein 451 | | ZNF451 | 0.00005 | -1.47 |
| 213641_at | zinc finger protein 500 | | ZNF500 | 0.00088 | -2.24 |
| 244466_at | zinc finger protein 544 | | ZNF544 | 0.00005 | -1.54 |
| 220242_x_at | zinc finger protein 701 | | ZNF701 | 0.00038 | -1.64 |
| 1559843_s_at | zinc finger protein 721 | | ZNF721 | 0.00014 | -1.29 |
| 1554476_x_at | zinc finger protein 808 | | ZNF808 | 0.00041 | -1.98 |
|  |  | |  |  |  |
| Miscellaneous/Unknown | | |  |  |  |
| 214763_at | acyl-CoA thioesterase 11 | | ACOT11 | 0.00077 | -1.59 |
| 220061_at | acyl-CoA synthetase medium-chain family member 5 | | ACSM5 | 0.00014 | -1.74 |
| 232570_s_at | ADAM metallopeptidase domain 33 | | ADAM33 | 0.00004 | -2.06 |
| 230167_at | ADAM metallopeptidase with thrombospondin type 1 motif, 14 | | ADAMTS14 | 0.00083 | -1.67 |
| 237437_s_at | adenosine deaminase, RNA-specific, B2 (non-functional) | | ADARB2 | 0.00001 | -2.15 |
| 223781_x_at | alcohol dehydrogenase 4 (class II), pi polypeptide | | ADH4 | 0.00010 | -2.13 |
| 225059_at | angiotensin II receptor-associated protein | | AGTRAP | 0.00079 | -1.46 |
| 211986_at | AHNAK nucleoprotein | | AHNAK | 0.00079 | -2.66 |
| 210625_s_at | A kinase (PRKA) anchor protein 1 | | AKAP1 | 0.00086 | -1.63 |
| 207015_s_at | aldehyde dehydrogenase 1 family, member A2 | | ALDH1A2 | 0.00032 | -1.63 |
| 224667_x_at | anaphase promoting complex subunit 16 | | ANAPC16 | 0.00089 | -1.98 |
| 219803_at | angiopoietin-like 3 | | ANGPTL3 | 0.00000 | -1.25 |
| 233895_at | ankyrin repeat domain 24 | | ANKRD24 | 0.00045 | -1.61 |
| 201305_x_at | acidic (leucine-rich) nuclear phosphoprotein 32 family, member B | | ANP32B | 0.00056 | -2.18 |
| 231903_x_at | Rho GTPase activating protein 23 | | ARHGAP23 | 0.00058 | -1.42 |
| 233903_s_at | Rho guanine nucleotide exchange factor (GEF) 26 | | ARHGEF26 | 0.00096 | -1.68 |
| 226055_at | arrestin domain containing 2 | | ARRDC2 | 0.00024 | -1.74 |
| 210551_s_at | acetylserotonin O-methyltransferase | | ASMT | 0.00059 | -1.52 |
| 1553567_s_at | ATP synthase F0 subunit 6 | | ATP6 | 0.00078 | -2.30 |
| 204378_at | breast carcinoma amplified sequence 1 | | BCAS1 | 0.00070 | -2.34 |
| 215654_at | branched chain amino-acid transaminase 2, mitochondrial | | BCAT2 | 0.00019 | -1.81 |
| 216397_s_at | block of proliferation 1 | | BOP1 | 0.00019 | -1.50 |
| 244622_at | bromodomain and WD repeat domain containing 1 | | BRWD1 | 0.00049 | -1.58 |
| 224373_s_at | chromosome 10 open reading frame 99 /// coiled-coil domain containing 104 /// heterogeneous nuclear ribonucleoprotein M /// NADH dehydrogenase, subunit 4 (complex I) | | C10orf99 /// CCDC104 /// HNRNPM /// ND4 | 0.00049 | -1.92 |
| 1554188_at | chromosome 11 open reading frame 53 | | C11orf53 | 0.00017 | -1.51 |
| 227292_at | chromosome 11 open reading frame 84 | | C11orf84 | 0.00077 | -1.50 |
| 1561721_a_at | chromosome 12 open reading frame 40 | | C12orf40 | 0.00065 | -1.35 |
| 217188_s_at | chromosome 14 open reading frame 1 | | C14orf1 | 0.00088 | -1.26 |
| 1554466_a_at | chromosome 16 open reading frame 13 | | C16orf13 | 0.00073 | -1.29 |
| 1557346_a_at | chromosome 17 open reading frame 51 | | C17orf51 | 0.00040 | -1.55 |
| 1554475_a_at | chromosome 19 open reading frame 47 | | C19orf47 | 0.00072 | -1.67 |
| 1554657_a_at | chromosome 20 open reading frame 26 | | C20orf26 | 0.00097 | -1.42 |
| 223697_x_at | chromosome 9 open reading frame 64 | | C9orf64 | 0.00097 | -2.45 |
| 219223_at | calcium channel flower domain containing 1 | | CACFD1 | 0.00052 | -1.70 |
| 228984_at | carnosine synthase 1 | | CARNS1 | 0.00034 | -1.87 |
| 220593_s_at | coiled-coil domain containing 40 | | CCDC40 | 0.00096 | -2.08 |
| 1554117_at | coiled-coil domain containing 60 | | CCDC60 | 0.00005 | -1.46 |
| 219025_at | CD248 molecule, endosialin | | CD248 | 0.00027 | -2.06 |
| 203507_at | CD68 molecule /// small nucleolar RNA, H/ACA box 67 | | CD68 /// SNORA67 | 0.00008 | -1.46 |
| 207188_at | cyclin-dependent kinase 3 /// TEN1-CDK3 readthrough (NMD candidate) | | CDK3 /// TEN1-CDK3 | 0.00090 | -1.45 |
| 1553158_at | centrosomal protein 19kDa | | CEP19 | 0.00085 | -1.24 |
| 206397_x_at | ceramide synthase 1 /// growth differentiation factor 1 | | CERS1 /// GDF1 | 0.00004 | -2.39 |
| 1559591_s_at | choline dehydrogenase | | CHDH | 0.00010 | -1.58 |
| 221961_at | chloride channel, voltage-sensitive 7 | | CLCN7 | 0.00028 | -2.00 |
| 219621_at | claspin | | CLSPN | 0.00081 | -1.34 |
| 1560754_at | CKLF-like MARVEL transmembrane domain containing 7 | | CMTM7 | 0.00012 | -1.75 |
| 52651_at | collagen, type VIII, alpha 2 | | COL8A2 | 0.00042 | -1.53 |
| 1553569_at | cytochrome c oxidase subunit II /// OAF homolog (Drosophila) /// transducin-like enhancer of split 1 (E(sp1) homolog, Drosophila) | | COX2 /// OAF /// TLE1 | 0.00080 | -2.52 |
| 1569293_x_at | ciliary rootlet coiled-coil, rootletin pseudogene 2 | | CROCCP2 | 0.00038 | -1.46 |
| 1554767_s_at | crystallin, zeta (quinone reductase)-like 1 | | CRYZL1 | 0.00068 | -1.27 |
| 217468_at | cytochrome P450, family 2, subfamily D, polypeptide 6 | | CYP2D6 | 0.00054 | -1.70 |
| 232516_x_at | death associated protein 3 | | DAP3 | 0.00024 | -2.07 |
| 229813_x_at | DAZ associated protein 1 | | DAZAP1 | 0.00086 | -1.40 |
| 211813_x_at | decorin | | DCN | 0.00018 | -1.79 |
| 227457_at | DET1 and DDB1 associated 1 | | DDA1 | 0.00028 | -1.45 |
| 207418_s_at | D-aspartate oxidase | | DDO | 0.00075 | -1.35 |
| 216459_x_at | DDR1 antisense RNA 1 (head to head) | | DDR1-AS1 | 0.00023 | -2.10 |
| 232915_at | DEAD (Asp-Glu-Ala-Asp) box polypeptide 49 | | DDX49 | 0.00062 | -1.65 |
| 229721_x_at | derlin 3 | | DERL3 | 0.00057 | -1.49 |
| 221887_s_at | deafness, autosomal recessive 31 | | DFNB31 | 0.00016 | -1.54 |
| 1557451_at | DiGeorge syndrome critical region gene 10 (non-protein coding) | | DGCR10 | 0.00070 | -1.69 |
| 241727_x_at | dihydrofolate reductase-like 1 | | DHFRL1 | 0.00031 | -1.85 |
| 233056_x_at | discs, large (Drosophila) homolog-associated protein 4 | | DLGAP4 | 0.00092 | -2.85 |
| 208216_at | distal-less homeobox 4 | | DLX4 | 0.00067 | -1.67 |
| 219651_at | developmental pluripotency associated 4 | | DPPA4 | 0.00026 | -1.38 |
| 224100_s_at | dihydropyrimidinase-like 5 | | DPYSL5 | 0.00042 | -1.25 |
| 211079_s_at | dual-specificity tyrosine-(Y)-phosphorylation regulated kinase 1A | | DYRK1A | 0.00099 | -1.37 |
| 1560874_at | EF-hand calcium binding domain 4B | | EFCAB4B | 0.00042 | -1.44 |
| 222314_x_at | eosinophil granule ontogeny transcript (non-protein coding) | | EGOT | 0.00074 | -1.80 |
| 236274_at | eukaryotic translation initiation factor 3, subunit B | | EIF3B | 0.00013 | -1.66 |
| 219599_at | eukaryotic translation initiation factor 4B | | EIF4B | 0.00009 | -2.56 |
| 201726_at | ELAV (embryonic lethal, abnormal vision, Drosophila)-like 1 (Hu antigen R) | | ELAVL1 | 0.00010 | -2.13 |
| 213779_at | EMI domain containing 1 | | EMID1 | 0.00048 | -1.76 |
| 232531_at | EMX2 opposite strand/antisense RNA | | EMX2OS | 0.00084 | -2.28 |
| 230430_at | ectonucleoside triphosphate diphosphohydrolase 2 | | ENTPD2 | 0.00009 | -1.66 |
| 229998_x_at | eva-1 homolog B (C. elegans) | | EVA1B | 0.00080 | -1.86 |
| 220838_at | exonuclease 3'-5' domain containing 3 | | EXD3 | 0.00011 | -1.41 |
| 223489_x_at | exosome component 3 | | EXOSC3 | 0.00032 | -1.47 |
| 226504_at | family with sequence similarity 109, member B | | FAM109B | 0.00010 | -1.34 |
| 242259_at | family with sequence similarity 187, member B | | FAM187B | 0.00042 | -1.27 |
| 221856_s_at | family with sequence similarity 63, member A | | FAM63A | 0.00015 | -2.01 |
| 1557218_s_at | Fanconi anemia, complementation group B | | FANCB | 0.00070 | -1.63 |
| 220164_s_at | F-box protein 40 | | FBXO40 | 0.00073 | -1.70 |
| 232064_at | fer (fps/fes related) tyrosine kinase | | FER | 0.00091 | -1.85 |
| 221376_at | fibroblast growth factor 17 | | FGF17 | 0.00018 | -1.68 |
| 238943_at | fibrinogen C domain containing 1 | | FIBCD1 | 0.00026 | -1.70 |
| 221120_at | --- | | FLJ20306 | 0.00056 | -1.96 |
| 1554286_at | MAP/microtubule affinity-regulating kinase 1 pseudogene | | FLJ25758 | 0.00031 | -1.69 |
| 220352_x_at | uncharacterized LOC645644 | | FLJ42627 | 0.00012 | -2.00 |
| 238986_at | uncharacterized LOC378805 | | FLJ43663 | 0.00071 | -1.31 |
| 238678_at | uncharacterized LOC402483 | | FLJ45340 | 0.00098 | -1.53 |
| 232096_x_at | FOXP1 intronic transcript 1 (non-protein coding) | | FOXP1-IT1 | 0.00013 | -1.79 |
| 223702_x_at | formimidoyltransferase cyclodeaminase | | FTCD | 0.00090 | -1.66 |
| 233741_at | formimidoyltransferase cyclodeaminase | | FTCD | 0.00040 | -1.45 |
| 242889_x_at | FUT8 antisense RNA 1 | | FUT8-AS1 | 0.00006 | -2.06 |
| 221759_at | glucose 6 phosphatase, catalytic, 3 | | G6PC3 | 0.00096 | -1.32 |
| 1563533_at | glutamate decarboxylase-like 1 | | GADL1 | 0.00060 | -2.49 |
| 211767_at | GINS complex subunit 4 (Sld5 homolog) | | GINS4 | 0.00090 | -1.38 |
| 222109_at | guanine nucleotide binding protein-like 3 (nucleolar)-like | | GNL3L | 0.00009 | -1.96 |
| 206747_at | G protein regulated inducer of neurite outgrowth 2 | | GPRIN2 | 0.00078 | -1.88 |
| 205752_s_at | glutathione S-transferase mu 5 | | GSTM5 | 0.00098 | -1.84 |
| 207003_at | guanylate cyclase activator 2A (guanylin) | | GUCA2A | 0.00058 | -1.46 |
| 238373_at | H1 histone family, member N, testis-specific | | H1FNT | 0.00082 | -1.91 |
| 216875_x_at | B1 for mucin | | HAB1 | 0.00022 | -1.34 |
| 220142_at | hyaluronan and proteoglycan link protein 2 | | HAPLN2 | 0.00071 | -1.90 |
| 233655_s_at | HAUS augmin-like complex, subunit 6 | | HAUS6 | 0.00009 | -1.25 |
| 223252_at | hepatoma-derived growth factor-related protein 2 | | HDGFRP2 | 0.00023 | -1.29 |
| 232627_at | hepatocyte growth factor-regulated tyrosine kinase substrate | | HGS | 0.00009 | -1.81 |
| 227614_at | hexokinase domain containing 1 | | HKDC1 | 0.00061 | -1.57 |
| 215536_at | major histocompatibility complex, class II, DQ beta 2 | | HLA-DQB2 | 0.00032 | -1.28 |
| 204111_at | histamine N-methyltransferase | | HNMT | 0.00057 | -1.17 |
| 211732_x_at | histamine N-methyltransferase | | HNMT | 0.00030 | -1.82 |
| 239093_at | 4-hydroxy-2-oxoglutarate aldolase 1 | | HOGA1 | 0.00016 | -2.11 |
| 221379_at | --- | | HUG1 | 0.00044 | -1.47 |
| 215490_at | IBA57, iron-sulfur cluster assembly homolog (S. cerevisiae) | | IBA57 | 0.00011 | -1.68 |
| 229386_at | inhibitor of DNA binding 4, dominant negative helix-loop-helix protein | | ID4 | 0.00021 | -1.45 |
| 217432_s_at | iduronate 2-sulfatase | | IDS | 0.00029 | -1.66 |
| 216829_at | immunoglobulin kappa locus /// immunoglobulin kappa constant | | IGK /// IGKC | 0.00073 | -1.39 |
| 214777_at | immunoglobulin kappa variable 4-1 /// NULL | | IGKV4-1 /// IGKV4-1 | 0.00074 | -1.65 |
| 1552672_a_at | immunoglobulin superfamily, member 3 | | IGSF3 | 0.00028 | -1.31 |
| 207252_at | inactivation escape 1 (non-protein coding) | | INE1 | 0.00006 | -1.30 |
| 1557770_at | importin 11 | | IPO11 | 0.00053 | -2.13 |
| 213005_s_at | KN motif and ankyrin repeat domains 1 | | KANK1 | 0.00073 | -2.57 |
| 216294_s_at | KIAA1109 | | KIAA1109 | 0.00055 | -1.34 |
| 1557601_s_at | KIAA1257 /// uncharacterized LOC100132731 | | KIAA1257 /// LOC100132731 | 0.00088 | -1.24 |
| 235021_at | KIAA2026 | | KIAA2026 | 0.00044 | -2.23 |
| 213656_s_at | kinesin light chain 1 | | KLC1 | 0.00060 | -2.41 |
| 239853_at | kinesin light chain 3 | | KLC3 | 0.00009 | -1.52 |
| 1554941_at | kelch-like family member 14 | | KLHL14 | 0.00027 | -1.24 |
| 217315_s_at | kallikrein-related peptidase 13 | | KLK13 | 0.00046 | -1.58 |
| 213235_at | lysine-rich nucleolar protein 1 | | KNOP1 | 0.00002 | -2.20 |
| 233439_at | leucine zipper-EF-hand containing transmembrane protein 1 | | LETM1 | 0.00031 | -1.31 |
| 219823_at | lin-28 homolog A (C. elegans) | | LIN28A | 0.00072 | -1.44 |
| 224443_at | long intergenic non-protein coding RNA 467 | | LINC00467 | 0.00088 | -2.26 |
| 1570345_at | long intergenic non-protein coding RNA 474 | | LINC00474 | 0.00022 | -1.38 |
| 1566526_at | long intergenic non-protein coding RNA 927 | | LINC00927 | 0.00023 | -1.49 |
| 1560819_a_at | long intergenic non-protein coding RNA 944 | | LINC00944 | 0.00009 | -1.33 |
| 1558728_at | uncharacterized LOC100128881 | | LOC100128881 | 0.00000 | -1.65 |
| 230574_at | uncharacterized LOC100130938 | | LOC100130938 | 0.00006 | -1.86 |
| 1563822_at | uncharacterized LOC100131763 | | LOC100131763 | 0.00066 | -1.42 |
| 1558527_at | uncharacterized LOC100132707 | | LOC100132707 | 0.00040 | -2.08 |
| 243083_at | uncharacterized LOC100287704 /// uncharacterized LOC100287834 | | LOC100287704 /// LOC100287834 | 0.00025 | -1.86 |
| 231677_at | uncharacterized LOC100506165 | | LOC100506165 | 0.00018 | -1.31 |
| 243710_at | uncharacterized LOC100506175 | | LOC100506175 | 0.00004 | -1.61 |
| 243510_at | uncharacterized LOC100506418 | | LOC100506418 | 0.00041 | -1.36 |
| 244037_at | uncharacterized LOC100507520 | | LOC100507520 | 0.00052 | -1.56 |
| 229469_at | G antigen family D member 3-like /// X antigen family, member 2 /// X antigen family, member 2B | | LOC101060193 /// XAGE2 /// XAGE2B | 0.00021 | -1.50 |
| 1562788_at | uncharacterized LOC254099 | | LOC254099 | 0.00006 | -1.38 |
| 1557591_at | uncharacterized LOC283038 | | LOC283038 | 0.00036 | -1.54 |
| 1555847_a_at | uncharacterized LOC284454 | | LOC284454 | 0.00092 | -1.93 |
| 1559083_x_at | uncharacterized LOC284600 | | LOC284600 | 0.00093 | -1.85 |
| 236846_at | uncharacterized LOC284757 | | LOC284757 | 0.00036 | -2.08 |
| 237163_x_at | protein phosphatase 2, regulatory subunit B'', beta pseudogene | | LOC390705 | 0.00031 | -1.36 |
| 1569436_at | uncharacterized LOC400128 | | LOC400128 | 0.00024 | -1.39 |
| 223911_at | uncharacterized LOC440330 | | LOC440330 | 0.00059 | -1.58 |
| 1562428_at | SFPQ | | LOC654780 | 0.00021 | -1.58 |
| 242835_s_at | uncharacterized LOC728730 | | LOC728730 | 0.00092 | -2.48 |
| 206792_x_at | uncharacterized LOC729966 /// phosphodiesterase 4C, cAMP-specific | | LOC729966 /// PDE4C | 0.00100 | -2.25 |
| 234664_at | uncharacterized LOC731275 | | LOC731275 | 0.00043 | -1.28 |
| 227252_at | low density lipoprotein receptor-related protein 10 | | LRP10 | 0.00001 | -2.21 |
| 212850_s_at | low density lipoprotein receptor-related protein 4 | | LRP4 | 0.00082 | -1.86 |
| 207790_at | leucine rich repeat containing 1 | | LRRC1 | 0.00073 | -1.46 |
| 235214_at | leucine rich adaptor protein 1 | | LURAP1 | 0.00047 | -1.57 |
| 215391_at | microtubule-associated protein 1A | | MAP1A | 0.00009 | -1.63 |
| 216206_x_at | mitogen-activated protein kinase kinase 7 | | MAP2K7 | 0.00069 | -2.12 |
| 243_g_at | microtubule-associated protein 4 | | MAP4 | 0.00021 | -1.58 |
| 242077_x_at | Mab-21 domain containing 1 | | MB21D1 | 0.00032 | -1.63 |
| 1553978_at | myocyte enhancer factor 2B /// MEF2B neighbor /// MEF2BNB-MEF2B readthrough | | MEF2B /// MEF2BNB /// MEF2BNB-MEF2B | 0.00007 | -1.89 |
| 212713_at | microfibrillar-associated protein 4 | | MFAP4 | 0.00033 | -1.39 |
| 225316_at | major facilitator superfamily domain containing 2A | | MFSD2A | 0.00026 | -1.73 |
| 232446_at | uncharacterized LOC90768 | | MGC45800 | 0.00009 | -1.18 |
| 243760_at | mitochondrial intermediate peptidase pseudogene 3 | | MIPEPP3 | 0.00042 | -1.68 |
| 228235_at | MIR503 host gene (non-protein coding) | | MIR503HG | 0.00055 | -1.50 |
| 227672_at | maestro heat-like repeat family member 6 | | MROH6 | 0.00084 | -1.57 |
| 229510_at | membrane-spanning 4-domains, subfamily A, member 14 | | MS4A14 | 0.00059 | -1.90 |
| 223311_s_at | metastasis associated 1 family, member 3 | | MTA3 | 0.00076 | -1.99 |
| 228593_at | myotubularin related protein 9-like, pseudogene | | MTMR9LP | 0.00041 | -2.07 |
| 227743_at | myosin XVB pseudogene | | MYO15B | 0.00036 | -1.64 |
| 217463_s_at | myelin regulatory factor | | MYRF | 0.00056 | -1.65 |
| 212993_at | NACC family member 2, BEN and BTB (POZ) domain containing | | NACC2 | 0.00001 | -2.27 |
| 213613_s_at | NAD kinase | | NADK | 0.00010 | -1.46 |
| 242880_at | sodium leak channel, non-selective | | NALCN | 0.00058 | -1.50 |
| 232828_at | NALCN antisense RNA 1 | | NALCN-AS1 | 0.00030 | -1.55 |
| 222598_s_at | neuron navigator 2 | | NAV2 | 0.00034 | -1.72 |
| 230190_at | Nedd4 family interacting protein 2 | | NDFIP2 | 0.00027 | -1.63 |
| 202608_s_at | N-deacetylase/N-sulfotransferase (heparan glucosaminyl) 1 | | NDST1 | 0.00054 | -1.56 |
| 227795_at | NADH dehydrogenase (ubiquinone) flavoprotein 1, 51kDa | | NDUFV1 | 0.00069 | -1.85 |
| 232593_at | neuralized homolog 3 (Drosophila) pseudogene | | NEURL3 | 0.00093 | -1.13 |
| 234922_s_at | NHS-like 1 | | NHSL1 | 0.00046 | -1.51 |
| 1557737_s_at | natural killer-tumor recognition sequence | | NKTR | 0.00006 | -2.10 |
| 206915_at | NK2 homeobox 2 | | NKX2-2 | 0.00096 | -2.03 |
| 1560204_at | 5'-nucleotidase domain containing 4 | | NT5DC4 | 0.00087 | -1.34 |
| 214251_s_at | nuclear mitotic apparatus protein 1 | | NUMA1 | 0.00099 | -1.29 |
| 212691_at | nucleoporin 188kDa | | NUP188 | 0.00074 | -1.48 |
| 225510_at | OAF homolog (Drosophila) | | OAF | 0.00033 | -1.65 |
| 233860_s_at | odorant binding protein 2A /// odorant binding protein 2B | | OBP2A /// OBP2B | 0.00088 | -1.35 |
| 221361_at | olfactory marker protein | | OMP | 0.00079 | -1.47 |
| 220256_s_at | 3-oxoacid CoA transferase 2 | | OXCT2 | 0.00082 | -1.74 |
| 224557_x_at | purinergic receptor P2X, ligand-gated ion channel, 2 | | P2RX2 | 0.00054 | -1.53 |
| 209154_at | P2RX5-TAX1BP3 readthrough /// Tax1 (human T-cell leukemia virus type I) binding protein 3 | | P2RX5-TAX1BP3 /// TAX1BP3 | 0.00053 | -2.54 |
| 233896_s_at | papilin, proteoglycan-like sulfated glycoprotein | | PAPLN | 0.00062 | -1.49 |
| 203060_s_at | 3'-phosphoadenosine 5'-phosphosulfate synthase 2 | | PAPSS2 | 0.00038 | -2.67 |
| 214177_s_at | pre-B-cell leukemia homeobox interacting protein 1 | | PBXIP1 | 0.00086 | -2.34 |
| 1552343_s_at | phosphodiesterase 7A | | PDE7A | 0.00011 | -1.74 |
| 212522_at | phosphodiesterase 8A | | PDE8A | 0.00030 | -1.75 |
| 1566670_at | pyridoxal (pyridoxine, vitamin B6) kinase | | PDXK | 0.00033 | -1.55 |
| 201397_at | phosphoglycerate dehydrogenase | | PHGDH | 0.00002 | -1.54 |
| 239568_at | pleckstrin homology domain containing, family H (with MyTH4 domain) member 2 | | PLEKHH2 | 0.00032 | -1.73 |
| 204436_at | pleckstrin homology domain containing, family O member 2 | | PLEKHO2 | 0.00003 | -1.79 |
| 241368_at | perilipin 5 | | PLIN5 | 0.00042 | -2.12 |
| 210722_at | pancreatic lipase-related protein 1 | | PNLIPRP1 | 0.00075 | -1.49 |
| 236135_at | patatin-like phospholipase domain containing 7 | | PNPLA7 | 0.00084 | -1.39 |
| 220411_x_at | podocan-like 1 | | PODNL1 | 0.00032 | -1.95 |
| 215281_x_at | pogo transposable element with ZNF domain | | POGZ | 0.00035 | -1.78 |
| 1565329_at | polymerase (DNA-directed), epsilon 4, accessory subunit | | POLE4 | 0.00053 | -1.55 |
| 219729_at | paired related homeobox 2 | | PRRX2 | 0.00038 | -1.61 |
| 223062_s_at | phosphoserine aminotransferase 1 | | PSAT1 | 0.00077 | -1.53 |
| 208549_x_at | prothymosin, alpha | | PTMA | 0.00016 | -1.50 |
| 212923_s_at | PX domain containing 1 | | PXDC1 | 0.00094 | -2.13 |
| 202148_s_at | pyrroline-5-carboxylate reductase 1 | | PYCR1 | 0.00043 | -2.05 |
| 231830_x_at | RAB11 family interacting protein 1 (class I) | | RAB11FIP1 | 0.00008 | -1.73 |
| 215070_x_at | RAB GTPase activating protein 1 | | RABGAP1 | 0.00082 | -1.57 |
| 210051_at | Rap guanine nucleotide exchange factor (GEF) 3 | | RAPGEF3 | 0.00025 | -2.64 |
| 219167_at | RAS-like, family 12 | | RASL12 | 0.00014 | -1.90 |
| 212430_at | RNA binding motif protein 38 | | RBM38 | 0.00043 | -1.63 |
| 209487_at | RNA binding protein with multiple splicing | | RBPMS | 0.00031 | -2.05 |
| 232359_at | retinol dehydrogenase 11 (all-trans/9-cis/11-cis) | | RDH11 | 0.00032 | -1.37 |
| 221686_s_at | RecQ protein-like 5 | | RECQL5 | 0.00020 | -1.44 |
| 228877_at | ral guanine nucleotide dissociation stimulator-like 3 | | RGL3 | 0.00026 | -2.07 |
| 217622_at | rhomboid domain containing 3 | | RHBDD3 | 0.00064 | -1.86 |
| 222711_s_at | rhomboid 5 homolog 1 (Drosophila) | | RHBDF1 | 0.00027 | -1.56 |
| 1568728_s_at | ring finger protein 207 | | RNF207 | 0.00038 | -1.60 |
| 244759_x_at | ring finger protein 207 | | RNF207 | 0.00081 | -1.61 |
| 219550_at | roundabout, axon guidance receptor, homolog 3 (Drosophila) | | ROBO3 | 0.00002 | -1.89 |
| 222297_x_at | ribosomal protein L18 pseudogene 10 /// NULL | | RPL18P10 /// RPL18P10 | 0.00033 | -1.50 |
| 201206_s_at | ribosome binding protein 1 | | RRBP1 | 0.00088 | -1.50 |
| 212389_at | SET binding factor 1 | | SBF1 | 0.00003 | -1.63 |
| 226373_at | sideroflexin 5 | | SFXN5 | 0.00014 | -1.75 |
| 242543_at | SH2 domain containing 6 | | SH2D6 | 0.00095 | -1.32 |
| 231701_s_at | serine hydroxymethyltransferase 1 (soluble) | | SHMT1 | 0.00011 | -1.71 |
| 207355_at | solute carrier family 1 (glutamate transporter), member 7 | | SLC1A7 | 0.00013 | -1.34 |
| 228236_at | solute carrier family 52, riboflavin transporter, member 3 | | SLC52A3 | 0.00018 | -1.33 |
| 203473_at | solute carrier organic anion transporter family, member 2B1 | | SLCO2B1 | 0.00029 | -1.72 |
| 236130_at | small nucleolar RNA, H/ACA box 37 | | SNORA37 | 0.00020 | -1.65 |
| 230509_at | sorting nexin 22 | | SNX22 | 0.00076 | -2.03 |
| 223241_at | sorting nexin 8 | | SNX8 | 0.00048 | -1.63 |
| 1570241_at | spermatogenesis associated 21 | | SPATA21 | 0.00000 | -1.75 |
| 222046_at | serrate RNA effector molecule homolog (Arabidopsis) | | SRRT | 0.00061 | -1.62 |
| 225034_at | ST3 beta-galactoside alpha-2,3-sialyltransferase 1 | | ST3GAL1 | 0.00078 | -1.48 |
| 216310_at | TAO kinase 1 | | TAOK1 | 0.00100 | -2.15 |
| 244615_x_at | threonyl-tRNA synthetase-like 2 | | TARSL2 | 0.00084 | -1.65 |
| 204043_at | transcobalamin II | | TCN2 | 0.00049 | -1.85 |
| 1554641_a_at | tet methylcytosine dioxygenase 3 | | TET3 | 0.00024 | -1.45 |
| 203092_at | translocase of inner mitochondrial membrane 44 homolog (yeast) | | TIMM44 | 0.00032 | -1.45 |
| 218745_x_at | transmembrane protein 161A | | TMEM161A | 0.00075 | -1.42 |
| 222892_s_at | transmembrane protein 40 | | TMEM40 | 0.00034 | -1.30 |
| 244716_x_at | transmembrane and immunoglobulin domain containing 2 | | TMIGD2 | 0.00013 | -1.75 |
| 1569986_x_at | troponin T type 3 (skeletal, fast) | | TNNT3 | 0.00021 | -1.54 |
| 205693_at | troponin T type 3 (skeletal, fast) | | TNNT3 | 0.00015 | -1.51 |
| 242364_x_at | TNRC6C antisense RNA 1 | | TNRC6C-AS1 | 0.00054 | -1.51 |
| 225891_at | taperin | | TPRN | 0.00026 | -1.62 |
| 207134_x_at | tryptase beta 2 (gene/pseudogene) | | TPSB2 | 0.00081 | -1.47 |
| 202368_s_at | translocation associated membrane protein 2 | | TRAM2 | 0.00021 | -1.54 |
| 204985_s_at | trafficking protein particle complex 6A | | TRAPPC6A | 0.00049 | -1.56 |
| 1565814_at | tripartite motif containing 36 | | TRIM36 | 0.00071 | -1.32 |
| 235084_x_at | tripartite motif containing 38 | | TRIM38 | 0.00036 | -2.04 |
| 221907_at | tRNA methyltransferase 61 homolog A (S. cerevisiae) | | TRMT61A | 0.00007 | -1.55 |
| 216434_at | tetratricopeptide repeat domain 38 | | TTC38 | 0.00023 | -1.59 |
| 209372_x_at | tubulin, beta 2A class IIa /// tubulin, beta 2B class IIb | | TUBB2A /// TUBB2B | 0.00099 | -1.66 |
| 225873_at | trypsin domain containing 1 | | TYSND1 | 0.00085 | -1.58 |
| 216584_at | ubiquitin-conjugating enzyme E2 variant 2 pseudgene 3 /// NULL | | UBE2V2P3 /// UBE2V2P3 | 0.00070 | -1.42 |
| 222252_x_at | ubiquilin 4 | | UBQLN4 | 0.00096 | -1.92 |
| 235327_x_at | UBX domain protein 2A | | UBXN2A | 0.00085 | -2.43 |
| 207499_x_at | unc-45 homolog A (C. elegans) | | UNC45A | 0.00001 | -1.71 |
| 1559324_at | ubiquitin specific peptidase 32 pseudogene 2 | | USP32P2 | 0.00070 | -1.37 |
| 207365_x_at | ubiquitin specific peptidase 34 | | USP34 | 0.00058 | -1.83 |
| 1555063_at | ubiquitin specific peptidase 6 (Tre-2 oncogene) | | USP6 | 0.00009 | -1.32 |
| 201796_s_at | valyl-tRNA synthetase | | VARS | 0.00025 | -1.52 |
| 203683_s_at | vascular endothelial growth factor B | | VEGFB | 0.00055 | -1.19 |
| 235237_at | VMA21 vacuolar H+-ATPase homolog (S. cerevisiae) | | VMA21 | 0.00037 | -1.52 |
| 1555314_at | WD repeat domain 19 | | WDR19 | 0.00047 | -1.96 |
| 242234_at | XIAP associated factor 1 | | XAF1 | 0.00012 | -1.62 |
| 206169_x_at | zinc finger CCCH-type containing 7B | | ZC3H7B | 0.00028 | -2.96 |
| 212419_at | zinc finger, CCHC domain containing 24 | | ZCCHC24 | 0.00002 | -3.08 |
| 212423_at | zinc finger, CCHC domain containing 24 | | ZCCHC24 | 0.00032 | -2.63 |
| 217486_s_at | zinc finger, DHHC-type containing 17 | | ZDHHC17 | 0.00050 | -2.95 |
| 213277_at | ZFP36 ring finger protein-like 1 | | ZFP36L1 | 0.00038 | -1.48 |
| 1569383_s_at | zinc finger, FYVE domain containing 28 | | ZFYVE28 | 0.00008 | -1.41 |
| 233399_x_at | zinc finger protein 252, pseudogene | | ZNF252P | 0.00010 | -1.62 |
| 1554972_at | --- | | --- | 0.00029 | -1.52 |
| 1556590_s_at | --- | | --- | 0.00085 | -2.15 |
| 1556937_at | --- | | --- | 0.00075 | -1.79 |
| 1557409_at | --- | | --- | 0.00081 | -1.36 |
| 1557457_at | --- | | --- | 0.00030 | -1.63 |
| 1557712_x_at | --- | | --- | 0.00008 | -1.81 |
| 1557987_at | --- | | --- | 0.00050 | -2.38 |
| 1559642_a_at | --- | | --- | 0.00067 | -1.56 |
| 1560086_at | --- | | --- | 0.00071 | -1.72 |
| 1560908_at | --- | | --- | 0.00066 | -1.77 |
| 1561087_at | --- | | --- | 0.00040 | -1.46 |
| 1561658_at | --- | | --- | 0.00057 | -3.28 |
| 1561870_at | --- | | --- | 0.00091 | -1.50 |
| 1562106_at | --- | | --- | 0.00098 | -1.67 |
| 1562300_at | --- | | --- | 0.00034 | -1.31 |
| 1563386_at | --- | | --- | 0.00053 | -2.12 |
| 1564379_at | --- | | --- | 0.00009 | -1.44 |
| 1564773_x_at | --- | | --- | 0.00088 | -2.29 |
| 1565705_x_at | --- | | --- | 0.00081 | -2.36 |
| 1565802_at | --- | | --- | 0.00007 | -1.49 |
| 1566003_x_at | --- | | --- | 0.00013 | -1.52 |
| 1566278_at | --- | | --- | 0.00014 | -1.99 |
| 1566763_at | --- | | --- | 0.00095 | -1.52 |
| 1566768_at | --- | | --- | 0.00017 | -1.61 |
| 1567167_at | --- | | --- | 0.00012 | -1.44 |
| 1570361_a_at | --- | | --- | 0.00052 | -1.52 |
| 1570432_at | --- | | --- | 0.00036 | -1.38 |
| 206548_at | --- | | --- | 0.00091 | -4.20 |
| 207436_x_at | --- | | --- | 0.00014 | -2.11 |
| 207730_x_at | --- | | --- | 0.00018 | -2.58 |
| 207756_at | --- | | --- | 0.00030 | -2.53 |
| 207953_at | --- | | --- | 0.00015 | -2.24 |
| 208139_s_at | --- | | --- | 0.00043 | -2.08 |
| 208185_x_at | --- | | --- | 0.00038 | -2.14 |
| 210920_x_at | --- | | --- | 0.00001 | -1.87 |
| 211374_x_at | --- | | --- | 0.00002 | -1.98 |
| 213156_at | --- | | --- | 0.00002 | -3.44 |
| 215137_at | --- | | --- | 0.00076 | -1.48 |
| 215197_at | --- | | --- | 0.00029 | -1.40 |
| 215360_at | --- | | --- | 0.00001 | -1.51 |
| 216158_at | --- | | --- | 0.00002 | -1.60 |
| 216745_x_at | --- | | --- | 0.00058 | -1.78 |
| 216751_at | --- | | --- | 0.00062 | -1.52 |
| 217396_at | --- | | --- | 0.00031 | -1.67 |
| 217446_x_at | --- | | --- | 0.00020 | -2.32 |
| 217474_at | --- | | --- | 0.00022 | -1.65 |
| 217570_x_at | --- | | --- | 0.00009 | -1.69 |
| 217579_x_at | --- | | --- | 0.00023 | -1.74 |
| 217679_x_at | --- | | --- | 0.00067 | -2.82 |
| 217713_x_at | --- | | --- | 0.00036 | -2.17 |
| 218279_s_at | --- | | --- | 0.00045 | -1.79 |
| 220296_at | --- | | --- | 0.00040 | -1.73 |
| 220743_at | --- | | --- | 0.00067 | -1.52 |
| 220905_at | --- | | --- | 0.00041 | -2.10 |
| 221071_at | --- | | --- | 0.00023 | -2.45 |
| 221155_x_at | --- | | --- | 0.00020 | -1.79 |
| 221176_x_at | --- | | --- | 0.00025 | -1.92 |
| 222207_x_at | --- | | --- | 0.00004 | -2.04 |
| 222284_at | --- | | --- | 0.00030 | -2.05 |
| 224067_at | --- | | --- | 0.00052 | -1.52 |
| 224087_at | --- | | --- | 0.00021 | -1.50 |
| 224105_x_at | --- | | --- | 0.00032 | -2.17 |
| 224214_at | --- | | --- | 0.00066 | -1.43 |
| 224261_at | --- | | --- | 0.00001 | -1.46 |
| 226252_at | --- | | --- | 0.00028 | -1.59 |
| 228159_at | --- | | --- | 0.00002 | -1.75 |
| 228934_x_at | --- | | --- | 0.00015 | -2.37 |
| 230711_at | --- | | --- | 0.00073 | -2.85 |
| 231191_at | --- | | --- | 0.00024 | -1.69 |
| 231212_x_at | --- | | --- | 0.00065 | -2.19 |
| 231322_at | --- | | --- | 0.00092 | -1.82 |
| 232699_at | --- | | --- | 0.00039 | -1.82 |
| 233041_x_at | --- | | --- | 0.00071 | -2.32 |
| 233055_at | --- | | --- | 0.00012 | -2.21 |
| 233103_at | --- | | --- | 0.00094 | -1.65 |
| 233180_at | --- | | --- | 0.00016 | -1.89 |
| 233282_at | --- | | --- | 0.00036 | -1.78 |
| 233427_x_at | --- | | --- | 0.00036 | -2.23 |
| 233431_x_at | --- | | --- | 0.00045 | -1.99 |
| 234046_at | --- | | --- | 0.00053 | -1.33 |
| 234158_at | --- | | --- | 0.00030 | -1.41 |
| 234181_at | --- | | --- | 0.00054 | -1.47 |
| 234438_at | --- | | --- | 0.00017 | -1.67 |
| 234565_x_at | --- | | --- | 0.00045 | -1.93 |
| 234612_at | --- | | --- | 0.00054 | -1.95 |
| 235842_at | --- | | --- | 0.00084 | -1.57 |
| 235875_at | --- | | --- | 0.00011 | -2.10 |
| 235986_at | --- | | --- | 0.00021 | -1.39 |
| 236015_at | --- | | --- | 0.00093 | -1.51 |
| 236147_at | --- | | --- | 0.00054 | -1.81 |
| 236438_at | --- | | --- | 0.00060 | -1.55 |
| 236764_at | --- | | --- | 0.00050 | -1.74 |
| 236818_at | --- | | --- | 0.00047 | -1.29 |
| 237078_at | --- | | --- | 0.00068 | -1.60 |
| 237232_at | --- | | --- | 0.00057 | -1.63 |
| 237352_at | --- | | --- | 0.00084 | -1.85 |
| 237436_at | --- | | --- | 0.00024 | -1.60 |
| 237562_at | --- | | --- | 0.00075 | -1.72 |
| 237769_at | --- | | --- | 0.00027 | -1.48 |
| 237962_x_at | --- | | --- | 0.00050 | -1.74 |
| 238643_at | --- | | --- | 0.00096 | -1.43 |
| 239306_at | --- | | --- | 0.00082 | -1.61 |
| 239658_at | --- | | --- | 0.00097 | -1.62 |
| 240238_at | --- | | --- | 0.00035 | -2.05 |
| 240481_at | --- | | --- | 0.00097 | -1.34 |
| 240775_at | --- | | --- | 0.00012 | -1.89 |
| 240813_at | --- | | --- | 0.00071 | -1.87 |
| 240849_at | --- | | --- | 0.00099 | -1.68 |
| 241203_at | --- | | --- | 0.00005 | -1.74 |
| 241243_at | --- | | --- | 0.00080 | -1.49 |
| 241250_at | --- | | --- | 0.00087 | -2.11 |
| 241255_at | --- | | --- | 0.00094 | -2.15 |
| 241848_x_at | --- | | --- | 0.00052 | -1.92 |
| 242182_x_at | --- | | --- | 0.00030 | -1.91 |
| 242475_at | --- | | --- | 0.00023 | -1.67 |
| 242509_at | --- | | --- | 0.00000 | -1.64 |
| 242589_x_at | --- | | --- | 0.00003 | -1.50 |
| 242681_at | --- | | --- | 0.00084 | -1.66 |
| 242829_x_at | --- | | --- | 0.00047 | -2.45 |
| 242846_at | --- | | --- | 0.00017 | -1.87 |
| 243014_at | --- | | --- | 0.00076 | -1.83 |
| 243147_x_at | --- | | --- | 0.00043 | -2.51 |
| 243416_at | --- | | --- | 0.00005 | -1.97 |
| 243763_x_at | --- | | --- | 0.00070 | -1.76 |
| 243955_at | --- | | --- | 0.00062 | -1.39 |
| 244188_at | --- | | --- | 0.00036 | -2.08 |
| 244326_at | --- | | --- | 0.00012 | -1.47 |
| 244340_x_at | --- | | --- | 0.00056 | -2.53 |
| 244448_at | --- | | --- | 0.00023 | -1.42 |
| 244562_s_at | --- | | --- | 0.00019 | -1.35 |
| 244658_at | --- | | --- | 0.00036 | -1.36 |
